# Lutein Isomers: Preparation, Separation, Structure Elucidation, and Occurrence in 20 Medicinal Plants

**DOI:** 10.3390/molecules28031187

**Published:** 2023-01-25

**Authors:** Veronika Nagy, Attila Agócs, Viktória L. Balázs, Dragica Purger, Rita Filep, Viktor Sándor, Erika Turcsi, Gergely Gulyás-Fekete, József Deli

**Affiliations:** 1 Department of Biochemistry and Medical Chemistry, Medical School, University of Pécs, Szigeti út 12., H-7624 Pécs, Hungary; 2 Department of Pharmacognosy, Faculty of Pharmacy, University of Pécs, Rókus u. 2., H-7624 Pécs, Hungary; 3 Institute of Bioanalysis, Medical School, University of Pécs, Szigeti út 12., H-7624 Pécs, Hungary

**Keywords:** lutein, *cis*-isomers, analysis, isomerization, HPLC-DAD-MS, NMR, petals

## Abstract

Lutein and its *cis*-isomers occur in a lot of plants, including a variety of flowers. In this study, lutein isomers were produced via iodine-catalyzed isomerization, and four *cis*-isomers (9*Z*-, 9′*Z*-, 13*Z*-, and 13*Z*′) were isolated by means of column chromatography and semipreparative HPLC. The structures of the 9′*Z*- and 13′*Z*-isomers were elucidated via NMR measurements. These compounds were used as standards for the HPLC-DAD-MS determination of the carotenoid composition of the flowers of 20 plant species, in which lutein and its geometrical isomers are the main components. The flowers showed great variation in their *cis*- and *trans*-lutein content, and also in the presence or absence of other carotenoids, such as violaxanthin, neoxanthin, β-cryptoxanthin, and β-carotene. Some of the investigated flowers were found to be rich sources of lutein without zeaxanthin.

## 1. Introduction

**Figure 1:**
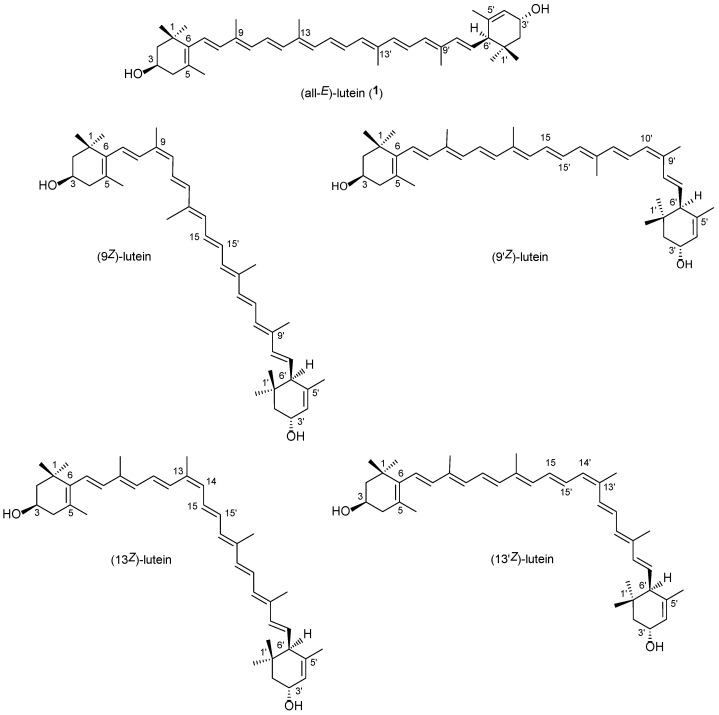
The main geometrical isomers of lutein.

Lutein ((all-*E*,3*R*,3′*R*,6′*R*)-β,ε-carotene-3,3′-diol) (**1**, Figure 1) can be found in all plant leaves as an important part of the light-harvesting complex, taking part in the energy transfer from light to the photosynthetic machinery [1]. As with most hydroxy-carotenoids, it occurs there as a fatty acid ester. It can also be found in several vegetables and fruits, so human consumption is considerable as well. Lutein accumulates in the macula. In human blood, not only lutein but also a lot of its isomers and derivatives can be detected, such as 3′-epilutein (**2**) (Figure S1), *cis*-isomers, anhydrolutein II and I, and 3′-oxo-lutein [2]. It has a plethora of biological functions, including the reduction of age-related macular degeneration in the eye [3]. It can also pass through the blood–brain barrier, has a neuroprotective effect, and reduces the risk of dementia and related diseases [4,5,6].

Lutein was first isolated from green leaves in 1907 [7], although the absolute configuration was only determined in 2004 by means of X-ray measurements [8]. It has stereoisomers due to the three chiral centers: carbons 3, 3′, and 6′. The 3′ hydroxyl group is allylic, unlike in its regioisomer, zeaxanthin (**3**) (Figure S1). Some stereoisomers can be found in nature, such as 3′-epilutein (**2**) with an (all-*E*,3*R*,3′*S*,6′*R*) configuration, which has been isolated from goldfish (*Carassius auratus*) [9], marsh marigold (*Caltha palustris*) [10,11,12], and other flowers [13]. Moreover, 3′-epilutein (**2**) forms from lutein (**1**) under acid-catalyzed epimerization in processed plants [14,15]. This epimerization can also be triggered in the laboratory [12,16]. Other minor isomers, such as 6′-epilutein (lutein F, (all-*E*,3*R*,3′*R*,6′*S*)-β,ε-carotene-3,3′-diol) and 3′,6′-diepilutein (lutein D, (all-*E*,3*R*,3′*S*,6′*S*)-β,ε-carotene-3,3′-diol), can be isolated from fish [17,18].

It is generally considered that most carotenoids occur in nature as all-*E* isomers. However, natural *Z*-isomers can also be isolated. For example, Khachik et al. obtained (13*Z*,13′*Z*)-lutein from kale and marigold flowers [19].

All carotenoid isomers undergo reversible geometrical isomerization in a solution. The main mono-*Z*-isomers (9*Z,* 9′*Z*, 13*Z*, and 13′*Z*) of both lutein (**1**) and 3′-epilutein (**2**), as well as a di-*Z*-isomer (9*Z*,9′*Z*) of lutein, were prepared via the thermal and the I_2_-catalyzed photoisomerization of their solutions [20,21,22]. In 1981, our research group isolated and identified these isomers via open column chromatography on calcium carbonate, and ^13^C-NMR studies confirmed the existence of four mono-*cis* isomers at positions 9, 9′, l3, and 13′, respectively. In 2001, geometrical isomers of lutein (**1**) and zeaxanthin (**3**) were separated using hyphenated HPLC-MS and HPLC-NMR systems on a C_30_ column [23]. The established elution order was (13*Z*)-, (13′*Z*)-, (all-*E*), (9*Z*)-, and (9′*Z*)-isomer. Some years later, Aman et al. published a similar separation method via LC-NMR [24].

Only a few papers have dealt with the bioavailability and antioxidant properties of the *cis*-isomers of lutein. The bioaccessibility proved to be higher for the *cis-* than the *trans*-isomer. The antioxidant activities were also better in the case of the *Z*-isomers, although it is too early to draw any conclusions in this regard due to the lack of data [25].

In flower petals, although there is high diversity, some carotenoids, such as lutein (**1**), β-cryptoxanthin (**4**) (Figure S1), and zeaxanthin (**3**), almost always occur. These carotenoids and epoxy carotenoids, such as violaxanthin (**5**), antheraxanthin (**6**), neoxanthin (**7**), and lutein-5,6-epoxide (**8**) (Figure S1), are responsible for the characteristic yellow color of many petals. In general, the carotenoid composition of petals is different from that of leaves. The flower petals of most plants accumulate both β,β- and β,ε-carotenoids. In some cases, the petals contain either β,β-carotenoids (*Sandersonia aurantiaca*, *Camellia chrysantha, Ipomoea* sp., etc.*)* [26,27,28] or β,ε-carotenoids (*Tagetes* sp., *Chrysanthemum morifolium,* etc.) [29,30], but not both. Some, although not too many, *cis*-carotenoids can also be detected in these petals, including di-, tri-, and even tetra-*cis* derivatives [30,31,32]. Di-*cis*-carotenoids are also found in the petals of *Viola tricolor* [33] and *Solidago canadensis* [34].

Here, we describe the identification and occurrence of lutein and its *cis*-isomers in some medicinal plants. In addition, we provide the carotenoid composition of the flowers of the investigated plants.

## 2. Results

### 2.1. Identification of Lutein Isomers

**Figure 2:**
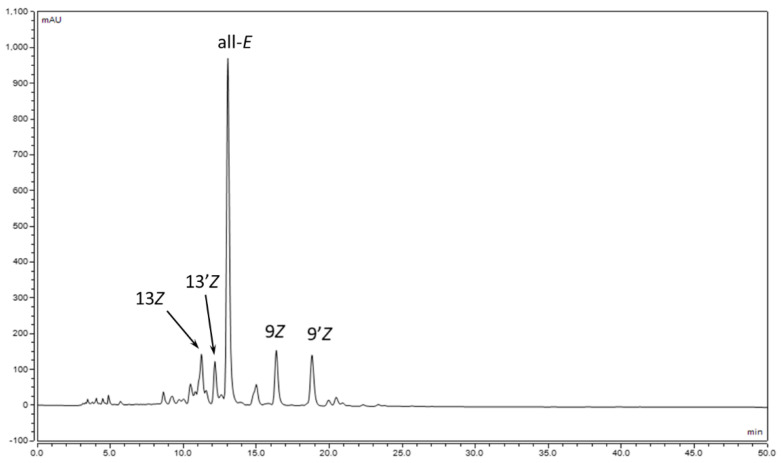
Separation of lutein isomers on a C_30_ phase.

The geometrical isomers (9*Z*)-, (9′*Z*)-, (13*Z*)-, and (13′*Z*)-luteins were previously separated by means of HPLC, although due to the lack of standards, we were not able to identify unambiguously the peaks in the chromatogram [35]. To prepare such standards, the iodine-catalyzed isomerization of (all-E)-lutein (**1**) (50 mg) was performed in toluene solution. The isomerized mixture was submitted to open column chromatography (CaCO_3_, hexane-toluene 60:40), which resulted in three main zones: zone 1: a mixture of (13Z)-and (13′*Z*)-lutein; zone 2: a mixture of (9*Z*)- and (9′*Z*)-lutein; and zone 3: (all-E)-lutein. The separations of the (13*Z*), (13′*Z*) and (9*Z*), (9′*Z*) isomers were achieved via semipreparative HPLC on a C_30_ column. Unfortunately, in both cases, only one isomer, the one with the higher retention time, was obtained in a pure form. Based on the NMR analysis, however, the isomers could be clearly identified (Figure 2, Table 1, Figure S2).

#### 2.1.1. (9′*Z*)-Lutein

The low intensity of the cis peak in the UV-visible spectrum (λ_max_: 330, 440, 467 nm, Q: 13.56, A_B_/A_II_%: 8.5) indicates a (9*Z*) or (9′*Z*) isomer. The exact position of the *Z*-double bond can primarily be established from the ^13^C-NMR spectrum. The chemical shifts of the C-8 or C-8′ are characteristic of Z-isomers. In the (all-E) compound, the C-8 (δ = 138.5 ppm) and C-8′ (δ = 137.7 ppm) can be distinguished unambiguously. In the *Z*-isomers, the δ for the C-8 or C-8′ shows an upfield shift of ca. 7–8 ppm, depending on the position of the Z double bond, while the other signal remains unchanged [20]. Here, the C-8′ has a chemical shift of 129.9 ppm, clearly showing the (9′*Z*)-isomer.

Less obvious but also visible from the ^1^H-NMR spectra: by comparing the chemical shifts (δ) of the H-6′ in the spectrum of the (all-*E*)-lutein and that of its (9*Z*)- or (9′*Z*)-isomers, in the (all-*E*) compound, the δ value of the H-6′ is 2.40 ppm, while in the (9′*Z*)-isomer, it is 2.47 ppm (doublet, *J* = 10.0 Hz). (In the 9*Z*-isomer, the δ of the H-6′ is the same as in the (all-*E*) compound). A similarly tiny increase in the chemical shift of the CH_3_-18′ can also be observed, where the δ is 1.62 ppm for the (all-*E*)-isomer and 1.65 ppm for the (9′*Z*)-isomer. These data are in accordance with the literature [36]. The chemical shifts of the H-10′ and H-8′ in the (9′*Z*)-lutein change dramatically compared to the (all-*E*) compound; however, they are not suitable for the distinction of the (9*Z*)- and (9′*Z*)-isomers. In the (all-*E*) compound, the chemical shift of the H-10 and that of the H-10′ are almost identical and overlap with other signals. Moreover, in both *Z*-isomers, depending on the position of the *Z* double bond, the H-10 and H-10′ show very similar δ values. The chemical shift of the H-8 or H-8′ increases by ca. 0.5 ppm in the *Z*-isomers, although this signal overlaps with others in the spectra of all the isomers [22]. 

#### 2.1.2. (13′Z)-Lutein

The UV-vis spectrum suggests a *Z*-configured double bond in position 13 or 13′ (λ_max_: 331, 438, 464 nm, Q: 2.37, A_B_/A_II_%: 41.7). Both the ^1^H- and ^13^C-NMR spectra support this finding. The chemical shift of the H-12′ is downfielded by 0.5 ppm compared to the (all-*E*)-isomer, and the H-15′ also has a 0.1 ppm higher chemical shift in this compound. However, these characteristic differences are not suitable for distinguishing the (13*Z*)- and the (13′*Z*)-isomers, because the H-12 and 12′ (6.36 and 6.35 ppm), as well as the H-15 and H-15′ (6.67–6.58 ppm), have very similar chemical shifts, respectively, in the (all-*E*)-lutein. Similarly, the upfield change in the H-14′ by 0.1 ppm in the *Z*-isomer does not help, as the H-14 and H-14′ give the same chemical shift in the (all-*E*)-compound. The ^13^C spectra also show some significant differences between the (all-*E*)- and the (13/13′*Z*)-isomers. Dramatic upfields of the C-12/12′ and C-14/14′, as well as downfields of the C-11/11′ and C-20/20′, can be detected. As these pairs of signals cannot be distinguished in the spectrum of the (all-*E*)-isomer, the changes do not inform us about the exact position of the *Z* double bond.

To distinguish the (13*Z*)- and (13′*Z*)-isomers via simple 1D NMR measurements, the very tiny differences in the chemical shifts of the C-7 and C-7′ or C-9 and C-9′ can be used. In the (13′*Z*)-lutein, the C-7′ has a downfield of 0.4 ppm compared to the all-*E* compound, while the C-9′ shows a downfield of 0.7 ppm.

The unambiguous evidence concerning the 13′*Z* structure requires a ^13^C-^1^H-HMBC experiment, which allows us to distinguish the H-12 and H-12′. A further ^13^C-^1^H-HSQC measurement helps in the identification of the C-12 and C-12′ as well as the C-20 and C-20′ (Figure S2). (Table 1)

### 2.2. Analysis of Flowers

In this study, the flowers of 20 species, which belong to eight families (Appendix A), were used. Whenever it was possible, the petals were investigated, although in most cases, due to the size of the flower, we could not separate the stamens and petals, so we analyzed the entire inflorescence. The total carotenoid contents were determined via UV-Vis spectrophotometry [37] and typically varied between 0.2 and 0.5 mg/g fresh material. The *Glottiphyllum cruciatum* and *Echinacea paradoxa* contained less than 0.1 mg/g, while the *Rorippa austrica* contained almost 1 mg/g of carotenoids.

The HPLC-DAD and HPLC-DAD-MS analyses were performed on a C_30_ phase. Our previous results [35] showed that this stationary phase separates the isomers of interest well, while a C_18_ phase is less suitable. The carotenoids were identified by their elution order on the C_30_ HPLC column, spiking with the authentic standards, UV-visible spectra (λ_max_, spectral fine structure (% III/II)), *cis* peak intensity (% A_B_/A_II_), and mass spectrum compared to the standards and data available in the literature [36].

**Figure 3:**
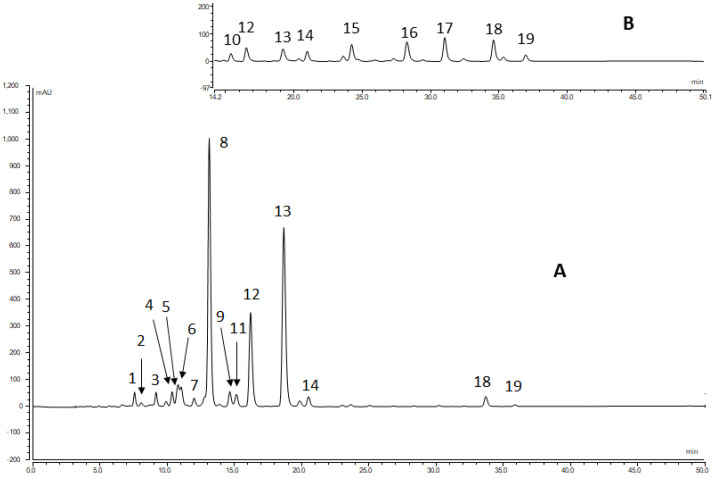
HPLC-DAD full chromatogram of *Anthemis tinctoria* (**A**) and a second part of *Coreopsis pubescens* (**B**). Peak numbering is given in Table 2.

In most of the investigated flowers, 16–19 carotenoids were detected in the total extract via HPLC on a C_30_ column at a 450 nm wavelength (Table 2). The identification of the components is shown in the example of *Anthemis tinctoria* and *Coreopsis pubescens* (Figure 3). 

The main component, Peak 8, was identified as (all-*trans*)-lutein (**1**) via its UV-VIS (444, 472 nm) and MS spectra (*m/z* 551, [M-H_2_O + H]^+^) and co-chromatography with an authentic sample. Peaks 12 and 13 were attributed to the 9-*cis* and 9′-*cis* isomers of lutein, respectively, according to the characteristic hypsochromic shift and the low intensity of the *cis* peak in the UV-visible spectra (330, 440, 467 nm, Figure S3), the MS ([M-H_2_O + H]^+^ at *m/z* 551), and spiking with the iodine-catalyzed isomerization of lutein. In the case of the *Anthemis tinctoria* (Figure S4.1), *Colchicum autumnale* (Figure S4.2), and *Helicrysum italicum* (Figure S4.3), another peak was found, which could be a lutein isomer. The *m/z* value of Peak 9 was 551, and its absorption maximum occurred at a wavelength 2–3 nm lower than that of the 9*Z*-isomer. The intensity of the *cis* peak was low. This suggests that this component is the (9*Z*,9′*Z*)-isomer of lutein. Moreover, (9*Z*,9′*Z*)-lutein was previously isolated from *Solidago gigantea* [34] as one of the main components. Unfortunately, we did not have an authentic sample for full identification.

Peaks 6 and 7 proved to be the (13*Z*)- and (13′*Z*)-isomers of lutein, respectively, according to the characteristic hypsochromic shift of the λ_max_ and the high intensity of the *cis* peak in the UV-visible spectra (Figure S3). The *m*/*z* value of 551 in its mass spectrum, as well as the co-chromatography with the iodine-catalyzed isomerization mixture of lutein (**1**), also supported the identification of (13*Z*)- and (13′*Z*)-lutein. In some cases, the (13*Z*)-isomer of lutein (peak 6) was partially covered by Peak 5, and it could be identified via EIC spectra at *m*/*z* 551. 

The (all-*trans*)-Violaxanthin (**6**) (Peak 2) and (9*Z*)-violaxanthin (Peak 5) showed characteristic UV-visible spectra, with a slightly decreased spectral fine structure and a hypsochromic shift of 4 nm for the Peak 5 compound compared to the all-*trans* isomer. The identification of these compounds was supported by co-elution with authentic standards and by their *m/z* value ([M + H]^+^ = 601).

From the polar carotenoids, the (all-*trans*)-neoxanthin (**7**) (Peak 1) showed characteristic UV-visible spectra (λ_max_ 414, 438, 468 nm) with a fine structure. The identity of Peak 1 was supported by co-elution with an authentic standard and by its *m*/*z* value (600). Peak 3 was identified similarly to Peak 5, that is, as (9′*Z*)-neoxanthin (**9**). In most plants, lutein is usually accompanied by small amounts of zeaxanthin (**4**). Peak 10 was identified as zeaxanthin via its UV-vis and MS spectrum. It should be noted that we could not detect zeaxanthin in any of the flowers. An unidentified component (Peak 11) with a different UV-vis spectrum and molar mass was found, with a very similar retention time. 

Plants containing lutein almost always contain small amounts of β-carotene. Peak 18 had UV-visible spectra similar to those of zeaxanthin. The molecular mass detected at 536 seemed to correspond to (all-*trans*)-β-carotene (**10**). Peak 19 was the 9*Z*-isomer and Peak 16 was the 13*Z*-isomer of β-carotene, based on their UV-VIS and MS spectra. This assumption was confirmed by spiking with the authentic standards. Peak 17, with a spectrum similar to lutein and an [M + H]^+^ value of 537, was identified as α-carotene (**11**).

Peaks 13 and 14 gave UV-visible spectra similar to those of Peaks 17 and 18. The molecular masses ([M + H]^+^), as detected at *m*/*z* 553 for both compounds, seemed to correspond to (all-*E*)-α-cryptoxanthin (**12**) and (all-*E*)-β-cryptoxanthin (**3**).

The chromatograms of the *Helianthus angustifolius* (Figure S4.4), *Helianthus tuberosus* (Figure S4.5), and *Sternbergia lutea* (Figure S4.6) contained a peak at 10.75 min, which had an UV-VIS spectrum similar to that of lutein, and the *m*/*z* value was 585. Based on these data and co-chromatography with an authentic sample, the peak was identified as antheraxanthin (**5**). 

In the *Coreopsis pubescens* (Figure S4.7)*,* we found a relatively large peak at 28.3 min. Based on the spectrum being similar to lutein, the *m*/*z* 553 molecular weight, and the retention time, the compound was identified as β-carotene 5,6-epoxide (**13**). Only two peaks, Peaks 4 and 11, remained unidentified, meaning they are presumably mixed peaks.

## 3. Discussion

The geometrical isomers of lutein show characteristic UV-vis spectra from which the position of the *Z* double bond can be established. However, lutein is a non-symmetrical β,ε-carotenoid (Figure 1), which results in different 9*Z* or 9′*Z* and 13*Z* or 13′*Z*-isomers. In our previous studies [35], the lutein isomers were separated, although due to the lack of standards, we could not distinguish between the (9*Z*) and (9′*Z*) or the (13*Z*) and (13′*Z*) isomers.

In this study, (9′*Z*)- and (13′*Z*)-lutein were isolated in a pure form from the iodine-catalyzed photoisomerization mixture of lutein using classical column chromatography and semipreparative HPLC. The structure of the pure products was confirmed via NMR studies. With the two known structures in mind, we were able to determine the elution order of the lutein isomers on the C_30_ stationary phase, which was 13*Z*, 13′*Z,* all-*E,* 9*Z*, and 9′*Z,* respectively. The elution order was the same as that previously described by Dachtler [21] and Aman [22], which shows that the elution order depends on the quality of the stationary phase and does not depend on the eluent composition and gradient program.

The other part of this work focused on the identification of carotenoids in 20 fresh flowers by means of HPLC. The investigated flowers could be classified into eight different families: Acanthaceae, Aizoaceae, Amaryllidaceae, Asteraceae, Brassicaceae, Colchicaceae, Euphorbiaceae, Fabaceae, and Nymphaceae (Appendix A). The colors varied from pale yellow to dark yellow. Lutein and its geometrical isomers were the major carotenoids in all the flowers. The amount of (all-*E*)-lutein varied between 26% and 76%. In addition to the low (all-*E*)-lutein content, we generally measured relatively high amounts of (9*Z*)- or (9′*Z*)-lutein. The proportion of the (9*Z*)- and (9′*Z*)-lutein isomers was almost the same, except for the *Anthemis tinctoria* and *Helichrysum italicum*, where the amount of the (9′*Z*)-isomer reached 30%, while the (9*Z*)-isomer in the case of the *Anthemis tinctoria* was approx. half or a third. This ratio was observed for the *Anthemis tinctoria* collected from different locations and at different times. An even greater difference was found in the case of the *Helicrysum italicum*, where in addition to the nearly 30% (9′*Z*)-isomer, only approx. 3% (9*Z*)-isomer was detected.

In the case of the (13*Z*)- and (13′*Z*)-lutein isomers, no such differences were found. The determination of the proportions was made difficult by the fact that in many cases, the peak of the (13*Z*)-lutein overlapped with the peak of the (9*Z*)-violaxanthin. In the *Anthemis tinctoria*, *Colchicum autumnale*, and *Helichrysum italicum,* we found a di-*cis*-isomer of lutein, namely (9*Z*,9′*Z*)-lutein, which was earlier identified as the main carotenoid in the *Solidago gigantea* [34].

Zeaxanthin almost always occurs alongside lutein in plants. To the best of our knowledge, no source has already been described from which lutein without zeaxanthin can be isolated. During our investigations, we could not detect even traces of zeaxanthin in the extracts of *Anthemis tinctoria*, *Erysimum cheiri*, *Colchicum autumnale,* and *Rorippa austriaca*.

The *Cassia artemisioides* contained a surprisingly large amount of α-cryptoxanthin (15%) (Figure S4.8), while no trace of β-cryptoxanthin was found. This is interesting because β-carotene also occurred in a significant amount (27%) in the *Cassia artemisioides*. Moreover, α-cryptoxanthin and β-carotene were also found in the other examined plants, typically in amounts between 0.3% and 3.5% and 0.2% and 7.5%, respectively. By contrast, only the *Coreopsis pubescens* and *Helichrysum italicum* contained β-cryptoxanthin in approximately 4%. In the other plants, it was significantly smaller than this or it was not detectable.

Furthermore, α-carotene occured in higher amounts only in the *Echinacea paradoxa* (17%) (Figure S4.9), while the *Erysimum cheiri*, *Helianthus angustifolius*, *Coreopsis pubescens, Cassia artehmisioides*, *Pachystachys lutea*, *Nuphar lutea,* and *Euphorbia palustris* contained 2–7%. The other flowers contained only traces of α-carotene. These results indicate that the carotenoid composition is a mixture of carotenoids containing the β,β- and β,ε-end groups.

The Echinacea *paradoxa* and *Euphorbia polychroma* did not contain epoxy carotenoids at all, while the *Glottiphyllum cruciatum* contained only trace amounts. The *Cassia artemisioides* and *Nuphar lutea* contained only (all-*E*)-neoxanthin and its 9′*Z*-isomer, while the *Coreopsis pubescens* and *Helianthus tuberosus* contained only (9′*Z*)-neoxanthin. The *Coreopsis verticillata* contained (all-*E*)-neoxanthin, while the *Pachystachys lutea* contained a larger amount (9%) of (9′*Z*)-neoxanthin as well as (all-*E*)- and (9*Z*)-violaxanthin. Additionally, (all-*E*)- and (9′*Z*)-neoxanthin, as well as (all-*E*)- and (9*Z*)-violaxanthin, were also found in smaller amounts in the other extracts. The *Helianthus tuberosus* contained 10% and the *Helianthus angustifolius* contained 2% antheraxanthin, while none of it was found in the other flowers.

β-carotene 5,6-epoxide was identified only in the extract of one flower, *Coreopsis pubescens*, and we could not detect even a trace of it in the others. Although the main component in all the investigated samples was lutein or its isomers, we could not detect lutein 5,6-epoxide in any of them.

Summarizing our results, we can conclude that flowers belonging to different families may have a similar carotenoid composition, which in our case is a mixture of carotenoids containing the β,β- and β,ε-end groups. The found carotenoids are known to be biosynthesized by the subsequent cyclisation, hydroxylation, and epoxydation of lycopene [38]. The low content of α-carotene and α-cryptoxanthin indicates that the hydroxylation of not only the β- but also the ε-end group takes place quickly, and lutein is produced. The absence of the β,β-end group hydroxy-carotenoids, especially β-cryptoxanthin and zeaxanthin, shows that epoxidation is faster compared to hydroxylation. However, lutein is apparently not a good substrate for the epoxidase enzyme, as no lutein 5,6-epoxide was found in any of the inflorescences. 

## 4. Materials and Methods

### 4.1. Plant Materials 

The plant material was collected from natural habitats in Hungary and from botanical gardens in Pécs (Melius Botanical Garden), Bratislava, and Brno (Table 2). Where possible, we examined the petals, although in some cases, due to the small size of the flower, we could not separate the stamens and petals, so we analyzed the entire flowers or inflorescences. The green calyx leaves and other green parts were carefully excluded from the flowers to avoid their lutein content falsifying the measurements. The plant material was collected and identified by botanists, and voucher examples were deposited in the Institute of Pharmacognosy, University of Pécs.

### 4.2. Pigment Extraction and Determination of Carotenoid Content

Analytical-grade chemicals were used for the extractions. Extraction: Flower samples were extracted twice with acetone and once with Et_2_O. After evaporation, the residue of the acetonic extracts was dissolved in Et_2_O. The ethereal solutions were combined and the total extract was saponified in a heterogeneous phase (30% KOH/MeOH) overnight. The reaction mixture was washed with water 10 times. The saponified pigments were stored in benzene at −20 °C under nitrogen. 

The total carotenoid content of the plant materials was determined photometrically [37]. The UV-VIS spectra were recorded with a Jasco V-530 spectrophotometer (Jasco Corporation, Tokyo, Japan). The NMR spectra were recorded with a Bruker Avance III Ascend 500 spectrometer (500/125 MHz for ^1^H/^13^C) in CDCl_3_. The chemical shifts are referenced to Me_4_Si (^1^H) or the residual solvent signals (^13^C).

#### 4.2.1. High-Performance Liquid Chromatography

The solvents used for the analysis (MeOH: methanol, MTBE: tert-butylmethyl ether, water) were of HPLC grade.

The HPLC-DAD separations on a C_30_ stationary phase were performed using a Dionex 3000 HPLC system (Thermo Fisher Scientific Inc., Waltham, MA, USA). The chromatograms were detected at a 450 nm wavelength, and the data acquisition was performed using Chromeleon 7.20 software. The separation was carried out on an endcapped C_30_ column (250 × 4.6 mm i.d.; YMC C_30_, 3 µm, YMC Europe GmbH, Dinslaken, Germany). Eluents: (A) MeOH:MTBE:H_2_O = 81:15:4 *v*/*v*%; and (B) MeOH:MTBE:H_2_O = 6:90:4 *v*/*v*%. The chromatography was performed in a linear gradient from 100% A eluent to 50% B mixture in 45 min, with a 1.00 mL/min flow rate at 22 °C.

The HPLC-DAD-MS chromatograms were recorded using an 6530 Accurate-Mass Q-TOF LC/MS apparatus (Agilent Technologies, Yishun, Singapore), and the data acquisition was performed using MassHunter B.04.00 software. For the LC–(APCI)MS, the positive ion mode was used, with TIC, a scanning range of 200–1500 *m*/*z*, a corona voltage of 2.6 kV, a fragmentor voltage of 150 V, a skimmer of 60 V, and an Oct 1RF Vpp of 750 V. The flow rate of the dried nitrogen used as a nebulizer gas was 240 L/h, and the vaporizer temperature was 400 °C. The HPLC conditions were the same as described above.

The pure (9′*Z*)- and (13′*Z*)-lutein were obtained via semipreparative HPLC of the corresponding fractions from the open column chromatography on CaCO_3_, (hexane-toluene 60:40). Equipment for the semipreparative HPLC-DAD: Column: 250 × 10.0−mm YMC C_30_; Eluents: (A) MeOH:MTBE:H_2_O = 81:15:4 *v*/*v*% and (B) MeOH:MTBE:H_2_O = 6:90:4 *v*/*v*%. The chromatograms were performed in a linear gradient from 100% A to 25% B eluent in 25 min at 22 °C. Flow rate: 3.00 mL/min, detection at 450 nm.

#### 4.2.2. Identification of the Peaks 

The carotenoids were identified using the following data: elution order on the C_30_ HPLC column, spiking with authentic standards, UV-visible spectrum (λ_max_, spectral fine structure (% III/II)), *cis* peak intensity (%A_B_/A_II_), and mass spectrum (molecular ion and fragments) compared to the standards and data available in the literature [35].

The authentic samples were taken from our collection.

### 4.3. Isomerization of Lutein

The lutein was isolated in the same way as in our previous article [4]. In brief, diethyl ether was used to dissolve the marigold extract (INEXA C.A., Quito, Ecuador), and it was saponified overnight with 30% KOH in methanol. Next, the ethereal solution was washed five times with water, and thereafter, it was dried and evaporated. The crude saponified extract was crystallized from hexane/toluene to deliver lutein with 98% purity. According to the HPLC on a C_30_ column, the lutein contained 6% zeaxanthin. 

A solution of 50 mg of lutein in 500 mL of toluene was isomerized under N_2_ in scattered daylight in the presence of 1 mg of I_2_ (~2% to the carotenoid) [39,40]. The isomerization was monitored by means of UV/VIS, and when the thermodynamic equilibrium was reached (ca. 40 min), the mixture was washed free of I_2_ with 5% Na_2_S_2_O_3_ soln. After the usual workup, the mixture was submitted to open column chromatography (CaCO_3_, hexane-toluene 60:40). The separation of the isomerization mixture of lutein resulted in the following picture after development: 4 mm of pale yellow (unidentified), 3 mm of intermediate zone, 15 mm of yellow (*Zone* 1; (13*Z*)- and (13′*Z*)-lutein), 4 mm of intermediate zone, 20 mm of pale yellow (*Zone* 2; (9*Z*)- and (9′*Z*)-lutein), 50 mm of intermediate zone, 8 mm of pale yellow (unidentified), 20 mm of intermediate zone, and 40 mm of yellow (*Zone* 3; (*all*-*E*)-lutein).

## 5. Conclusions

In conclusion, lutein and its mono-*cis* isomers were found to be the main carotenoids in the studied flowers. The (9*Z*)-, (9′*Z*)- and (13*Z*)-, (13′*Z*) isomers of lutein accumulated in approximately equal proportions in all but two of the examined flowers. The *Anthemis tinctoria* and *Helichrysum italicum* contained ca. 30% of (9′*Z*)-lutein, which was probably produced enzymatically. With the exception of the above two flowers and the *Rorippa austriaca*, the proportion of (9*Z*)-lutein was higher compared to (9′*Z*)-lutein. In four of the examined flowers, we could not detect zeaxanthin; thus, these plants could be sources of zeaxanthin-free lutein. It was also established that flowers belonging to different families may have similar carotenoid composition, and probably, that members of the same family can have different carotenoid composition. Our future research will be undertaken to improve our understanding of the wide chemical diversity of flowers’ carotenoids. 

## Figures and Tables

**Table 1:** ^1^H- and ^13^C-NMR data of (all-*E*)-, (9′*Z*)-, and (13′*Z*)-lutein at 500/125 MHz, respectively, in CDCl_3_ at room temperature. All ^1^H and ^13^C spectra were assigned with the help of ^1^H-^1^H-COSY, ^13^C-^1^H-HSQC, and ^13^C-^1^H-HMBC experiments.

	(all-*E*)-Lutein			(9′*Z*)-Lutein			(13′*Z*)-Lutein		
Position	δ(H) [ppm]	J [Hz]	δ(C) [ppm]	δ(H) [ppm]	J [Hz]	δ(C) [ppm]	δ(H) [ppm]	J [Hz]	δ(C) [ppm]
1	-	-	37.1	-	-	37.1	-	-	37.1
2	α: 1.77 (*dt*)	12.1, 3.1	48.5	α: 1.79–1.75 (*m*)	-	48.5	α: 1.77 (*pd*)	12.9	48.5
	β: 1.48 (*t*)	11.9		β: 1.48 (*t*)	11.9		β: 1.48 (*t*)	11.8	
3	4.03–3.97 (*m*)	-	65.1	4.03–3.97 (*m*)	-	65.1	4.06–3.96 (*m*)	-	65.1
4	α: 2.39 (*dd*)	15.7, 5.1	42.6	α: 2.39 (*dd*)	16.4, 4.8	42.6	α: 2.40 (*dd*)	5.8	42.6
	β: 2.04 (*dd*)	16.6, 9.7		β: 2.05 (*dd*)	16.9, 9.7		β: 2.05 (*dd*)	16.5, 9.4	
5	-	-	126.2	-	-	126.2	-	-	126.1
6	-	-	137.8	-	-	137.8	-	-	137.8 ^j^
7	6.09 (*d*) *	16.7	125.6	6.10 (*d*) *	16.9	125.6	6.09 (*d*) *	16.6	125.5
8	6.10 (*m*) *	-	138.5	6.12 (*brs*) *	-	138.5	6.12 (*brs*) *	-	138.5
9	-	-	135.7	-	-	135.7	-	-	135.6 ^k^
10	6.15 (*d*) *	10.5	131.3	6.16 (*d*) *	11.4	131.3	6.10–6.17 (*m*) *	-	131.3
11	6.67–6.58 (*m*)	-	124.9 ^b^	6.77–6.71 (*m*) ^f^	-	124.9^g^	6.66–6.53 (*m*)	-	124.8
12	6.36 (*d*) ^a^	14.9	137.57 ^c^	6.36 (*d*)	14.9	137.6	6.38 (*d*)	15.0	137.6
13	-	-	136.5 ^d^	-	-	136.4 ^h^	-	-	136.3
14	6.25 (*d*)	9.5	132.6	6.25 (*pd*) *	-	132.6	6.24 (*d*)	11.6	132.4
15	6.67–6.58 (*m*)	-	130.09 ^e^	6.68–6.62 (*m*)	-	130.0 ^i^	6.66–6.53 (*m*)	-	129.2
16	1.07 (*s*)	-	30.3	1.07 (*s*)	-	30.3	1.07 (*s*)	-	30.3
17	1.07 (*s*)	-	28.7	1.07 (*s*)	-	28.8	1.07 (*s*)	-	28.7
18	1.73 (*s*)	-	21.6	1.74 (*s*)	-	21.6	1.74 (*s*)	-	21.6
19	1.97 (*s*)	-	12.81 ^l^	1.973 (*s*) ^m^	-	12.9 ^n^	1.97 (*s*)	-	12.7
20	1.97 (*s*)	-	12.75 ^l^	1.968 (*s*) ^m^	-	12.82 ^n^	1.98 (*s*)	-	12.7
1′	-	-	34.0	-	-	34.0	-	-	34.0
2′	α: 1.84 (*dd*)	13.2, 5.9	44.6	α: 1.86 (*dd*)	13.2, 5.9	44.8	α: 1.85 (*dd*)	13.2, 6.0	44.6
	β: 1.36 (*dd*)	13.1, 6.8		β: 1.38 (*dd*)	13.0, 6.9		β: 1.37 (*dd*)	13.0, 6.8	
3′	4.25 (*brs*)	-	65.1	4.27 (*brs*)	-	65.9	4.25 (*brs*)		65.9
4′	5.54 (*s*)	-	124.5	5.56 (*s*)	-	123.6	5.55 (*s*)	-	124.5
5′	-	-	138.0	-	-	137.8	-	-	137.9 ^j^
6′	2.40 (*d*)	14.5	55.0	2.47 (*d*)	10.0	55.3	2.42 (*d*)	9.2	55.0
7′	5.43 (*dd*)	15.4, 9.9	128.7	5.46 (*dd*)	15.3, 10.0	130.9	5.45 (*dd*)	15.3, 10.0	129.1
8′	6.13 (*d*) *	16.7	137.7	6.68–6.62 (*m*) *	-	130.1	6.10–6.17 (*m*)	-	137.7
9′	-	-	135.1	-	-	133.7	-	-	135.8 ^k^
10′	6.10 (*m*) *	-	130.8	6.02 (*d*)	11.3	129.3	6.17 (*d*) *	~13.0	130.9
11′	6.67–6.58 (*m*)	-	124.8 ^b^	6.77–6.71 (*m*) ^f^	-	124.6 ^g^	6.66–6.53 (*m*)	-	126.1
12′	6.35 (*d*) ^a^	14.9	137.56 ^c^	6.29 (*d*)	14.9	136.9	6.88 (*d*)	14.7	129.4
13′	-	-	136.4 ^d^	-	-	136.3 ^h^	-	-	134.8
14′	6.25 (*d*)	9.5	132.6	6.25 (*pd*) *	-	132.5	6.10–6.17 (*m*) *	-	130.8
15′	6.67–6.58 (*m*)	-	130.04^e^	6.68–6.62 (*m*)	-	129.9 ^i^	6.79 (*pt*)	12.5	128.7
16′	0.85 (*s*)	-	24.3	0.86 (*s*)	-	24.3	0.85 (*s*)	-	24.3
17′	1.00 (*s*)	-	29.5	1.03 (*s*)	-	29.5	1.00 (*s*)	-	29.5
18′	1.62 (*s*)	-	22.28	1.65 (*s*)	-	22.9	1.63 (*s*)	-	22.8
19′	1.91 (*s*)	-	13.1	1.91 (*s*)	-	21.1	1.92 (*s*)	-	13.1
20′	1.97 (*s*)	-	12.81 ^l^	1.99 (*s*) ^m^	-	12.75 ^n^	1.97 (*s*)	-	20.7

^a–n^ Assignment may be exchanged; * overlapped with other signals.

**Table 2:** Carotenoid composition of the investigated flowers via HPLC analysis. Color shades show the same family.

Peak No.	Carotenoid	Retention Time (min)	UV-Vis λ_max_ (nm)	Plant Source: MS (*m/z*)	Golden Marguerite (*Anthemis tinctoria*)				Immortelle (*Helichrysum italicum)*	Jerusalem Artichoke (*Helianthus tuberosus)*	Narrowleaf Sunflower (*Helianthus angustiflius)*	Star Tickseed *(Coreopsis pubescens)*	Whorled Tickseed *(Coreopsis verticillata)*	Yellow Coneflower *(Echinacea paradoxa)*	Water Lily (*Nuphar lutea*)	Golden Shrimp (*Pachystachys lutea*)
1	Neoxanthin	6.77	416, 440, 468	601 [M + H]^+^	1.68	1.34	0.48	0.44	0	0	0.23	0	1.37	0	1.57	tr
2	Violaxanthin	7.49	416, 440, 468	601 [M + H]^+^	2.98	2.00	1.61	2.18	0.50	9.35	1.97	1.29	0	0	9.76	9.12
3	(9′*Z*)-Neoxanthin	8.35	411, 434, 463	601 [M + H]^+^	0.70	5.13	0.65	0.46	2.21	0	0.41	0	3.39	0	tr	1.20
4	Unidentified mixture	9.16			1.62	1.34	2.14	5.01	3.69	10.70 *	2.36 *	1.45	0.90	0	5.25	0
5	(9*Z*)-Violaxanthin	11.01	411, 434, 463	601 [M + H]^+^	3.51	2.43	2.44	1.20	7.62	0	0	0	3.34	0	5.08	2.11
6	(13*Z*)-Lutein	11.87	331, 435, 463	551 [M-H_2_O + H]^+^	3.82	2.42	3.93	5.56	0	2.03	3.86	1.57	3.79	1.34	0	2.89
7	(13′*Z*)-Lutein	12.35	331, 438, 464	551 [M-H_2_O + H]^+^	1.38	1.10	1.32	1.31	tr	1.52	1.42	1.70	2.83	1.08	1.79	2.12
8	Lutein	13.29	444, 472	551 [M-H_2_O + H]^+^	36.50	35.20	35.29	46.68	29.46	63.21	58.52	53.43	76.42	44.48	57.21	67.94
9	(9Z,9′*Z*)-Lutein	14.12	330, 437, 464	551 [M-H_2_O + H]^+^	1.91	1.82	0	2.02	1.27	0	0	0	0	0.32	0	0
10	Zeaxanthin	15.47	450, 475	569 [M + H]^+^	0	0	0	0	7.11	8.54	1.67	1.65	0.95	6.25	6.33	tr
11	Unidentified mixture	16.05			1.98	1.52	0	1.34	0	0	0	0	0	0	0	0
12	(9*Z*)-Lutein	16.77	331, 439, 467	551 [M-H_2_O + H]^+^	12.41	15.31	10.24	9.86	2.73	1.15	4.04	3.44	4.41	3.58	2.66	2.66
13	(9′*Z*)-Lutein	19.62	330, 440, 467	551 [M-H_2_O + H]^+^	27.46	31.40	28.73	21.22	29.79	tr	4.60	3.16	1.21	3.01	1.90	2.56
14	α-Cryptoxanthin	21.18	445, 472	553 [M + H]^+^	0.93	1.52	2.40	0.40	2.60	0.73	3.13	2.38	tr	3.08	tr	3.26
15	β-Cryptoxanthin	24.46	450, 476	553 [M + H]^+^	tr	tr	0.34	0	4.28	tr	1.35	4.70	0	1.07	tr	2.26
16	β-Carotene 5,6-epoxide	28.30	445, 471	553 [M + H]^+^	tr	tr	tr	0	tr	tr	3.06	5.09	0	tr	tr	tr
17	α-Carotene	31.51	445, 472	537 [M + H]^+^	tr	tr	tr	0	tr	tr	5.55	5.87	tr	17.85	1.85	3.63
18	β-Carotene	34.84	451, 476	537 [M + H]^+^	2.24	1.73	2.35	1.62	6.48	0.28	3.73	5.69	0.96	7.01	5.69	3.97
19	(9*Z*)-β-Carotene	35.42	444, 470	537 [M + H]^+^	tr	0.26	0.38	0.22	0.90	0	1.13	1.67	0.39	2.11	0.92	tr
	Total carotenoid mg/g				0.134	0.283	0.124	0.263	0.131	0.937	0.293	0.135	0.126	0.075	0.148	0.299
	Plant parts analyzed				i	i	i	i	i	Rayflorets	i	Rayflorets	Rayflorets	Rayflorets	Sepals	Bracts
	Collection site				a	b 2021	b 2022	c	b	d	b	a	b	a	e	f
Peak No.	Carotenoid	Retention Time (min)	UV-Vis λ_max_ (nm)	Plant Source: MS (*m/z*)	Shining Spurge *(Euphorbia lucida)*	Marsh Spurge *(Euphorbia palustris)*	Cushion Spurge *(Euphorbia polychroma)*	Yellow Tuft (*Alyssum murale*)	Warty Cabbage (*Bunias orientalis)*	Wallflower *(Erysimum cheiri*)	Austrian Yellowcress (*Rorippa austriaca)*	Wormwood Senna (*Cassia artmisioides*)		Ice Plant *(Glottiphyllum cruciatum)*	Autumn Crocus *(Colchicum autumnale)*	Autumn Daffodil *(Sternbergia lutea)*
1	Neoxanthin	6.77	416, 440, 468	601 [M + H]^+^	1.06	4.18	0	3.40	5.38	1.25	0.73	1.54		tr	0.14	tr
2	Violaxanthin	7.49	416, 440, 468	601 [M + H]^+^	4.87	8.50	0	14.46	6.66	1.93	7.08	1.15		tr	1.48	13.31
3	(9′*Z*)-Neoxanthin	8.35	411, 434, 463	601 [M + H]^+^	1.03	1.12	0	1.43	2.89	0.59	6.69	0		tr	1.03	0.54
4	Unidentified mixture	9.16			1.88	0	0	1.70	2.53	7.91	8.06	0		tr	0	2.75 *
5	(9*Z*)-Violaxanthin	11.01	411, 434, 463	601 [M + H]^+^	7.43	6.76	0	9.94	7.93	2.25	0	0		tr	2.73	3.01
6	(13*Z*)-Lutein	11.87	331, 435, 463	551 [M-H_2_O + H]^+^	1.81	1.83	8.97	0	0	2.82	0	1.64		4.87	4.06	1.95
7	(13′*Z*)-Lutein	12.35	331, 438, 464	551 [M-H_2_O + H]^+^	1.97	1.45	5.73	1.42	1.46	1.88	1.99	0.76		2.13	3.47	1.36
8	Lutein	13.29	444, 472	551 [M-H_2_O + H]^+^	69.28	53.91	61.97	55.96	55.75	58.56	52.24	37.64		52.46	65.42	65.84
9	(9Z,9′*Z*)-Lutein	14.12	330, 437, 464	551 [M-H_2_O + H]^+^	0	0	0	0	0	0	0	0		0	13.34	0
10	Zeaxanthin	15.47	450, 475	569 [M + H]^+^	2.11	1.55	1.74	tr	tr	0	0	1.59		4.99	0	2.61
11	Unidentified mixture	16.05			0	0	0.18	tr	0	0	1.36	tr		0	0	0
12	(9*Z*)-Lutein	16.77	331, 439, 467	551 [M-H_2_O + H]^+^	3.90	4.12	4.87	3.74	5.86	2.66	4.60	2.25		10.44	5.86	2.35
13	(9′*Z*)-Lutein	19.62	330, 440, 467	551 [M-H_2_O + H]^+^	1.68	3.62	1.05	3.10	1.52	1.22	10.28	0.56		9.37	0.46	1.33
14	α-Cryptoxanthin	21.18	445, 472	553 [M + H]^+^	0.54	0.65	0.93	0.30	2.15	2.21	2.10	15.18		1.62	0.91	0.84
15	β-Cryptoxanthin	24.46	450, 476	553 [M + H]^+^	1.44	0.86	tr	tr	tr	0.41	0.10	0		0.40	tr	0.12
16	β-Carotene 5,6-epoxide	28.30	445, 471	553 [M + H]^+^	0.48	0.30	tr	tr	tr	0.61	tr	0		0.70	tr	tr
17	α-Carotene	31.51	445, 472	537 [M + H]^+^	0.17	1.64	tr	tr	tr	7.09	0.23	4.42		0.44	0.15	0.15
18	β-Carotene	34.84	451, 476	537 [M + H]^+^	7.48	6.71	2.96	3.93	6.17	6.49	0.75	27.54		6.57	0.18	2.06
19	(9*Z*)-β-Carotene	35.42	444, 470	537 [M + H]^+^	1.28	1.72	0.88	0.61	1.24	0.71	0.16	3.37		2.01	tr	0.82
	Total carotenoid mg/g				0.253	0.141	0.266	0.432	0.208	0.492	0.951	0.289		0.029	0.344	0.393
	Plant parts analyzed				Bracts	Bracts	Bracts	Petals	Petals	Petals	Petals, Stamina	Petals		Petals	Stamina	Sepals
	Collection site				g	h	b	b	i	b	j	b		b	d	f

* Antheraxanthin at 10.75 min.; tr: traces; i: inflorescence. Collection sites: a. Brno bot. gar., b. Pécs Melius, c. Kővágószőlős, d. Szentlőrinc, e. Zasavica, f. Bratislava bot. gar., g. Szolnok, h. Lankóci Forest, i. Csíksomlyó, j. Battonya.

## Data Availability

The data presented in this study are available in Appendix A of this article and in the Supplementary Materials.

## Supplementary Materials

The following supporting information can be downloaded at https://www.mdpi.com/article/10.3390/molecules28031187/s1, Figure S1: Structure of carotenoids; Figure S2: NMR spectra of lutein isomers in CDCl_3_ (500/125 MHz for ^1^H/^13^C); Figure S3: UV-vis spectra of lutein isomers in the HPLC mobile phase; Figure S4: HPLC-DAD-MS chromatograms.

## Sample Availability

Samples of the compounds are available from the authors.

## Appendix A

**The plant species used belong to the following eight families:**

**Acanthaceae family**

*Pachystachys lutea* Nee: Golden shrimp plant is a subtropical shrub 90–120 cm tall. This species is native to South America. It was collected for the first time in the Amazon region in Brazil [41]. It is commonly used as an ornamental plant [42].

**Aizoaceae** **family**

*Glottiphyllum cruciatum* (Haw.) N.E.Br, Ice plant has succulent fleshy leaves that are broadened at the base, born in pairs, and spread horizontally. The flowers are yellow. This species is native to arid areas in South Africa [43].

**Amaryllidaceae** **family**

*Sternbergia lutea* (L.) Ker Gawl. ex Spreng, Autumn daffodil is a bulbous plant native to Iran and Turkey. A flower with six bright yellow sepals appears from September to January, depending on the occurrence of rainfall and the temperature of the area [44]. The bulb of this plant contains Amaryllidiaceae alkaloids; thus, it can be potentially used as a medicinal plant [45].

**Asteraceae** **family**

*Anthemis tinctoria* L., Golden marguerite is a herbaceous perennial that blooms from May to September. Its daisy-like head inflorescence (capitulum) consists of ray and disk flowers, which are of a bright yellow color. The species of the Anthemis genus are widely used in pharmaceutics, cosmetics, and the food industry. The flowers have well-documented use as antiseptic and healing herbs, with the main components being natural flavonoids and essential oils [46].

*Coreopsis pubescens* Elliott, Star tickseed is a perennial herbaceous plant that grows up to 120 cm tall. The flower heads are yellow, with both ray florets and disk florets. This plant is native to N. America, where it grows in rocky open woods and gravely stream beds [47].

*Coreopsis verticillata* L., Whorled tickseed is a perennial herbaceous plant that grows up to 100 cm tall. Both the ray florets and disk florets are bright yellow. It is native to North America, where it has been traditionally used as diuretic, while its flowers are edible [48].

*Echinacea paradoxa* (J.B.S. Norton) Britt. The yellow coneflower is a perennial herb and ornamental plant that grows up to 90 cm tall. One plant can form one or more bright yellow flower heads, each with yellow ray florets and yellow disk florets, while all the other Echinacea species native to North America are purple to pink [49,50,51]. It was used by Native Americans as a herb for various ailments, including mouth sores, colds, and cough [52].

*Helianthus angustifolius* L., Narrowleaf sunflower is a perennial herbaceous plant with a 50–150 cm high erect stem and narrowly lanceolate to linear leaves. The capitulum consists of yellow ray florets and disk florets with yellow corolla lobes. The anthers are dark brown or black, and the appendages are dark (style branches are usually yellow). It is native to the south-central and eastern United States, found in all the coastal states from Texas to Long Island [53], and widely used as ornamental plant.

*Helianthus tuberosus* L., Jerusalem artichoke is a perennial herbaceous plant with 150–300 cm high erect stem and lanceolate leaves. The capitulum inflorescence consists of yellow ray florets and disk florets with yellow corolla lobes. It is native to N. America and naturalized in Europe, and it is widely known to have inulin-rich tubers; therefore, it is often used as a crop [54], and sometimes, as an ornamental plant. Recent research has confirmed that it is not only an alternative plant protein source but also a good source of biological valuable phytochemicals [55]. The flowers are consumed as edible parts [56].

*Helichrysum italicum* (Roth) G. Don, Immortelle is a perennial xerophytic plant native to the dry, stony, and sandy areas of the Mediterranean region. This herb has been used in folk medicine due to its bile-promoting, diuretic, and expectorant effects. Studies have shown the antioxidant, antibacterial, antiviral, anti-inflammatory, and anti-proliferative effects of its extracts and essential oil [57].

**Brassicaceae family**

*Alyssum murale* Waldst. & Kit.: Yellowtuft is a herbaceous perennial plant that blooms from May to July, with four small yellow petals. It is native to southern Europe, grows on serpentine soils, and accumulates Ni; therefore, it has been used as a crop for phytoremediation [58].

*Bunias orientalis* L.: A Turkish wartycabbage is a biennial or perennial plant with branched shoots up to 120 cm high. It blooms from May to August. The petals are 4–8 mm long, yellow, entire, or truncate [59]. It is native to the highlands of Armenia, has been introduced to most parts of Europe [60], and is invasive in North America. The high antimicrobial activity of alcoholic extracts of B. orientalis roots against some microbial strains of microorganisms has been shown [61].

*Erysimum cheiri* (L.) Crantz: Wallflower is an evergreen perennial growing to 0.5 m that is native to S. Europe. The flowers have purplish-green sepals and rounded petals that are 2–3 cm long and bright yellow to red and purple [58]. The plant was formerly used mainly as a diuretic and emmenagogue, but recent research has shown that it is more valuable as a cardiotonic. The flowers and stems are antirheumatic, antispasmodic, cardiotonic, nervine, purgative, and resolvent [62].

*Rorippa austriaca* (Crantz) Besser: Austrian yellowcress is a 30–100 cm tall perennial plant with simple elongated leaves and 3–4,5 mm long yellow petals. It blooms from June to August [59]. This species is native to Central South-Eastern Europe, and it has recently been expanding its range to North West Europe and become invasive along riverine areas in its new range.

**Colchicaceae** **family**

*Colchicum autumnale* L.: Autumn crocus is a herbaceous perennial. It has leaves up to 25 cm long. The flowers emerge from the ground long after the leaves have dried. The flowers are solitary, 4–7 cm across, with six pink tepals and six stamens, with orange anthers and three white styles. At the time of fertilization, the ovary is deep below ground. The plant grows widely throughout the northern hemisphere. Colchicum extracts were originally used to treat rheumatic complaints, especially gouty attacks [63].

**Euphorbiaceae family**

*Euphorbia palustris* L.: Marsh spurge is an upright, clump-forming, herbaceous perennial plant that grows up to 150 cm. It occurs on wetlands, and in autumn, the foliage turns orange-red [64].

*Euphorbia lucida* Waldst. et Kit.: The shining spurge is a perennial plant with a thick stem that reaches a height of 130 cm. The dark-green, lanceolate leaves with entire margins are leathery and shiny [65]. It is native to Central Europe, Eastern Central Europe, the Balkan Peninsula, and West Siberia.

*Euphorbia epithymoides* L. (*E. polychroma* A. Kern.): The cushion spurge is a hairy perennial plant that grows up to 50 cm high. It bears terminal cymes of yellow flower heads (cyathia) in spring and summer, which turn orange-red in autumn. It grows on the edge of dry calcareous forests and in steppe meadows [64], and it is native to Central and Southern Europe and the Middle East.

**Fabaceae family**

*Cassia artemisioides* (Gaudich. ex DC.) Randell.: Wormwood senna is an evergreen shrub that grows up to 300 cm. The plant is endemic to Australia and cultivated as an ornamental plant in various countries. It blooms during winter and produces plenty of yellow flowers. Senna roots are used in Ayurvedic medicine for the treatment of skin diseases, leprosy, tuberculous glands, and syphilis, while its fruits are used for the treatment of inflammation, throat troubles, liver complaints, chest complaints, rheumatism, and asthma [65].

**Nymphaceae family**

*Nuphar lutea* (L.) Smith: Water lily is a macrophyte with large leaves and yellow flowers floating on lakes or small rivers. The plant has a thick rhizome, which is anchored in the sediment by roots at depths up to about 250 cm [66].
