# Supplementary material for: Lutein Isomers: Preparation, Separation, Structure Elucidation, and Occurrence in 20 Medicinal Plants
Source: Molecules. 2023 Jan 25;28(3):1187. doi: 10.3390/molecules28031187 (PMC9921531; doi:10.3390/molecules28031187)
Supplement: Supplementary file 1 [file molecules-28-01187-s001.zip › molecules-2175450-supplementary.pdf]

Supplementary materials

Lutein isomers: preparation, separation, structure elucidation and occurrence in twenty medicinal plants

Veronika Nagy [1], Attila Agócs [1], Viktória Lilla Balázs [2], Dragica Purger [2], Rita Filep[2], Viktor Sándor[3], Erika Turcsi [1], Gergely Gulyás-Fekete [1], and József Deli [1,2,*]

[1] Department of Biochemistry and Medical Chemistry, Medical School, University of Pécs, Szigeti út 12., H-7624 Pécs, Hungary
[2] Department of Pharmacognosy, Faculty of Pharmacy, University of Pécs, Rókus u. 2., H-7624 Pécs, Hungary
[3] Institute of Bioanalytics, Medical School, University of Pécs, Szigeti út 12., H-7624 Pécs, Hungary

* Correspondence: jozsef.deli@aok.pte.hu

Figure S1. Structure of carotenoids

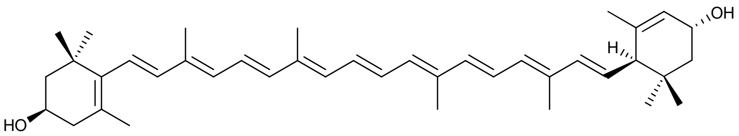

(all-*E*)-Lutein (**1**)

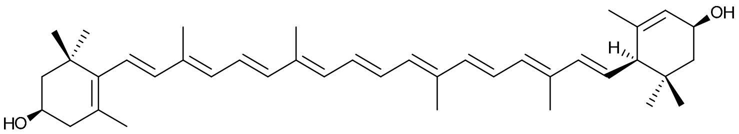

3′-Epilutein (**2**)

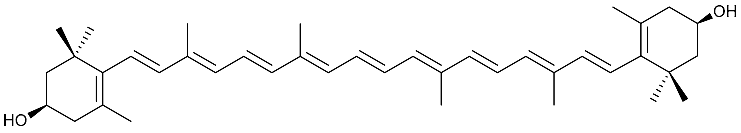

Zeaxanthin (**3**)

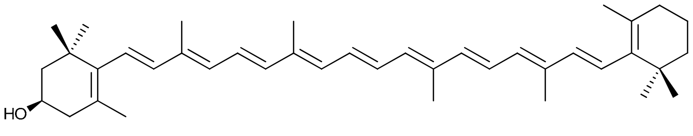

β-Cryptoxanthin (**4**)

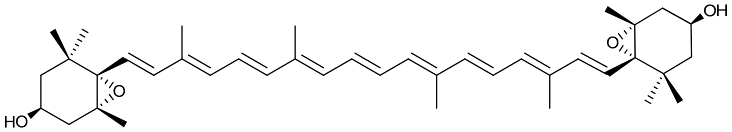

Violaxanthin (**5**)

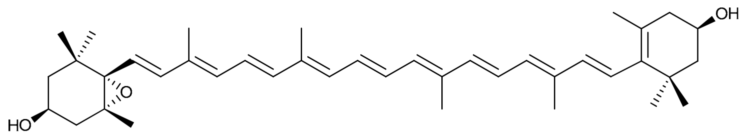

Antheraxanthin (**6**)

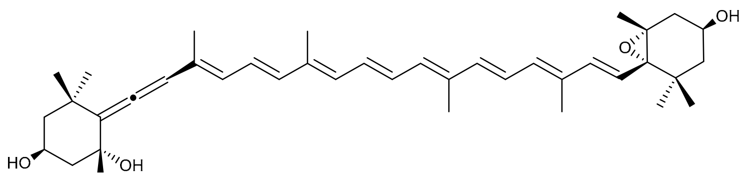

(all-*E*)-Neoxanthin (**7**)

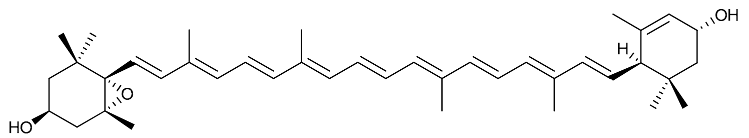

Lutein 5,6-epoxide (**8**)

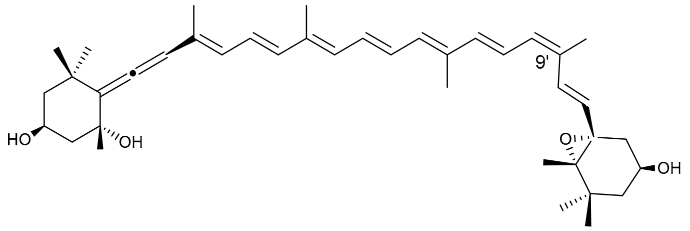

(9′*Z*)-Neoxanthin (**9**)

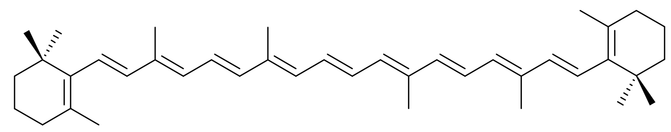

β-Carotene (**10**)

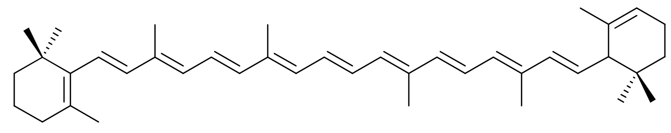

α-Carotene (**11**)

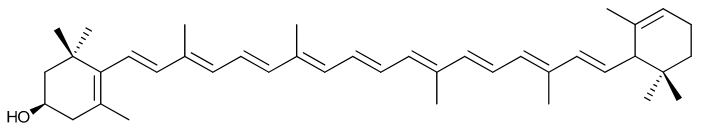

α-Cryptoxanthin (**12**)

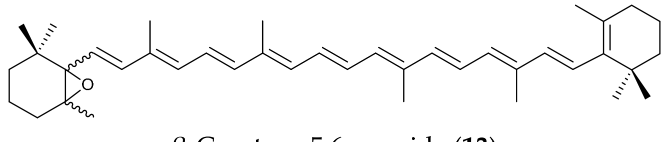

β-Carotene 5,6-epoxide (**13**)

Figure S2. NMR spectra of lutein isomers, in $CDCl_3$, (500/125 MHz for $^1H/^{13}C$)

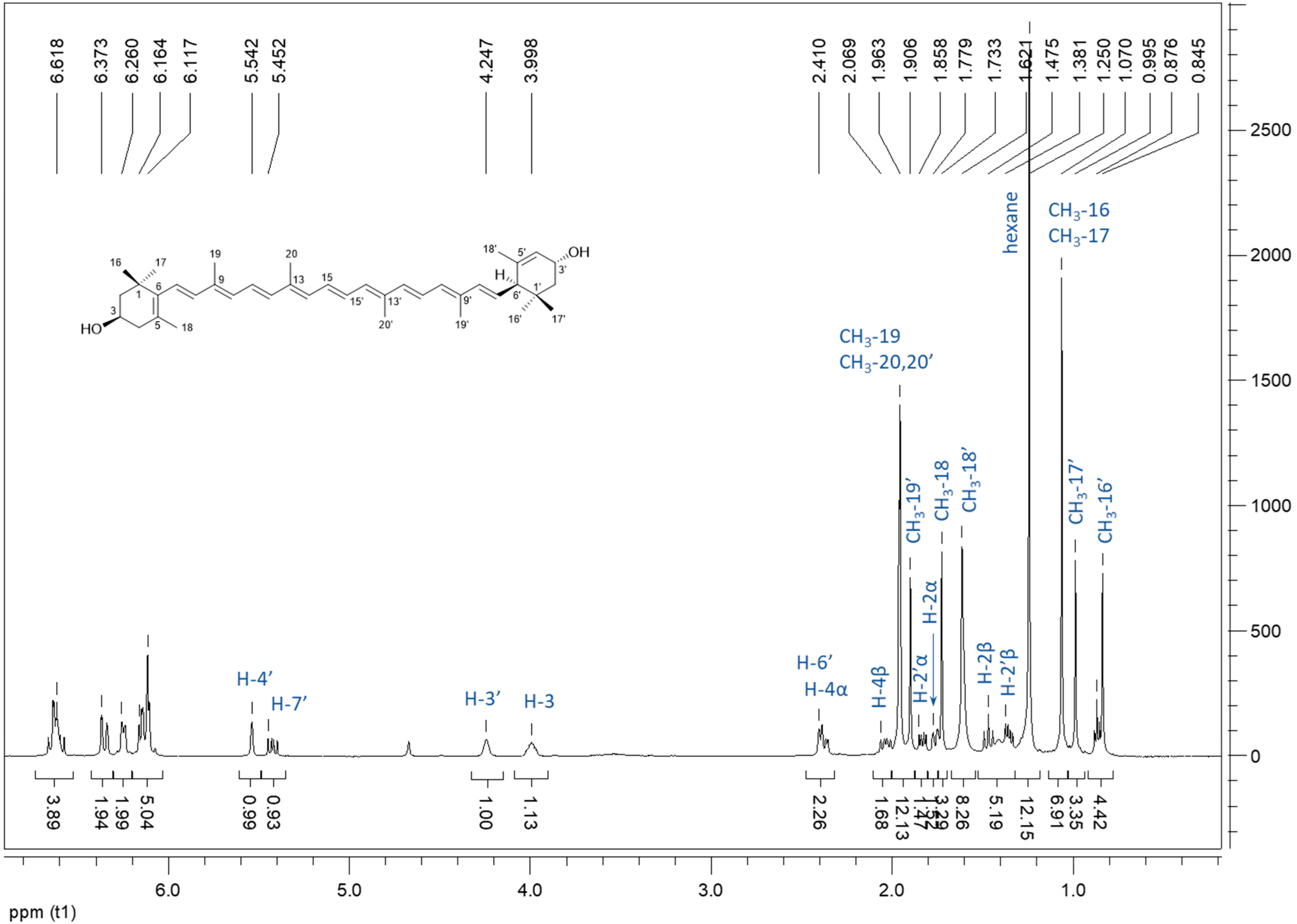

Figure S2.1. ^1H-NMR spectrum of (all-*E*)-lutein in $CDCl_3$, (500/125 MHz for $^1H/^{13}C$)

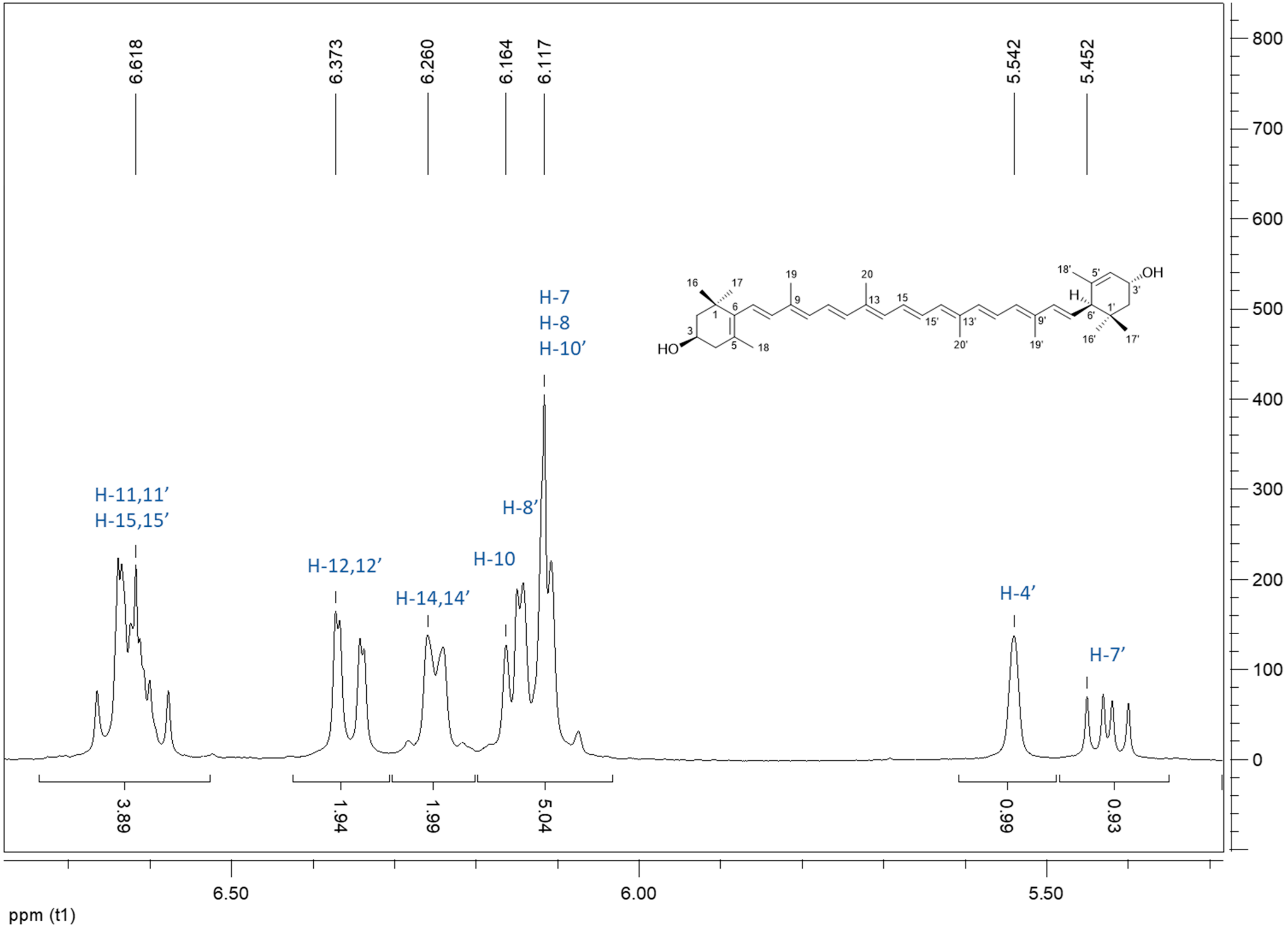

Figure S2.2. ^{1}H-NMR spectrum of (all-*E*)-lutein in $CDCl_3$, (500/125 MHz for ^{1}H/^{13}C)

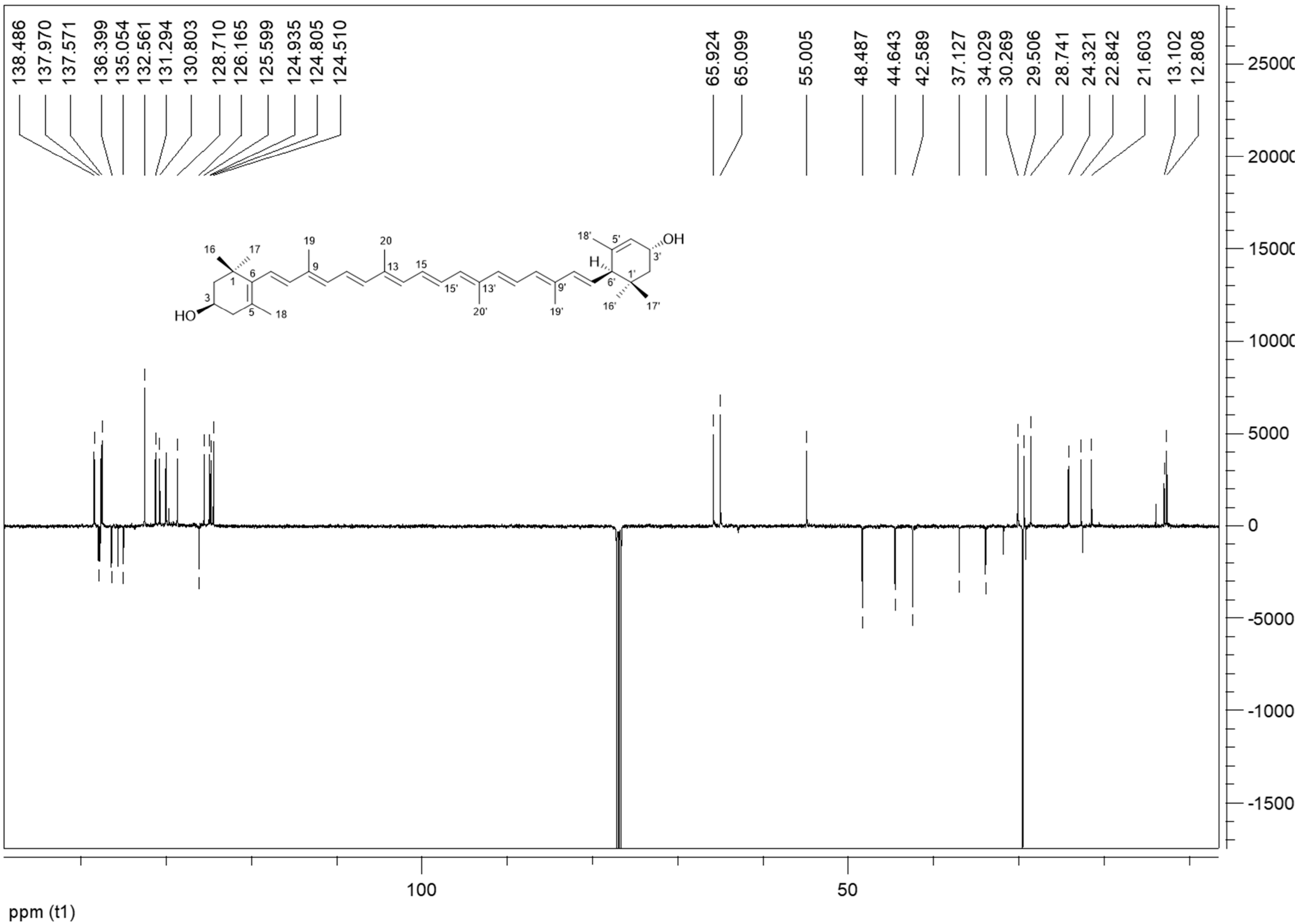

Figure S2.3. ^{13}C-NMR spectrum of (all-*E*)-lutein in $CDCl_3$, (500/125 MHz for ^{1}H/^{13}C)

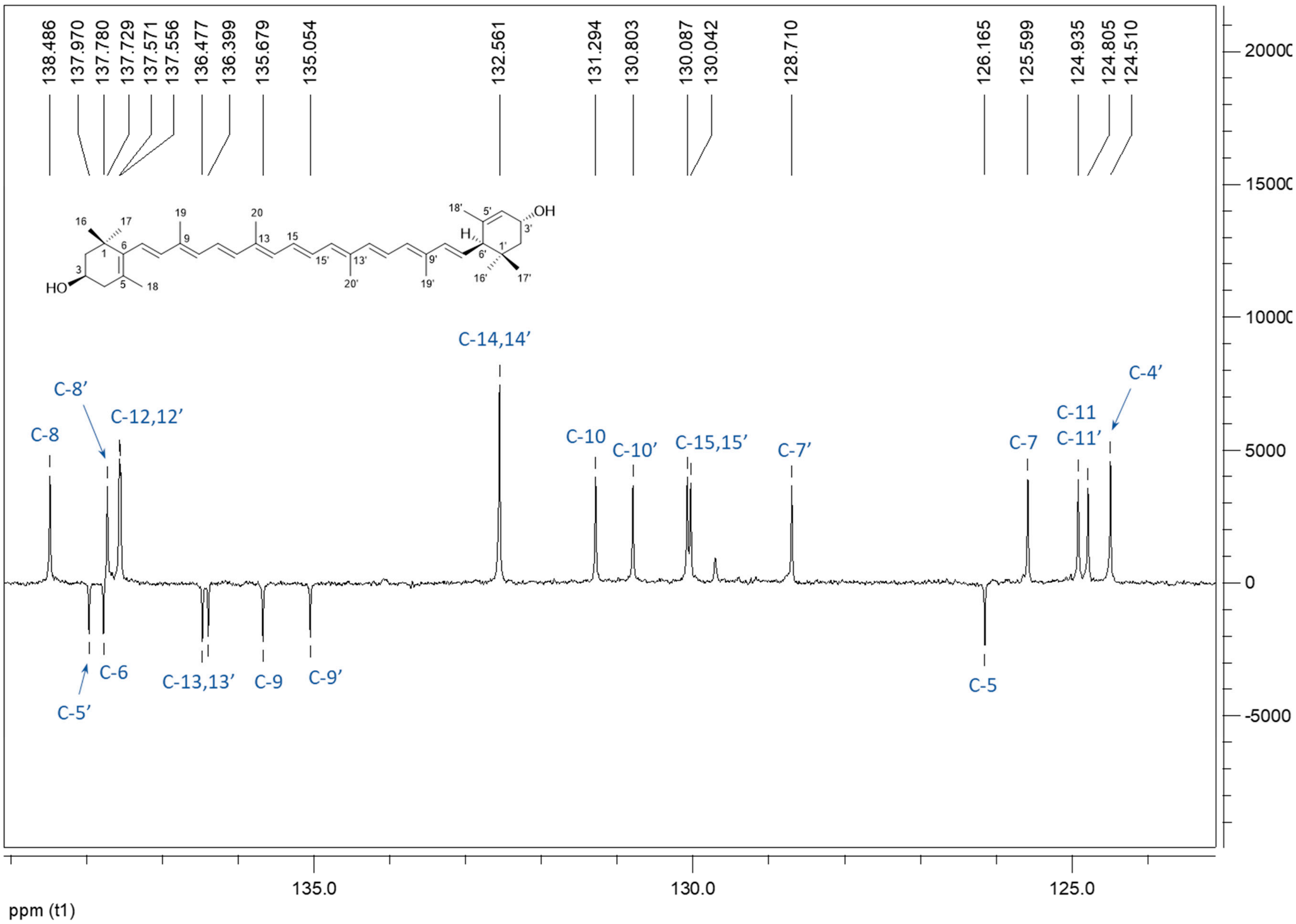

Figure S2.4. ^{13}C-NMR spectrum of (all-*E*)-lutein in $CDCl_3$, (500/125 MHz for ^{1}H/^{13}C)

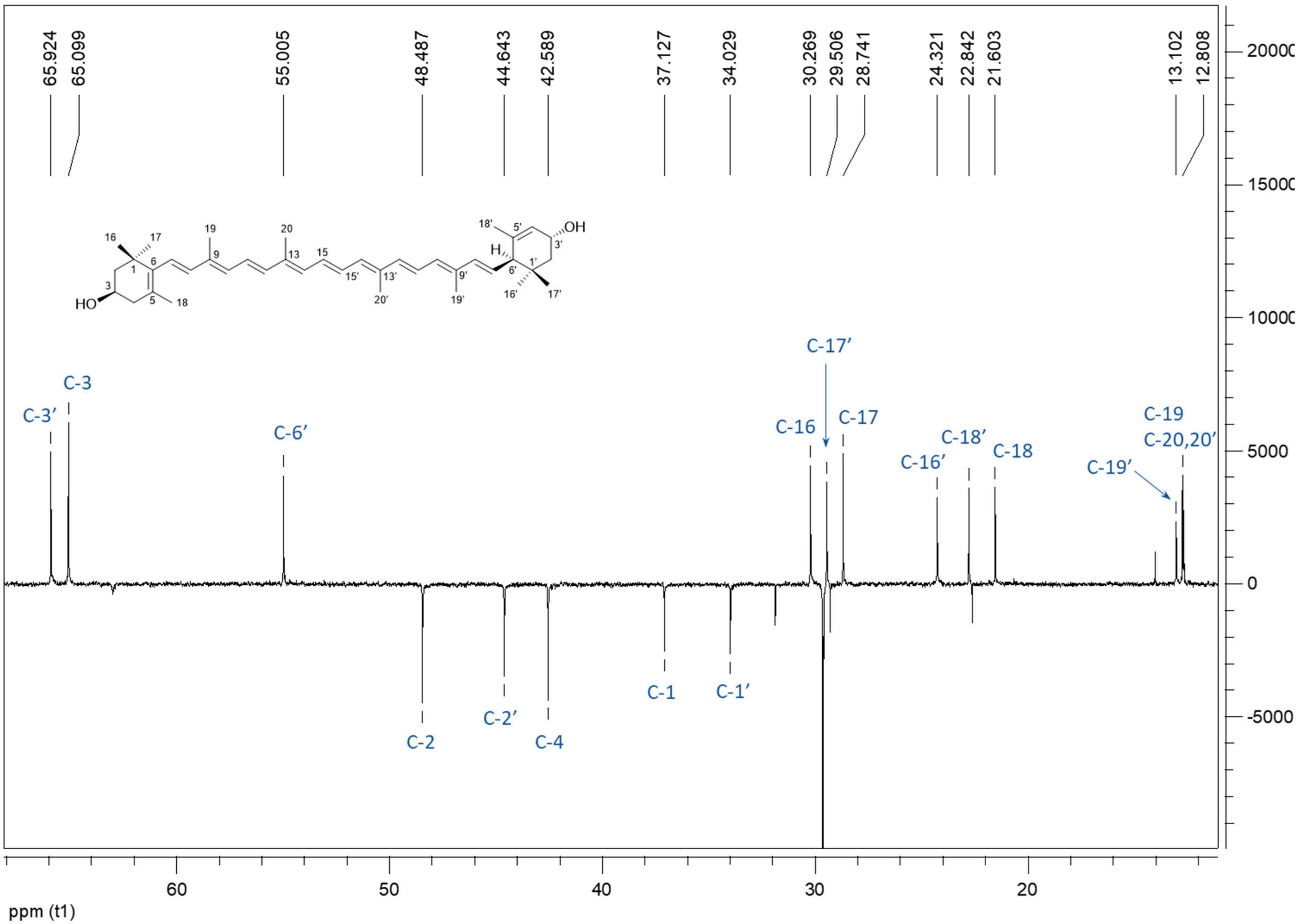

Figure S2.5. ^{13}C-NMR spectrum of (all-*E*)-lutein in $CDCl_3$, (500/125 MHz for ^{1}H/^{13}C)

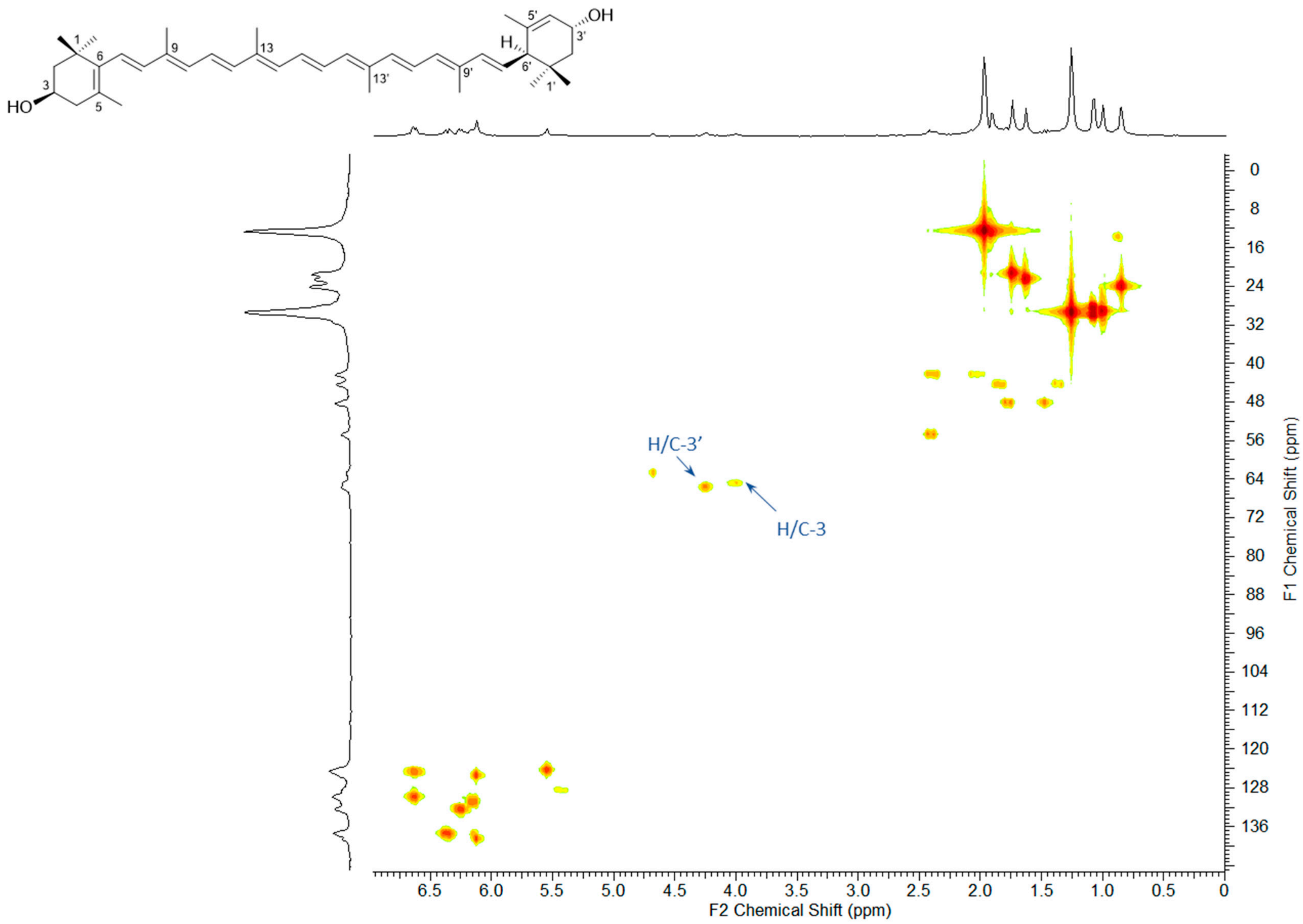

Figure S2.6. ^{13}C-^{1}H-HSQC spectrum of (all-*E*)-lutein in $CDCl_3$, (500/125 MHz for $^{1}H/^{13}C$)

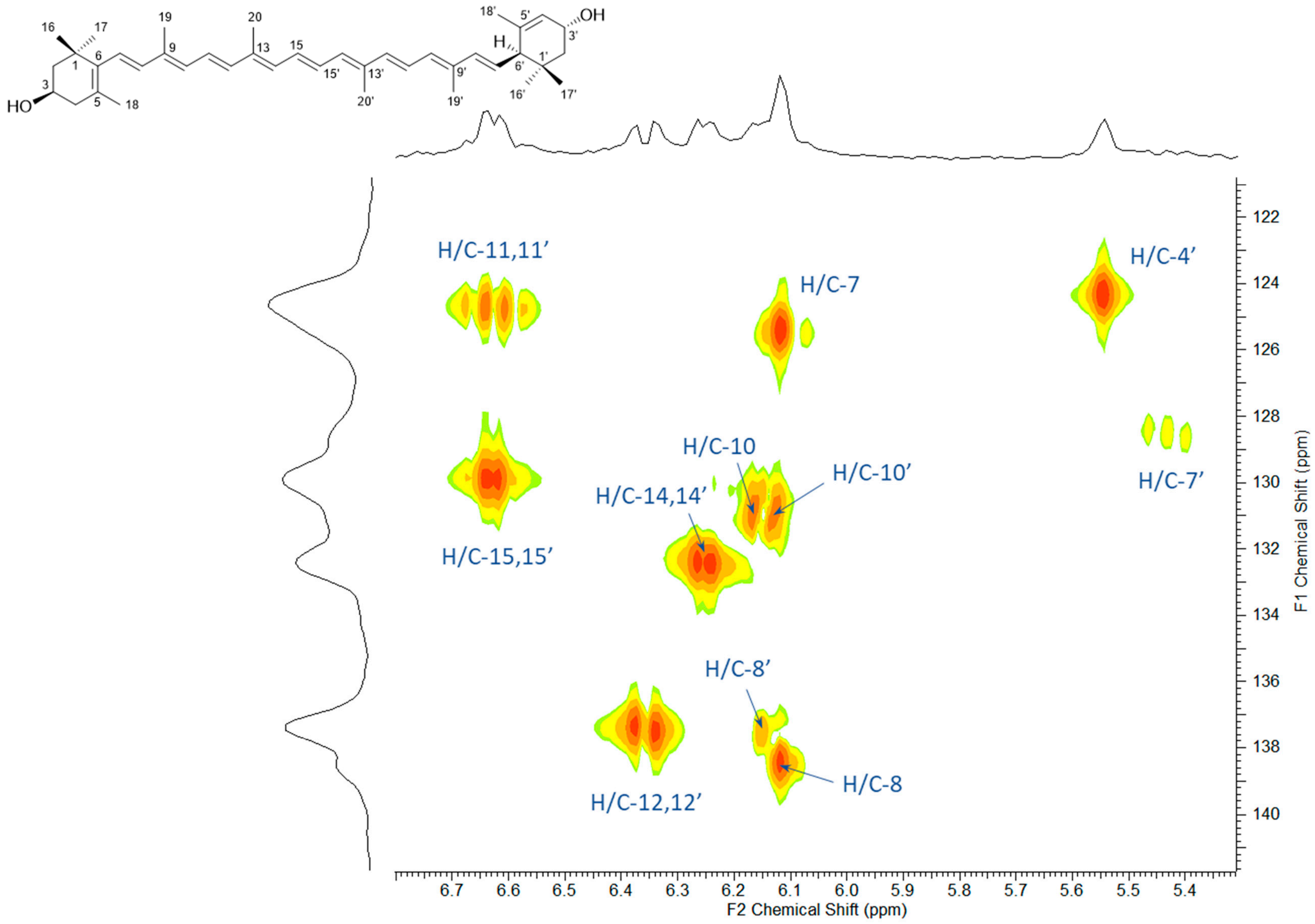

Figure S2.7. ^{13}C-^{1}H-HSQC spectrum of (all-*E*)-lutein in $CDCl_3$, (500/125 MHz for ^{1}H/^{13}C)

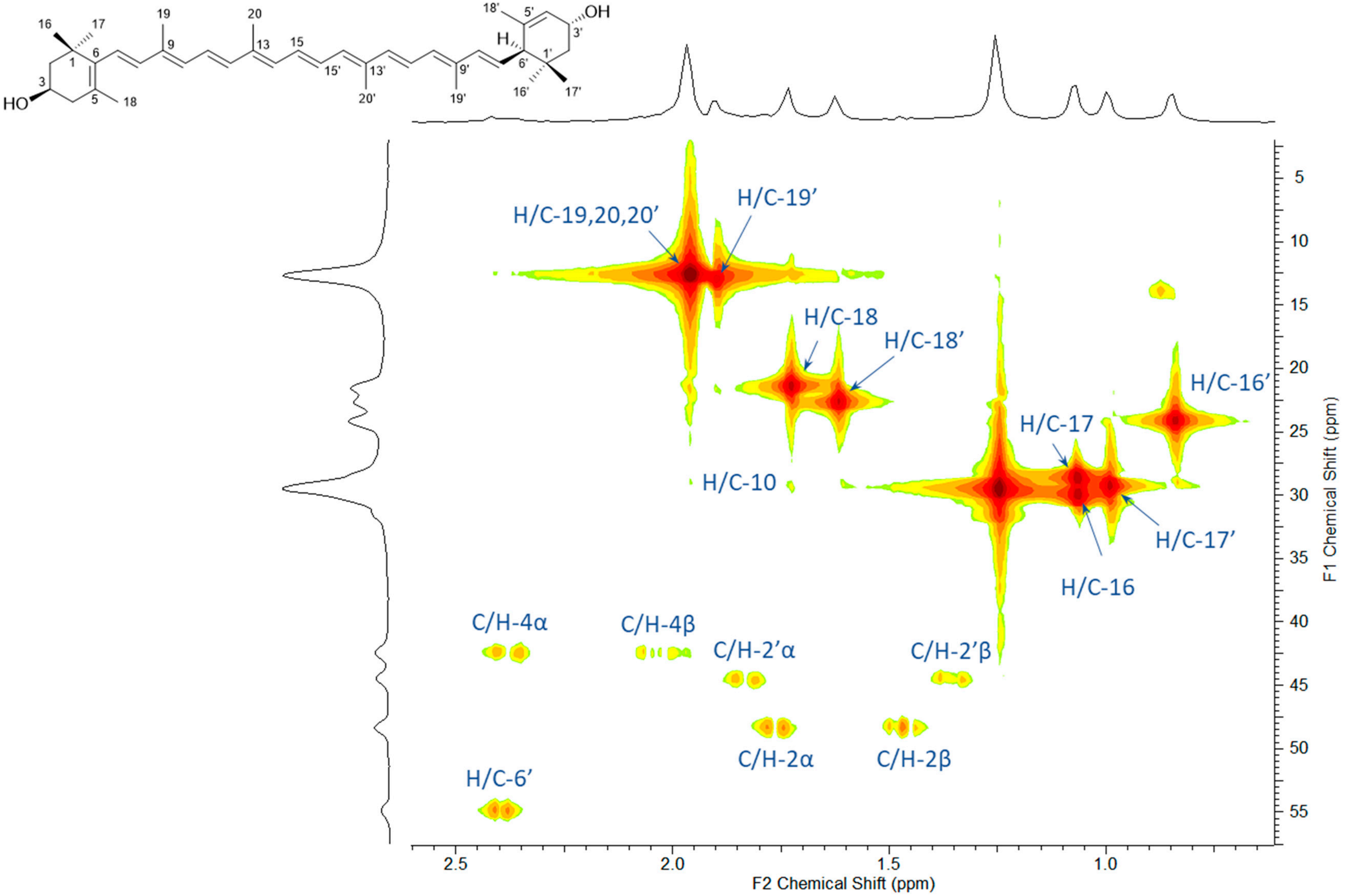

Figure S2.8. ^{13}C-^{1}H-HSQC spectrum of (all-*E*)-lutein in $CDCl_3$, (500/125 MHz for ^{1}H/^{13}C)

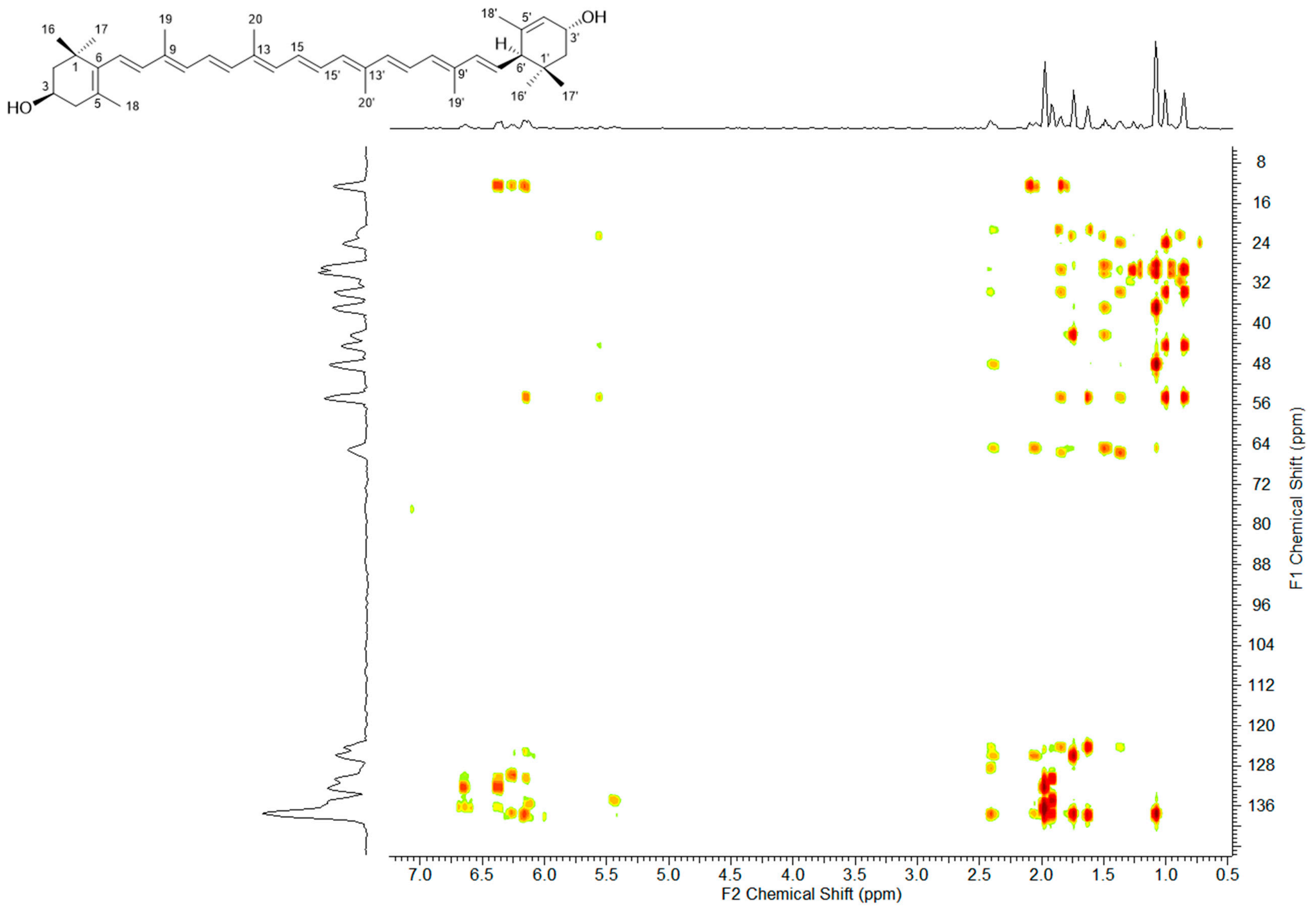

Figure S2.9. ^{13}C-^{1}H-HMBC spectrum of (all-*E*)-lutein in CDCl$_3$, (500/125 MHz for ^{1}H/^{13}C)

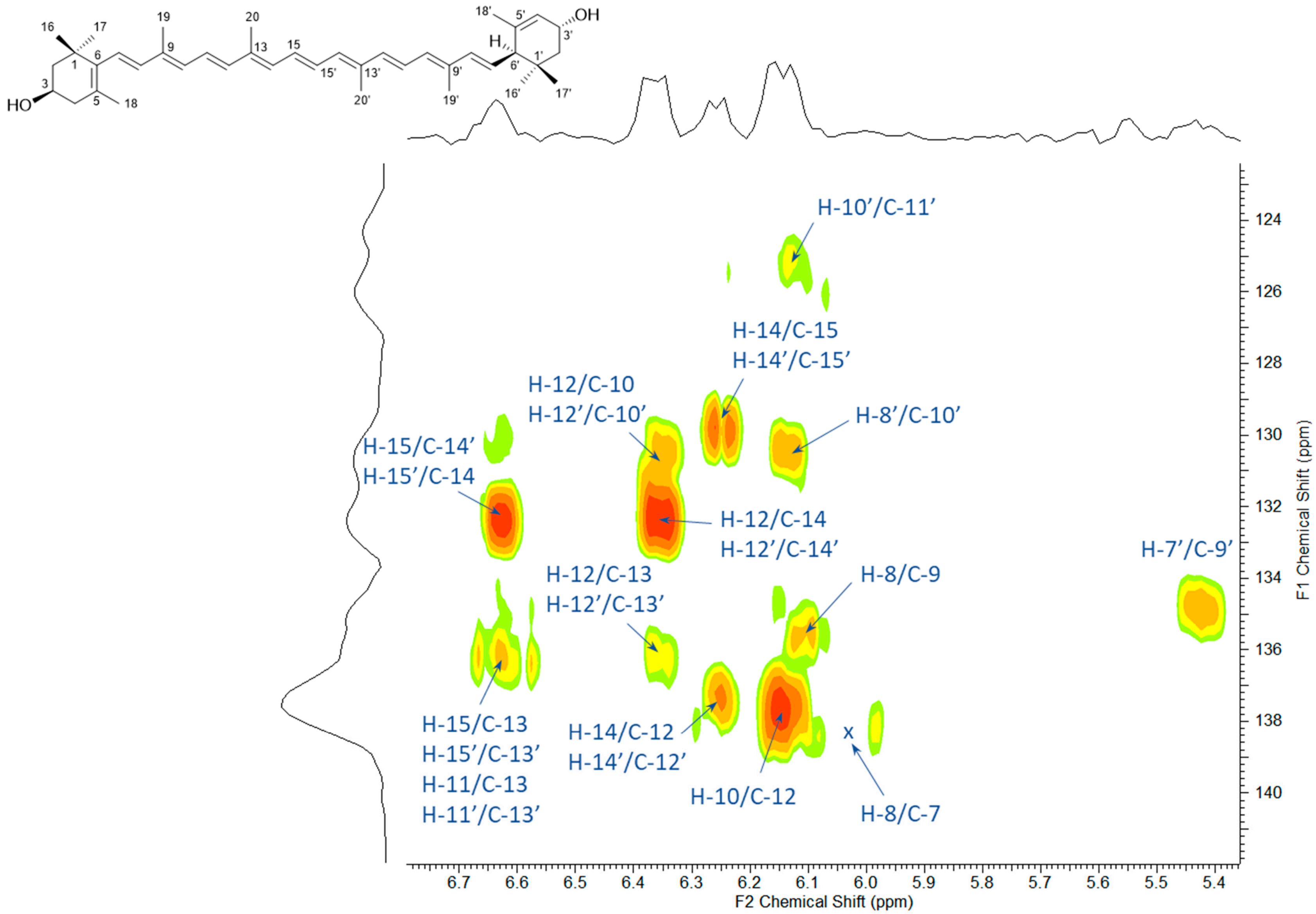

Figure S2.10. ^{13}C-^{1}H-HMBC spectrum of (all-*E*)-lutein in $CDCl_3$, (500/125 MHz for ^{1}H/^{13}C)

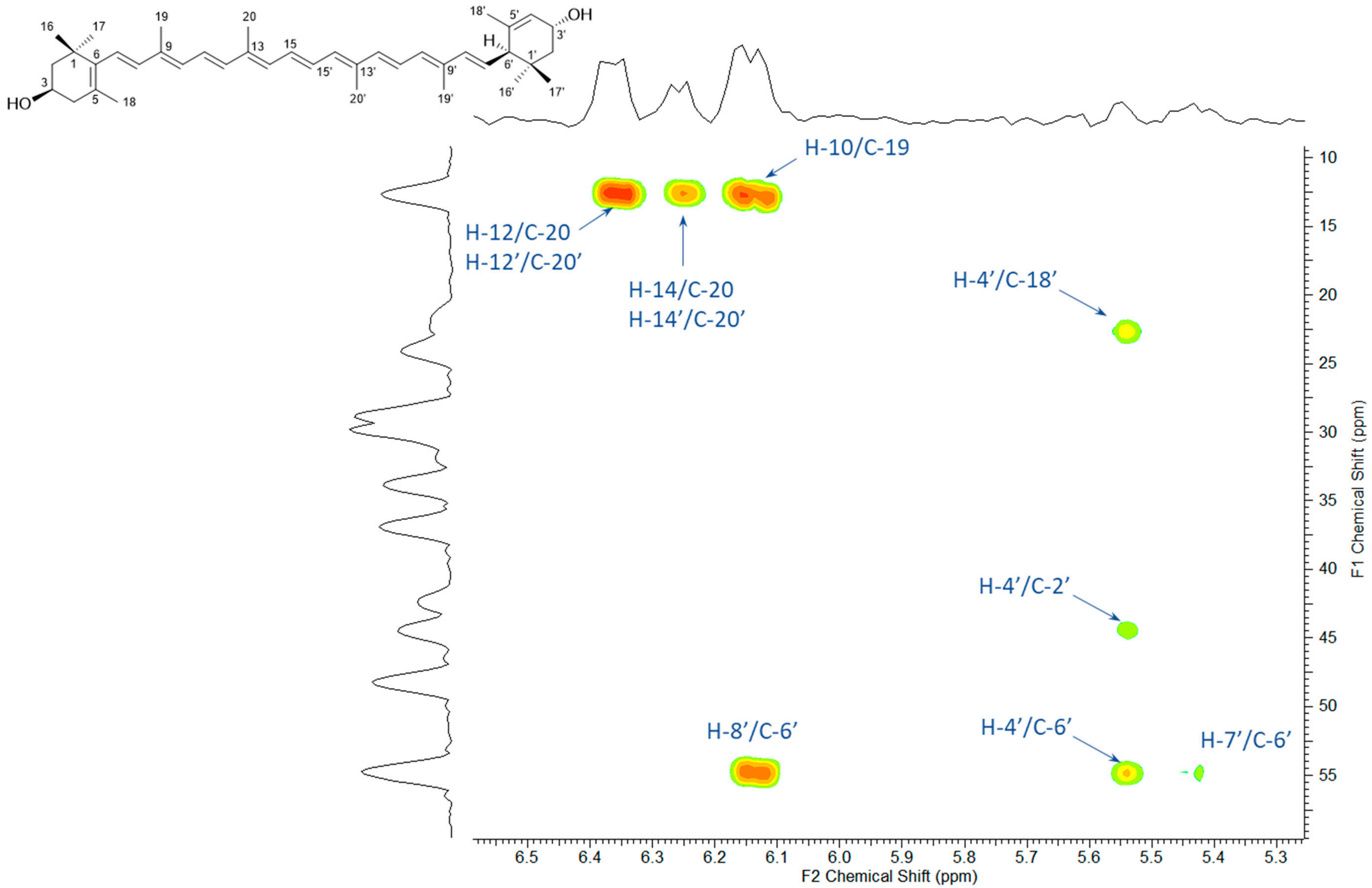

Figure S2.11. ^{13}C-^{1}H-HMBC spectrum of (all-*E*)-lutein in $CDCl_3$, (500/125 MHz for ^{1}H/^{13}C)

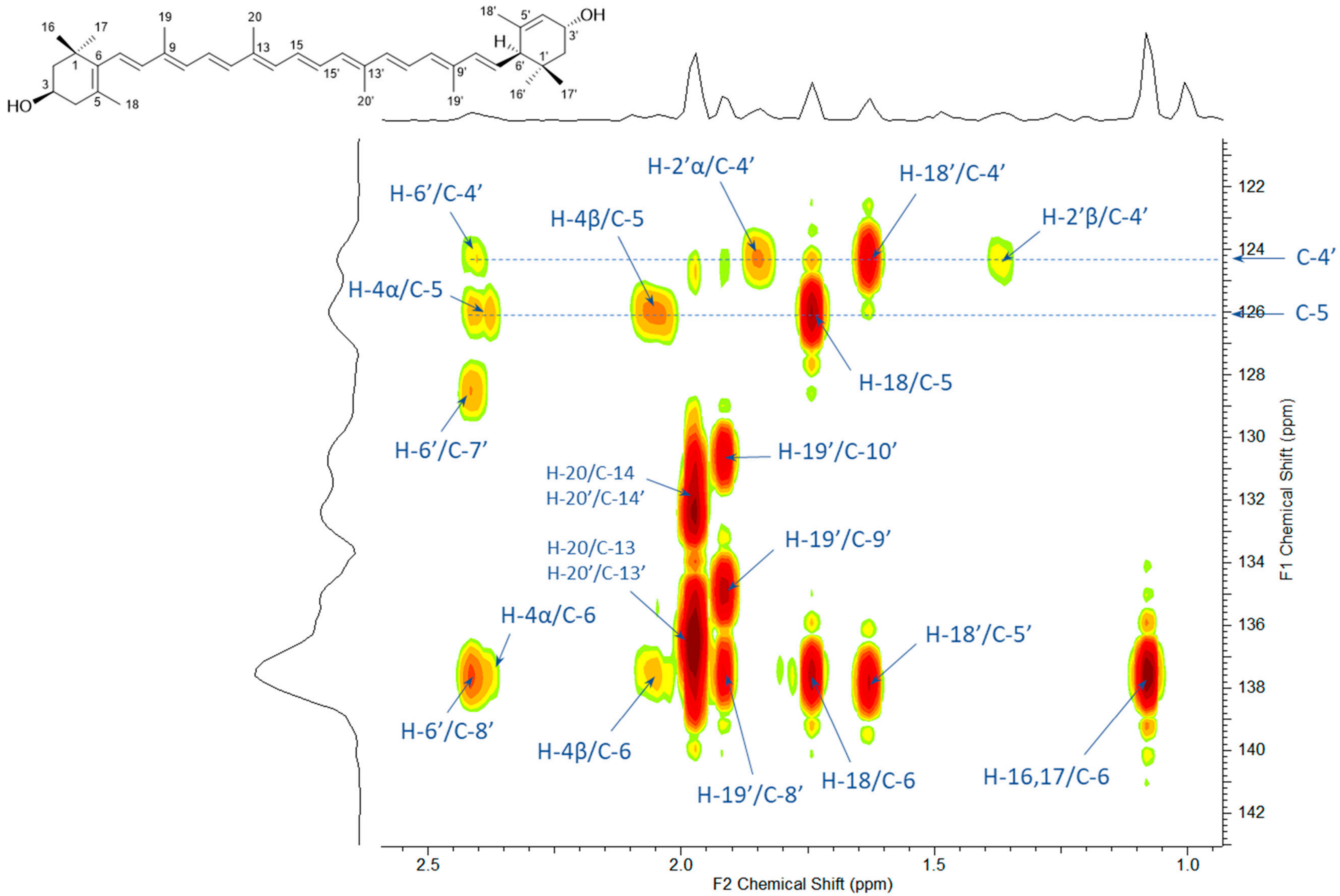

Figure S2.12. ^{13}C-^{1}H-HMBC spectrum of (all-*E*)-lutein in $CDCl_3$, (500/125 MHz for ^{1}H/^{13}C)

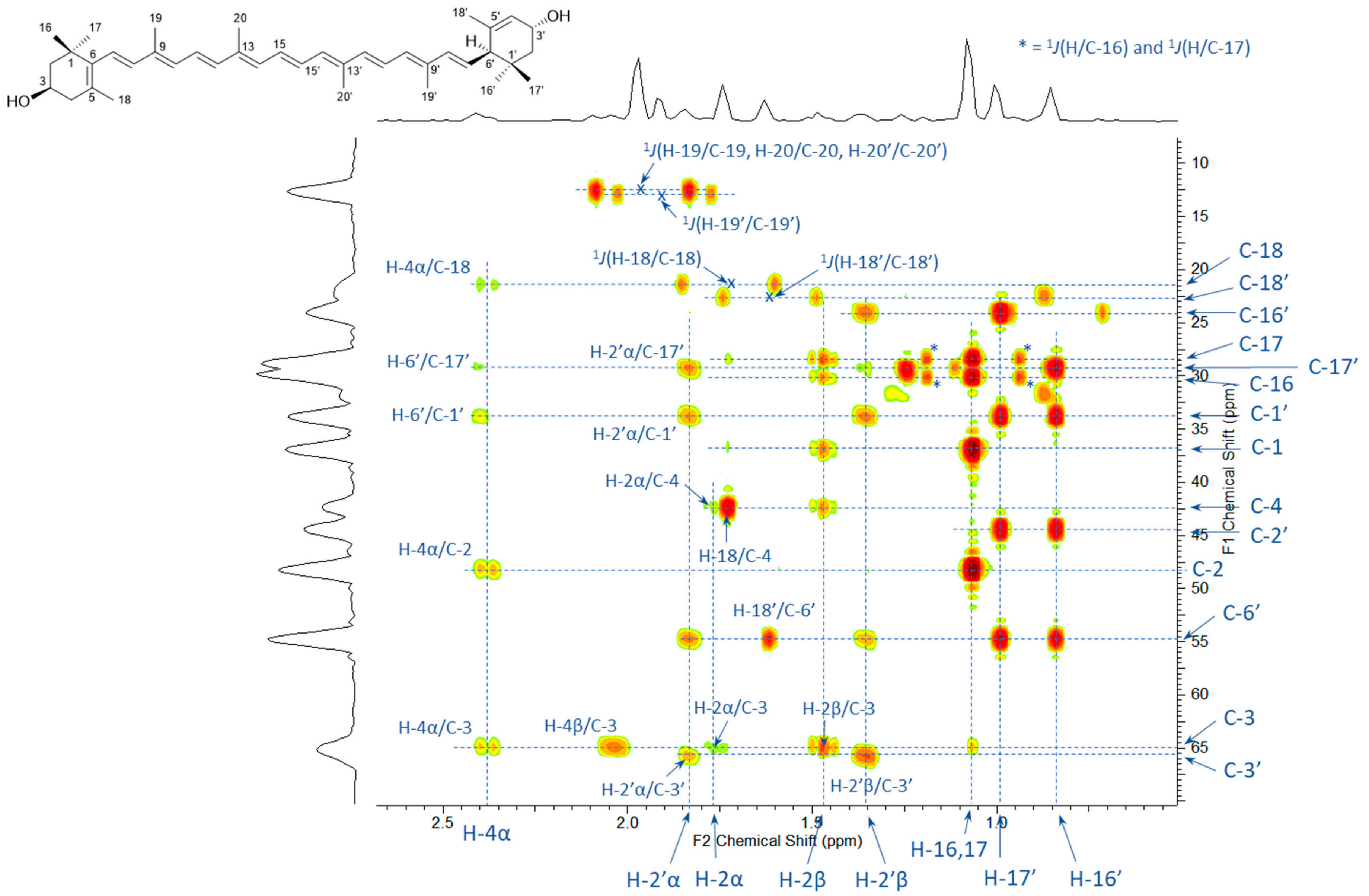

Figure S2.13. ^{13}C-^{1}H-HMBC spectrum of (all-*E*)-lutein in $CDCl_3$, (500/125 MHz for ^{1}H/^{13}C)

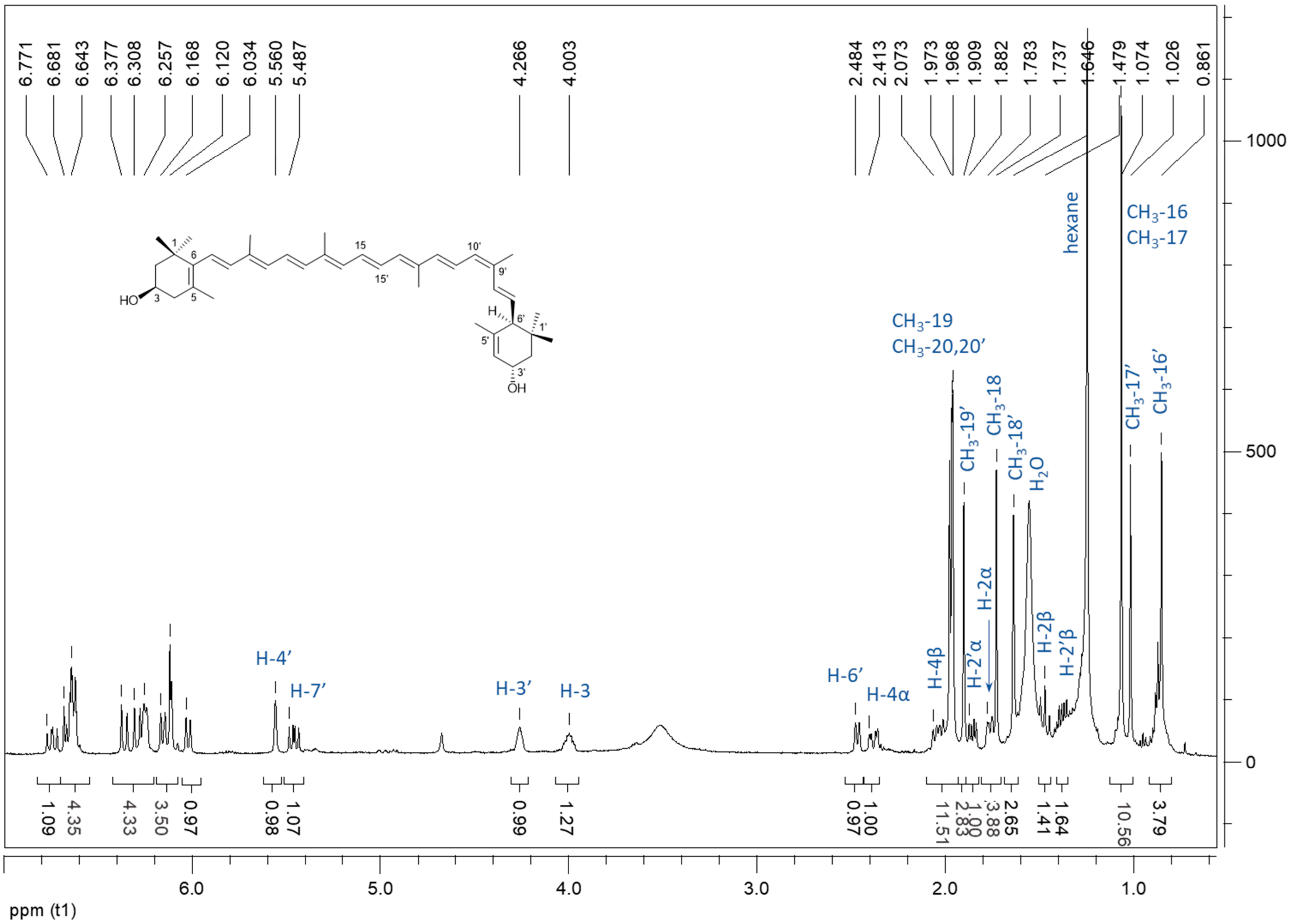

Figure S2.14. ¹H-NMR spectrum of (9′Z)-lutein in $CDCl_3$, (500/125 MHz for ¹H/¹³C)

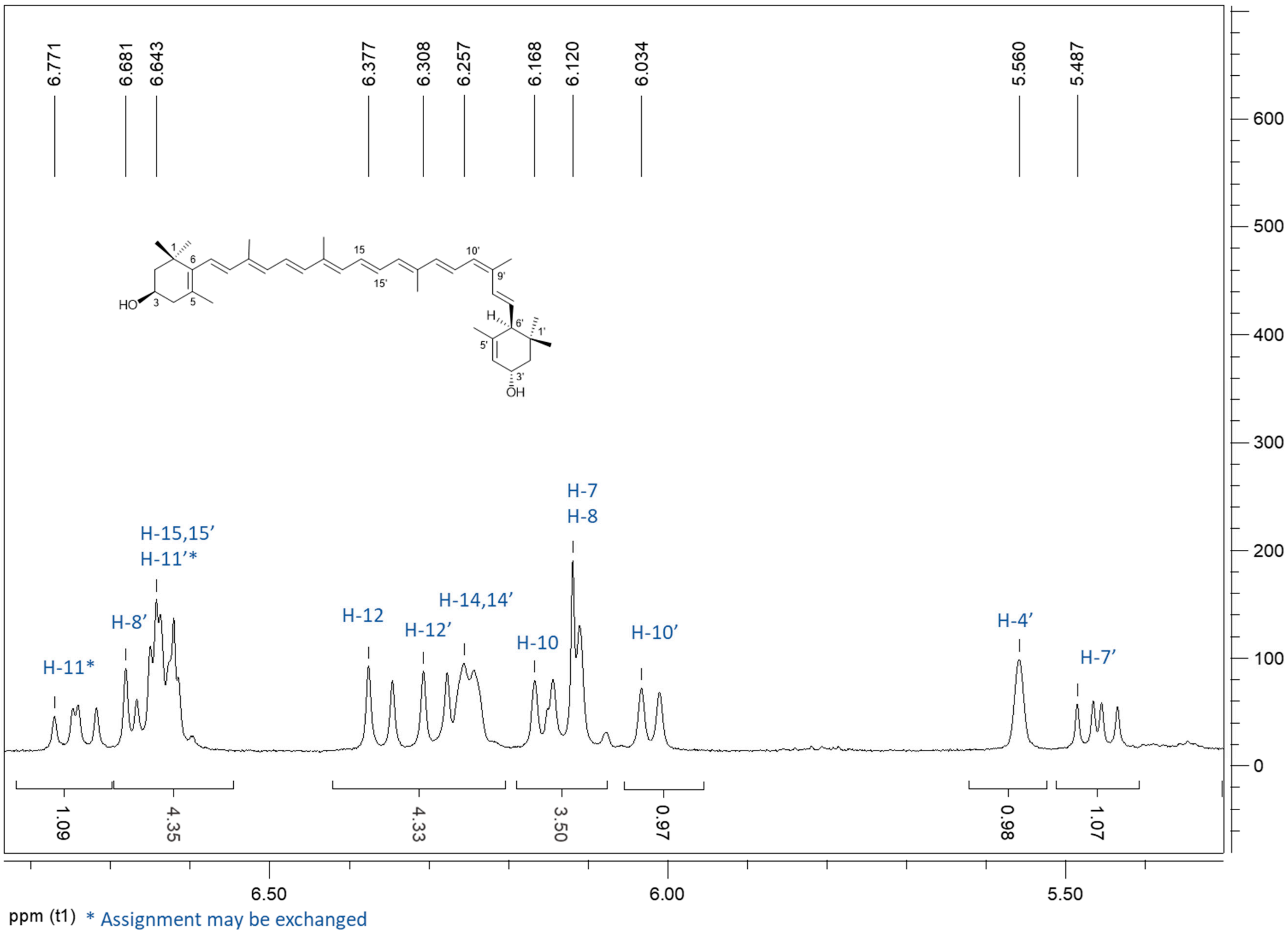

Figure S2.15. ^{1}H-NMR spectrum of (9′Z)-lutein in $CDCl_3$, (500/125 MHz for $^{1}H/^{13}C$)

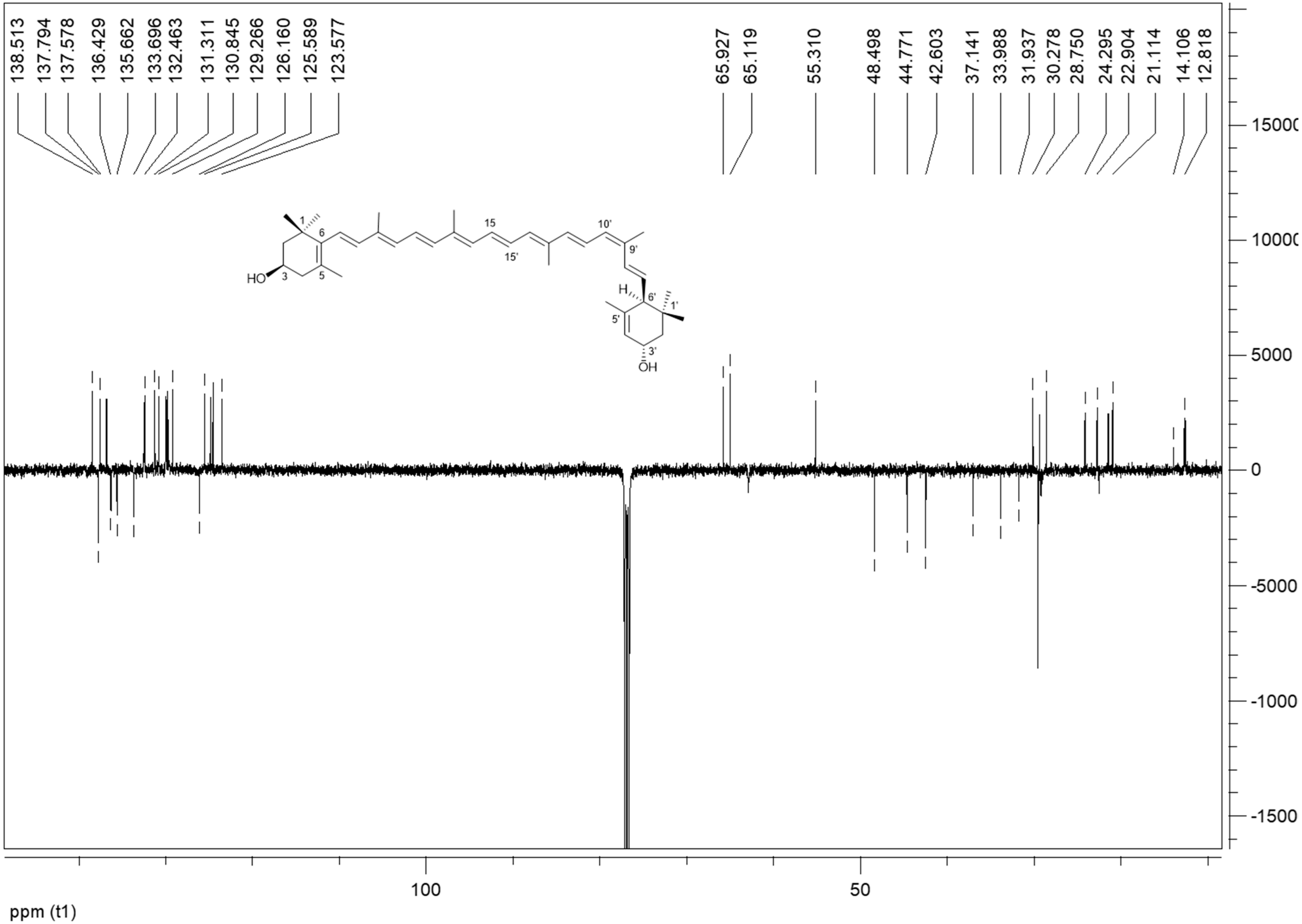

Figure S2.16. ^{13}C-NMR spectrum of (9′Z)-lutein in $CDCl_3$, (500/125 MHz for ^{1}H/^{13}C)

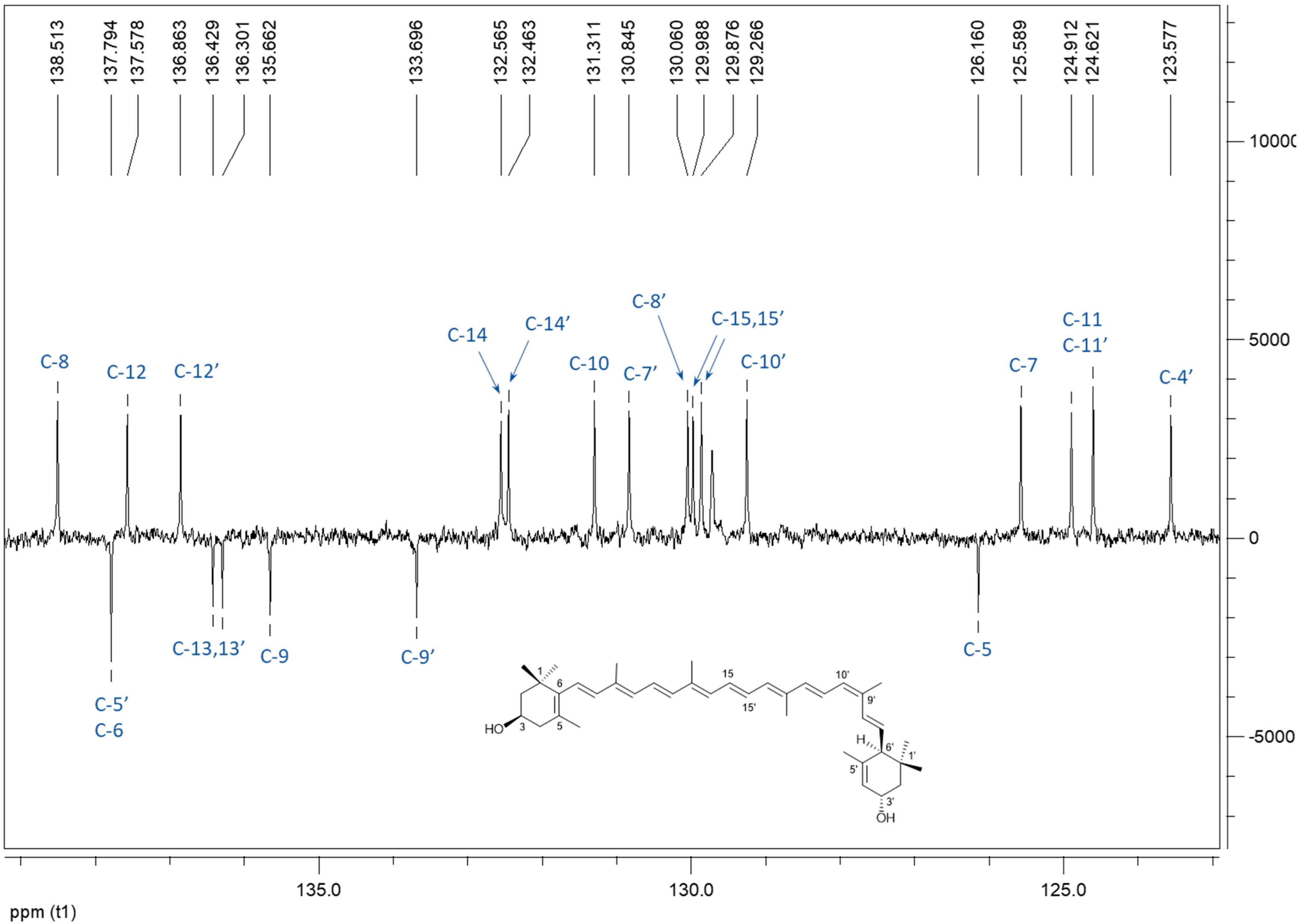

Figure S2.17. ^{13}C-NMR spectrum of (9′*Z*)-lutein in $CDCl_3$, (500/125 MHz for ^1H/^{13}C)

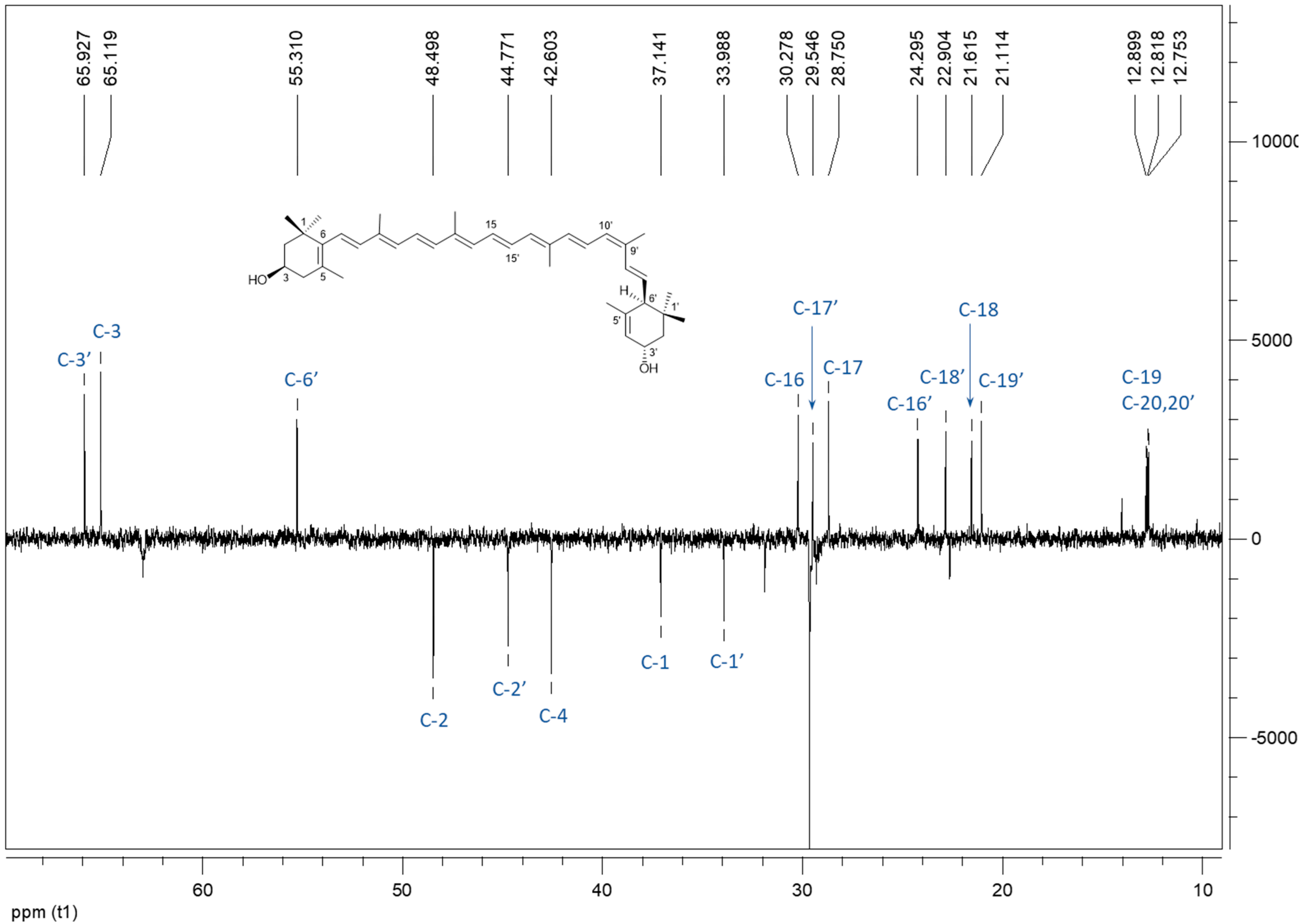

Figure S2.18. ^{13}C-NMR spectrum of (9′Z)-lutein in $CDCl_3$, (500/125 MHz for $^{1}H/^{13}C$)

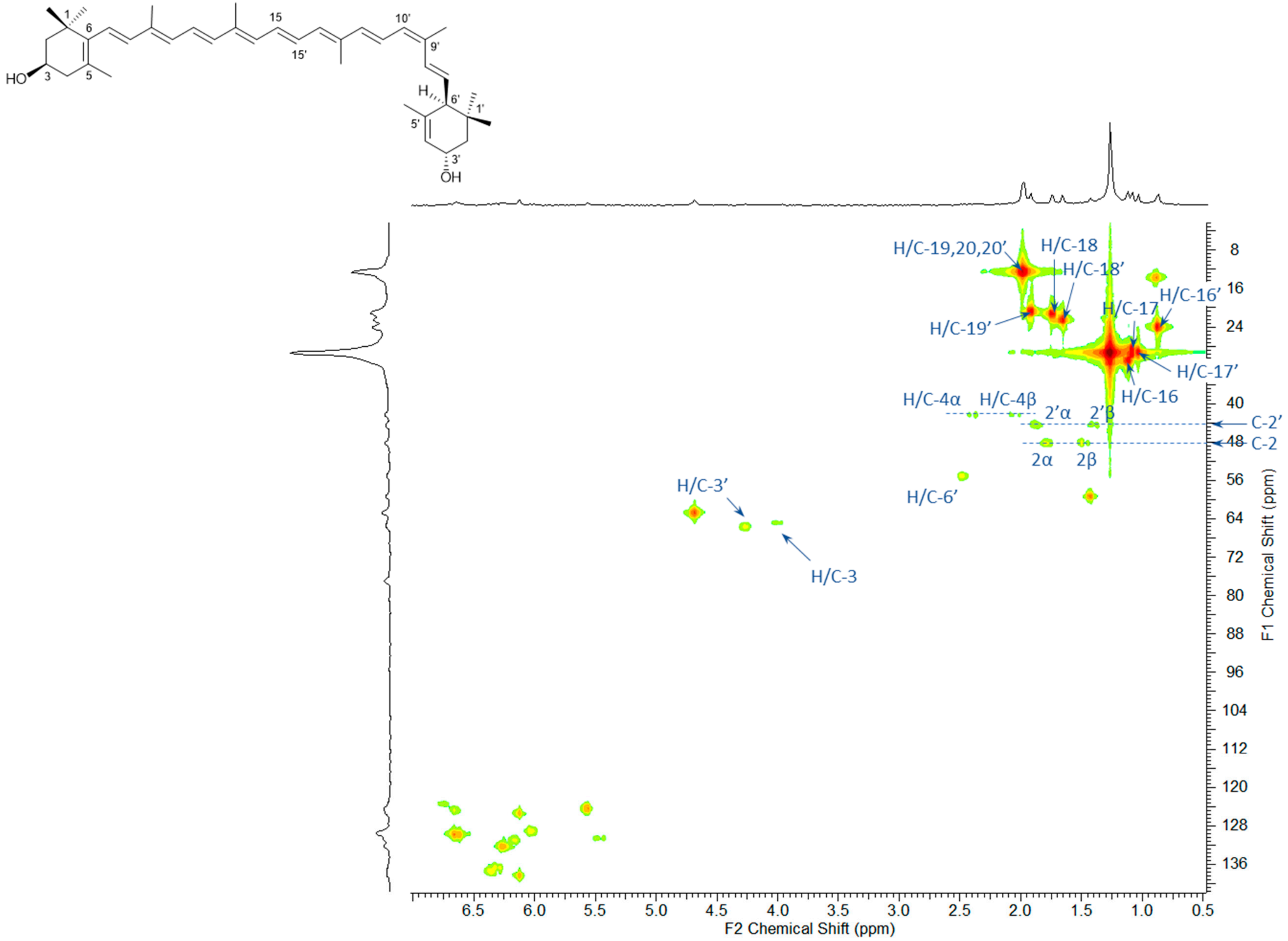

Figure S2.19. ^{13}C-^{1}H-HSQC spectrum of (9′*Z*)-lutein in $CDCl_3$, (500/125 MHz for ^{1}H/^{13}C)

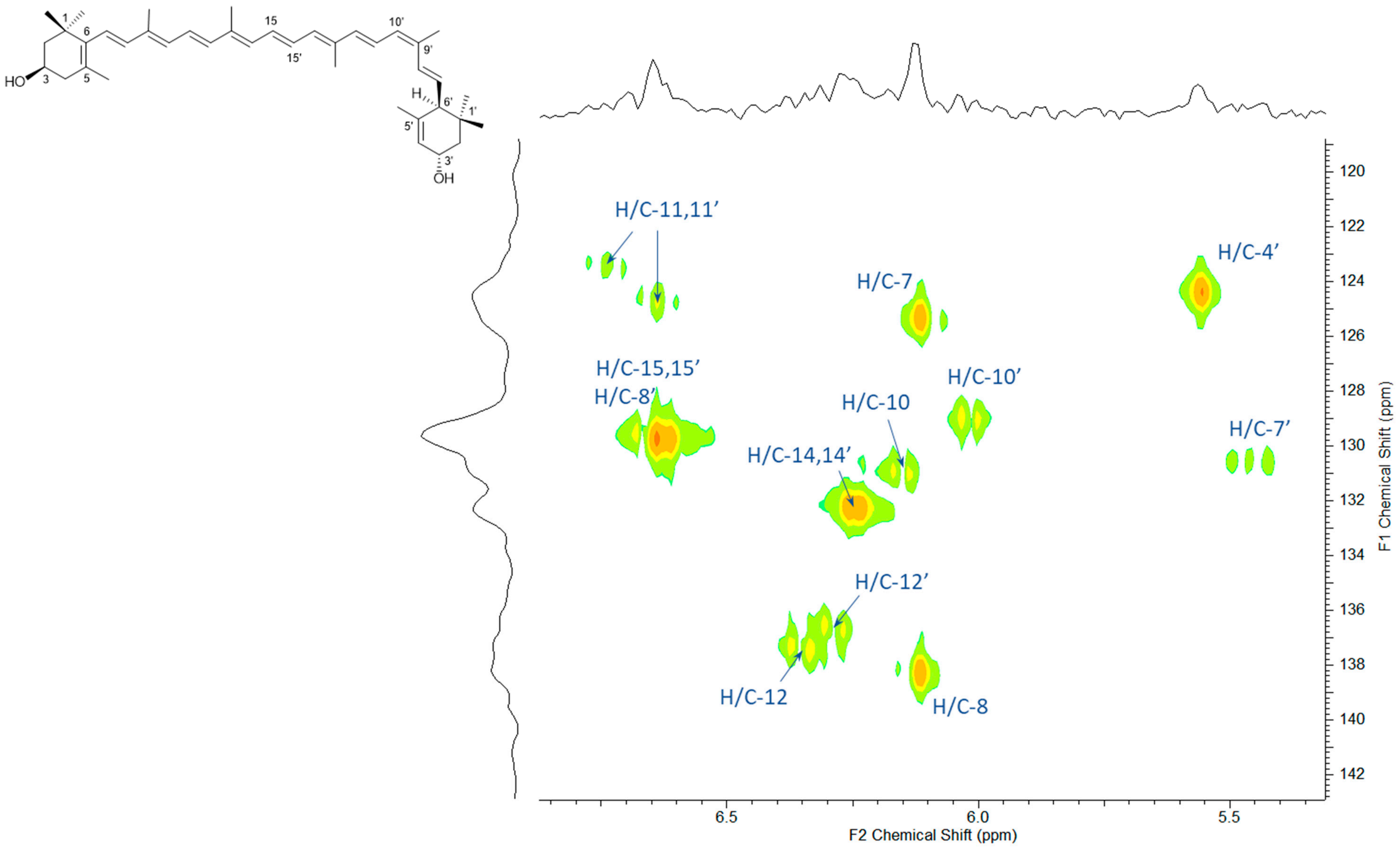

Figure S2.20. ^{13}C-^{1}H-HSQC spectrum of (9′*Z*)-lutein in $CDCl_3$, (500/125 MHz for ^{1}H/^{13}C)

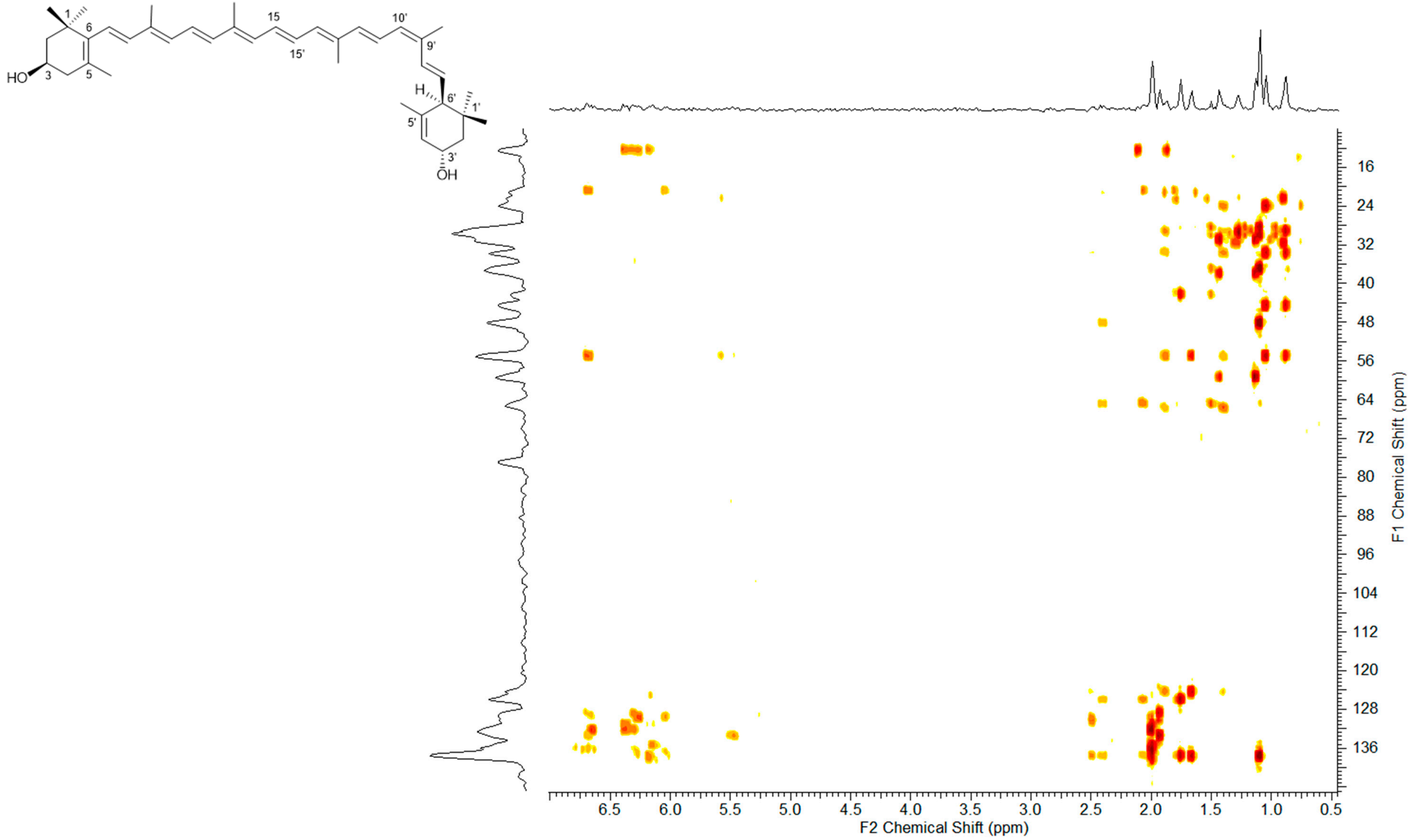

Figure S2.21. ^{13}C-^{1}H-HMBC spectrum of (9′*Z*)-lutein in $CDCl_3$, (500/125 MHz for ^{1}H/^{13}C)

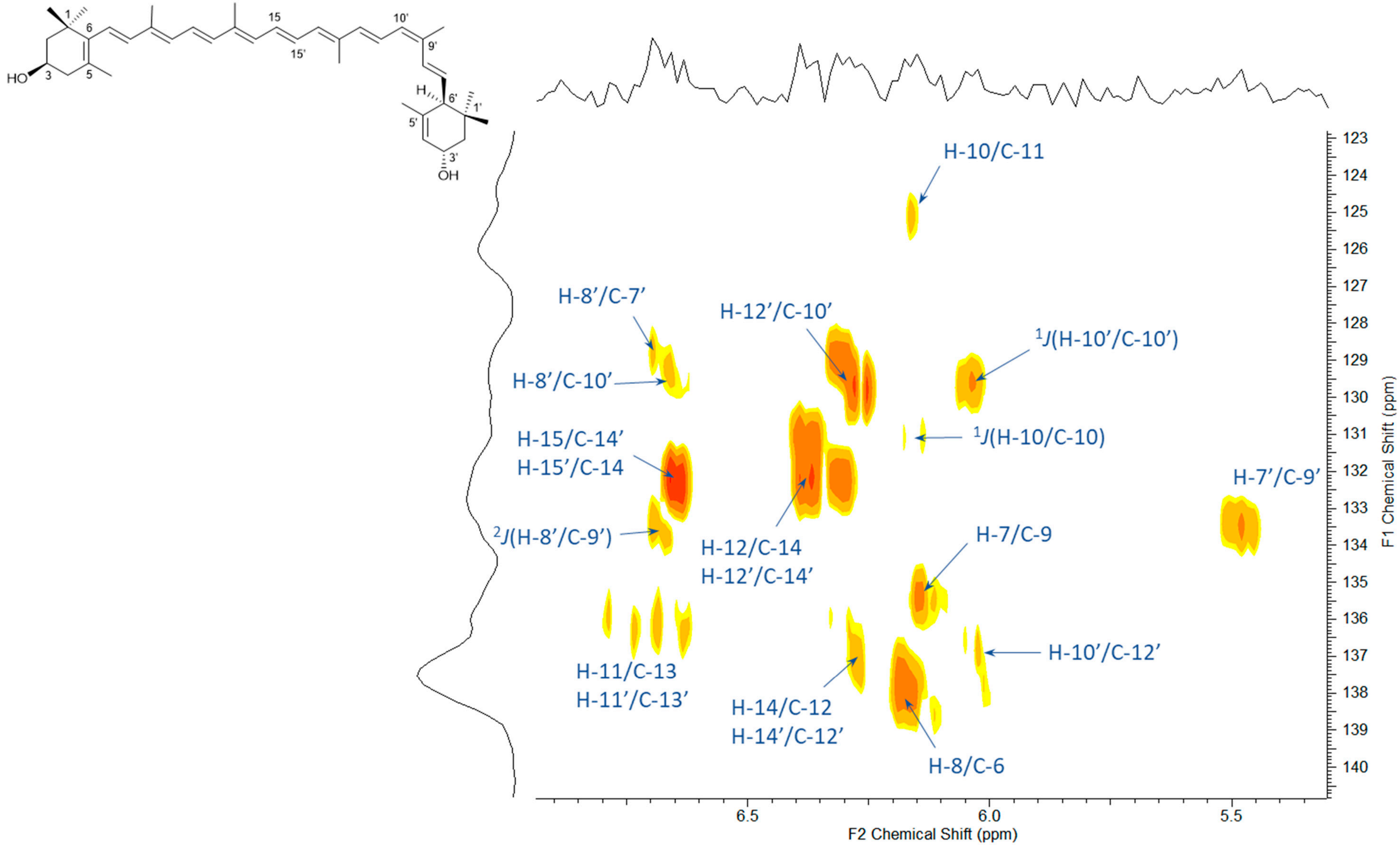

Figure S2.22. ^{13}C-^{1}H-HMBC spectrum of (9′*Z*)-lutein in $CDCl_3$, (500/125 MHz for ^{1}H/^{13}C)

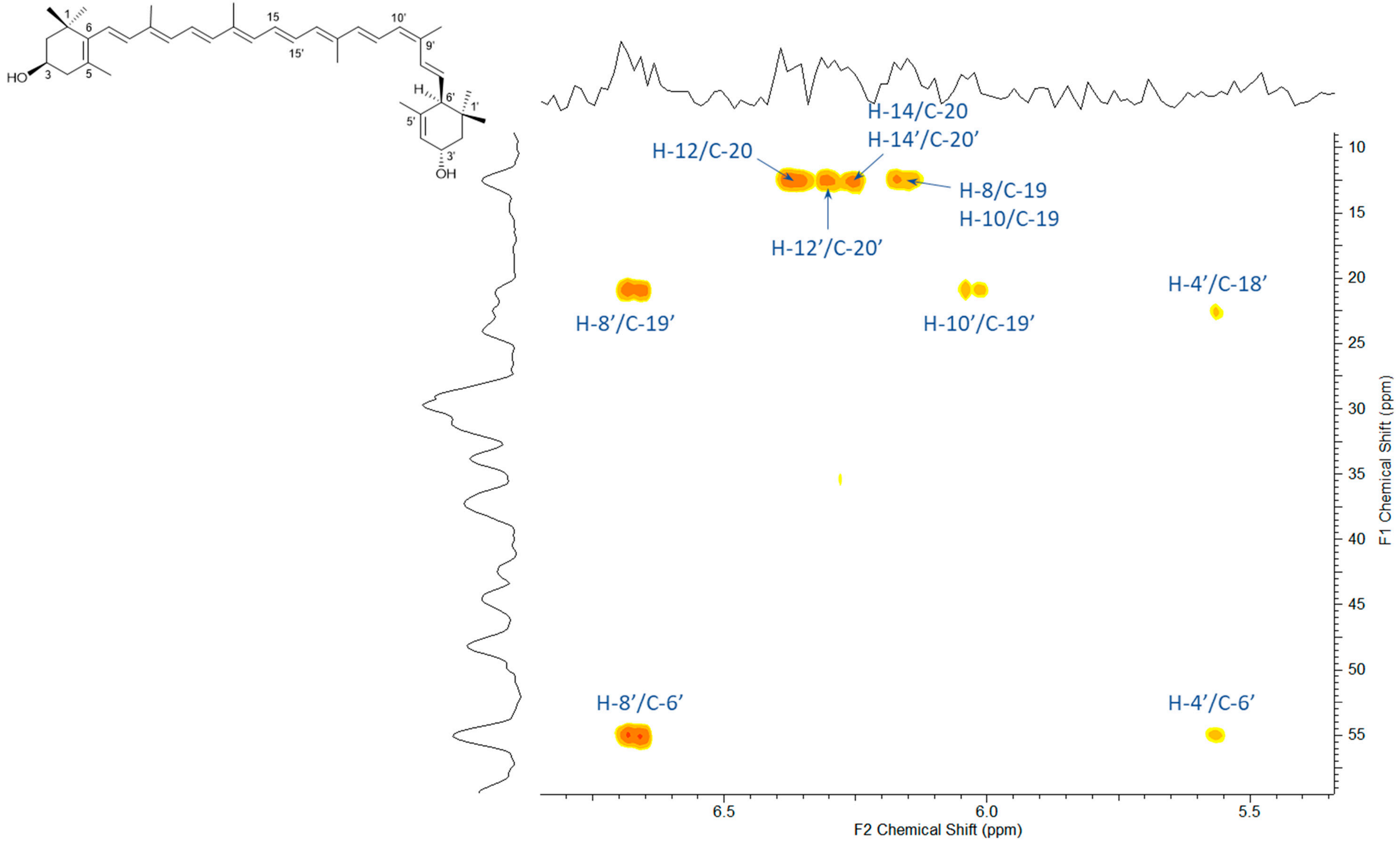

Figure S2.23. ^{13}C-^{1}H-HMBC spectrum of (9′*Z*)-lutein in $CDCl_3$, (500/125 MHz for ^{1}H/^{13}C)

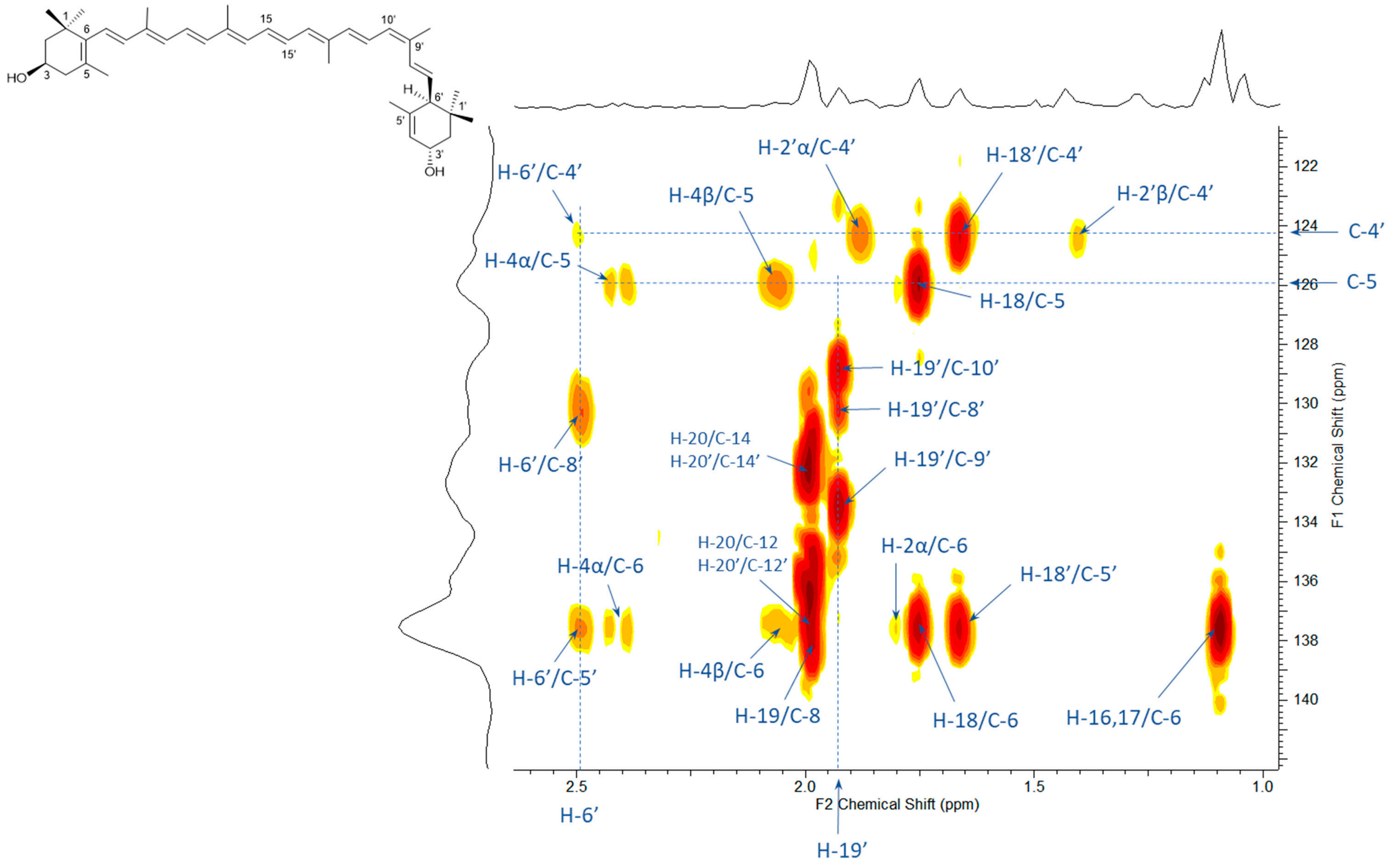

Figure S2.24. ^{13}C-^{1}H-HMBC spectrum of (9′*Z*)-lutein in $CDCl_3$, (500/125 MHz for ^{1}H/^{13}C)

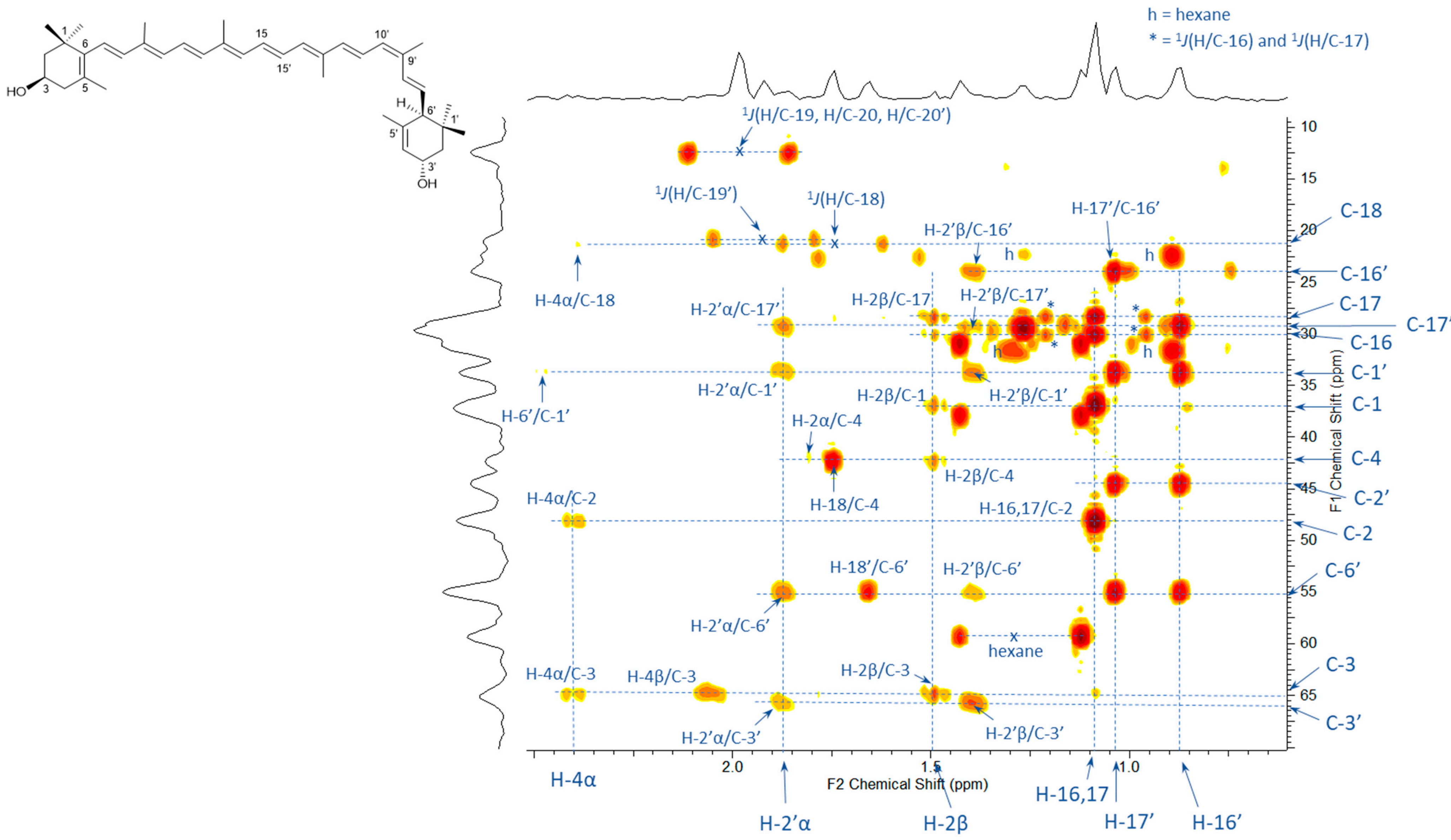

Figure S2.25. ^{13}C-^{1}H-HMBC spectrum of (9′*Z*)-lutein in $CDCl_3$, (500/125 MHz for ^{1}H/^{13}C)

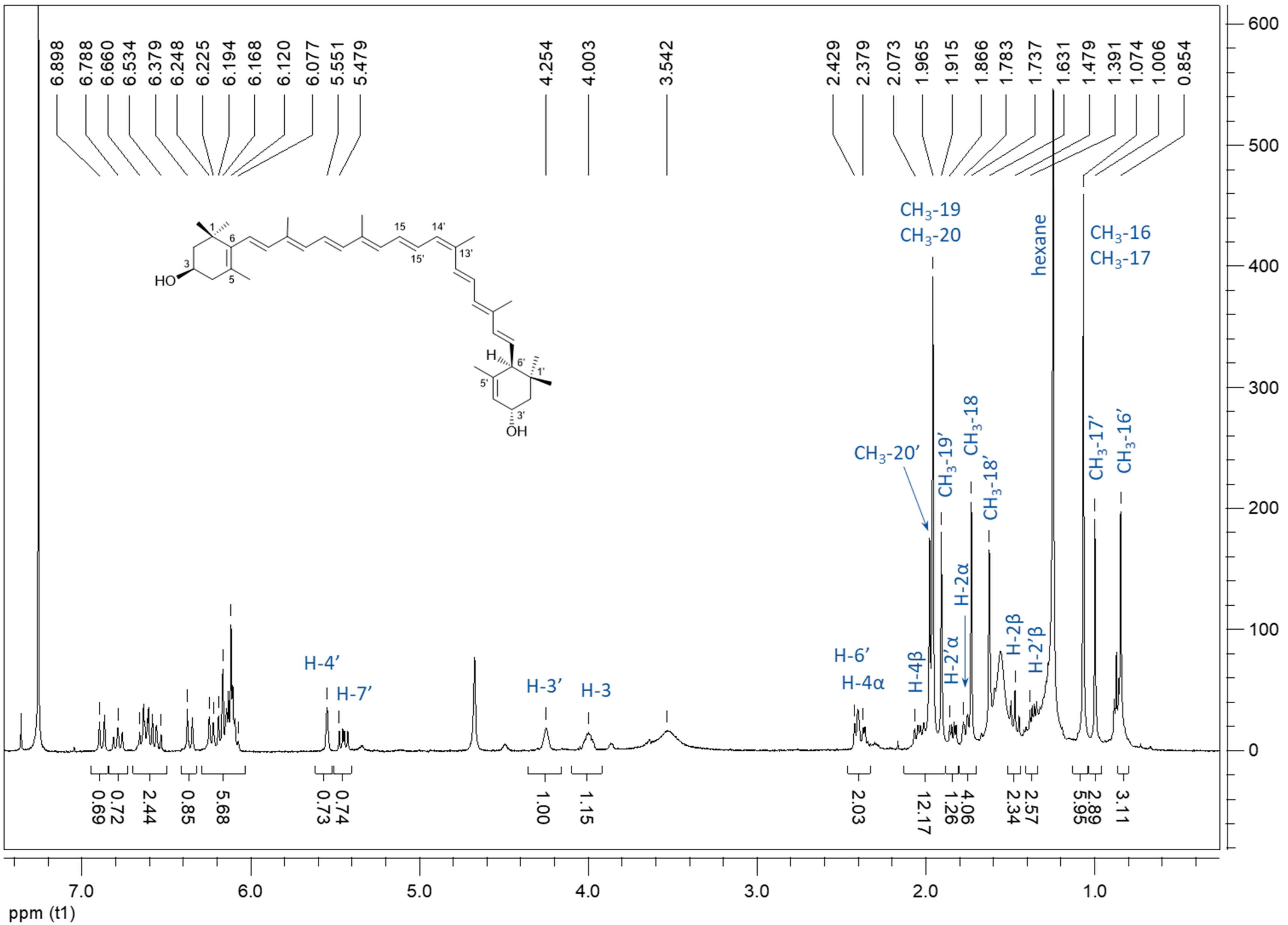

Figure S2.26. ^{1}H-NMR spectrum of (13′Z)-lutein in $CDCl_3$, (500/125 MHz for $^1H/^{13}C$)

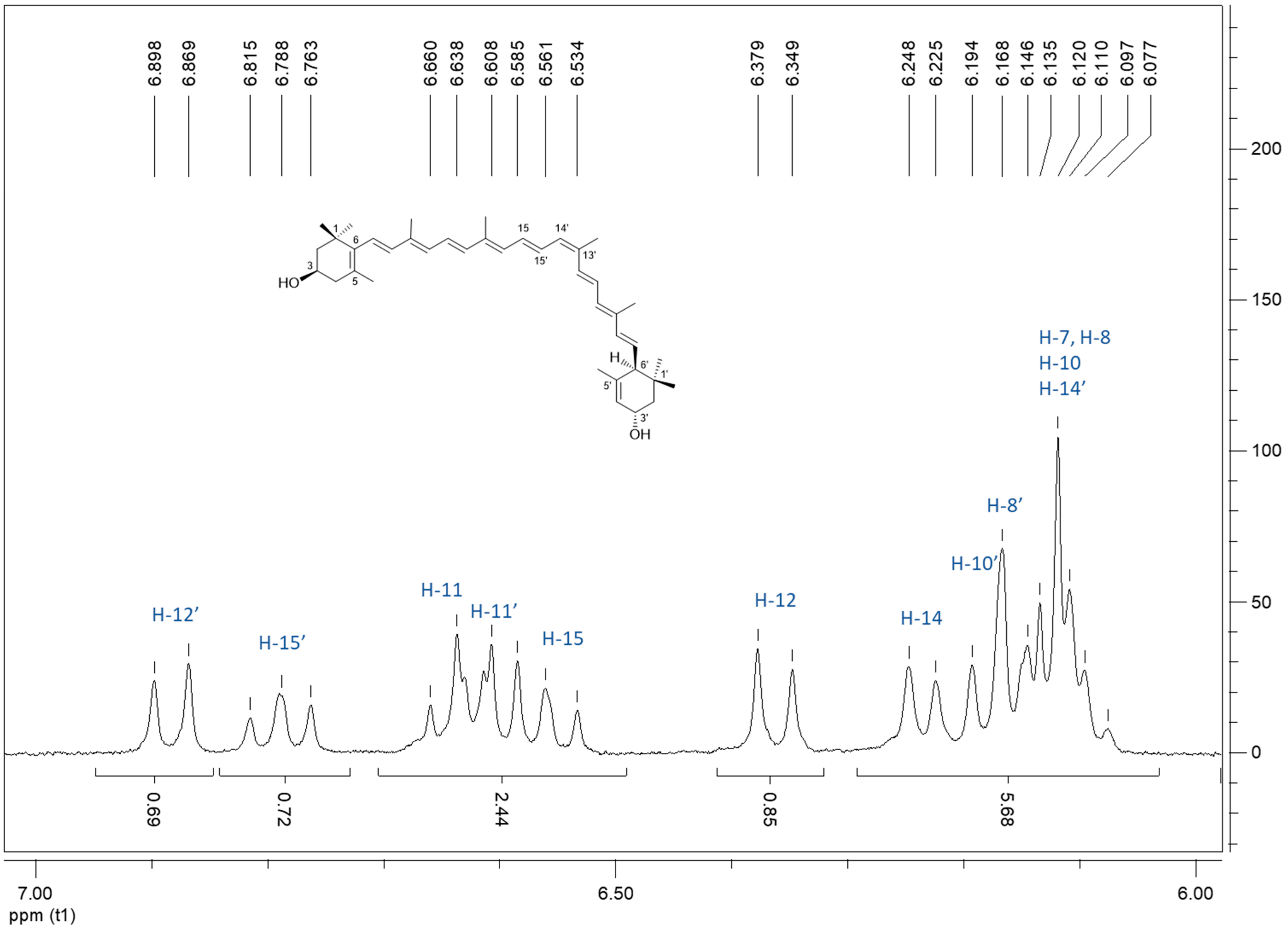

Figure S2.27. ^{1}H-NMR spectrum of (13′*Z*)-lutein in $CDCl_3$, (500/125 MHz for ^{1}H/^{13}C)

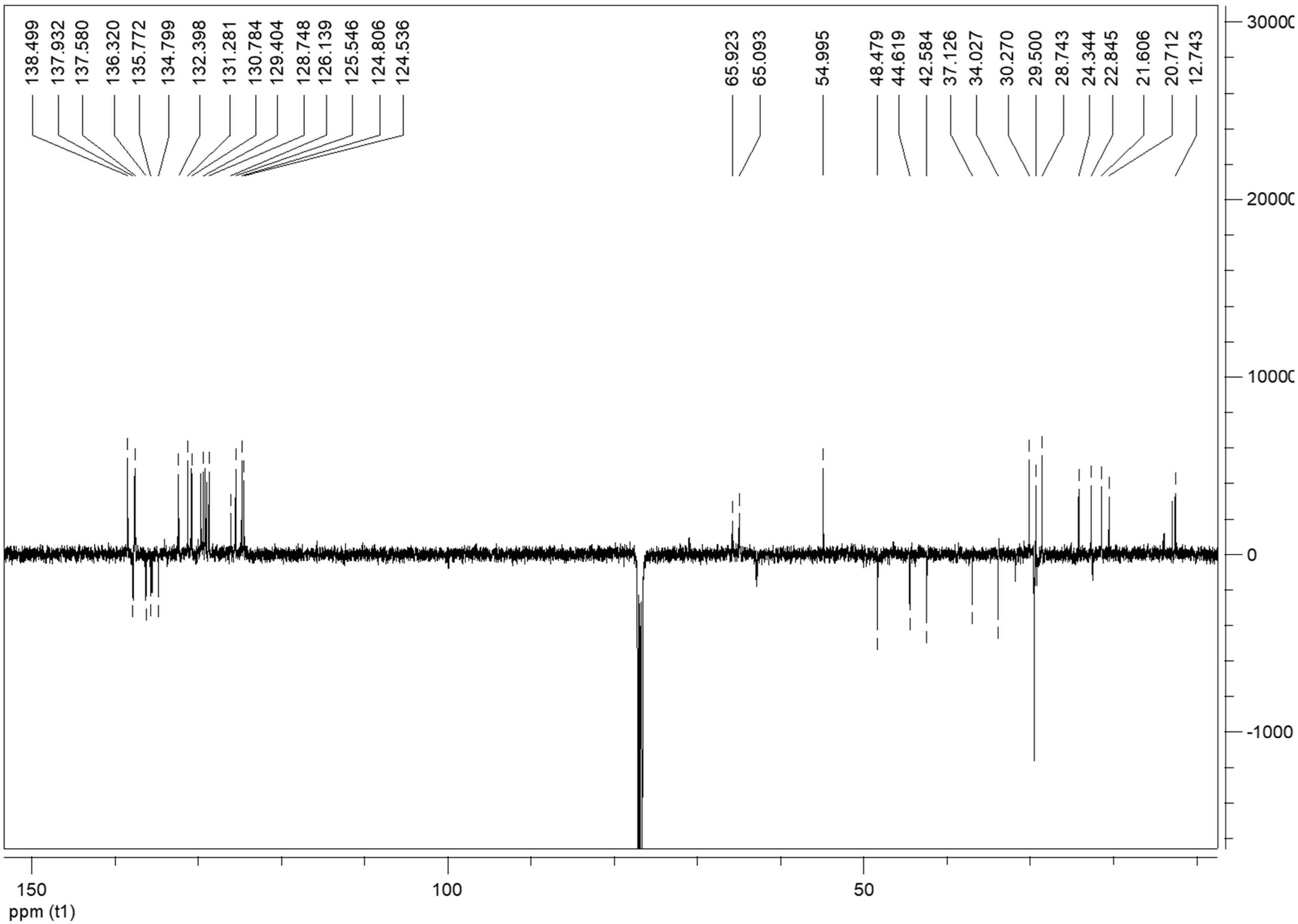

Figure S2.28. ^{13}C-NMR spectrum of (13′Z)-lutein in $CDCl_3$, (500/125 MHz for ^{1}H/^{13}C)

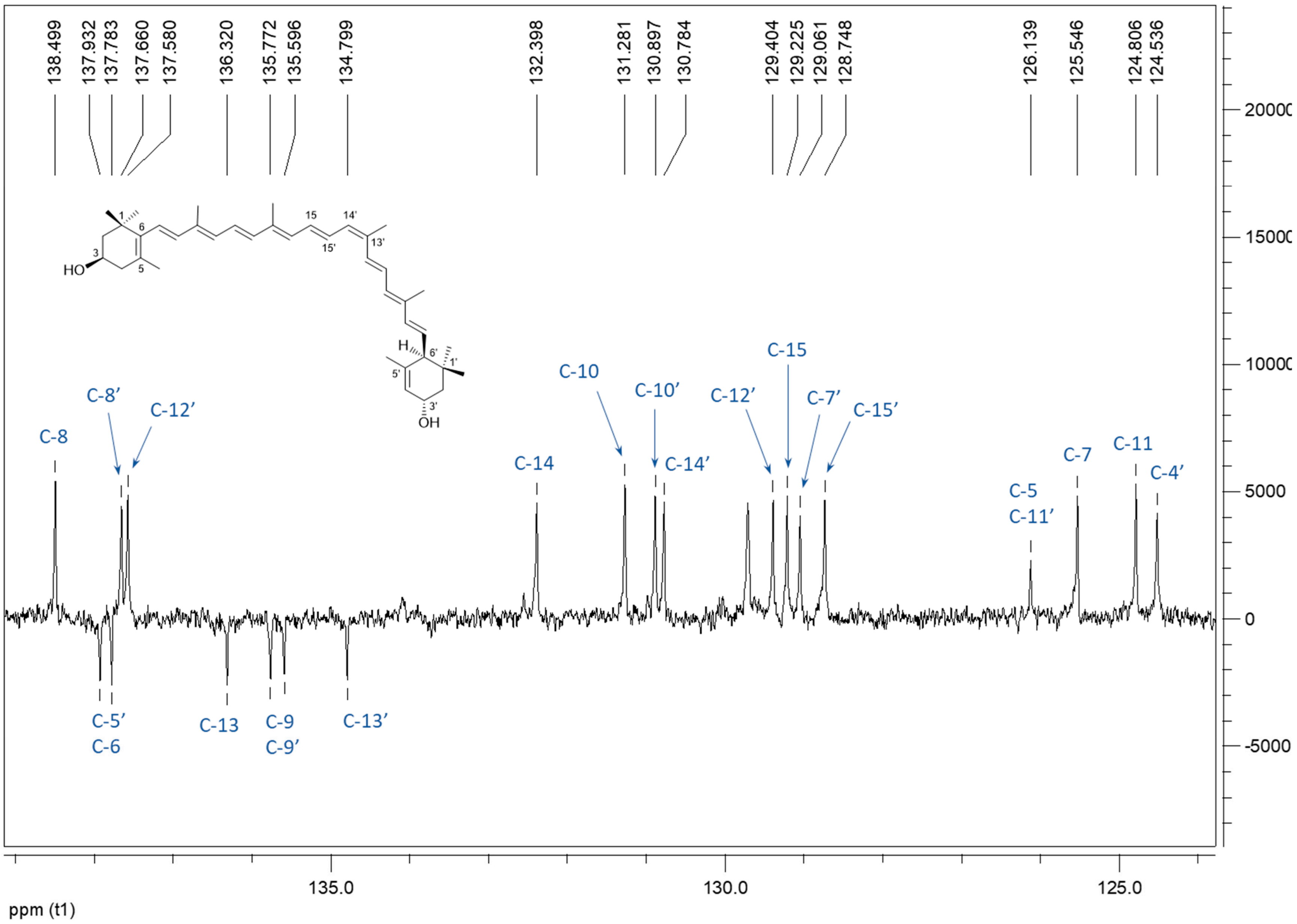

Figure S2.29. ^{13}C-NMR spectrum of (13′Z)-lutein in $CDCl_3$, (500/125 MHz for $^{1}H/^{13}C$)

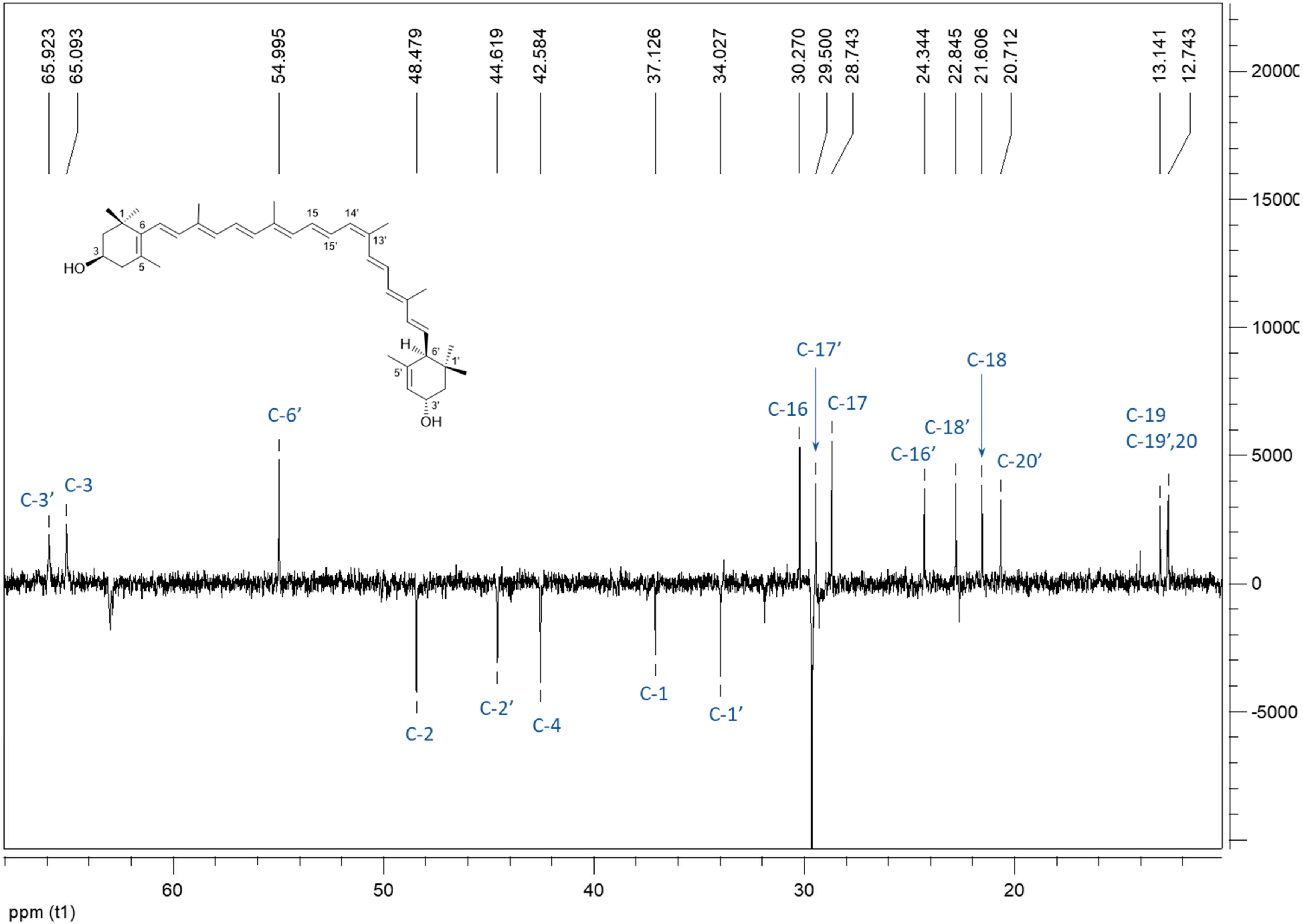

Figure S2.30. ^{13}C-NMR spectrum of (13′Z)-lutein in $CDCl_3$, (500/125 MHz for ^{1}H/^{13}C)

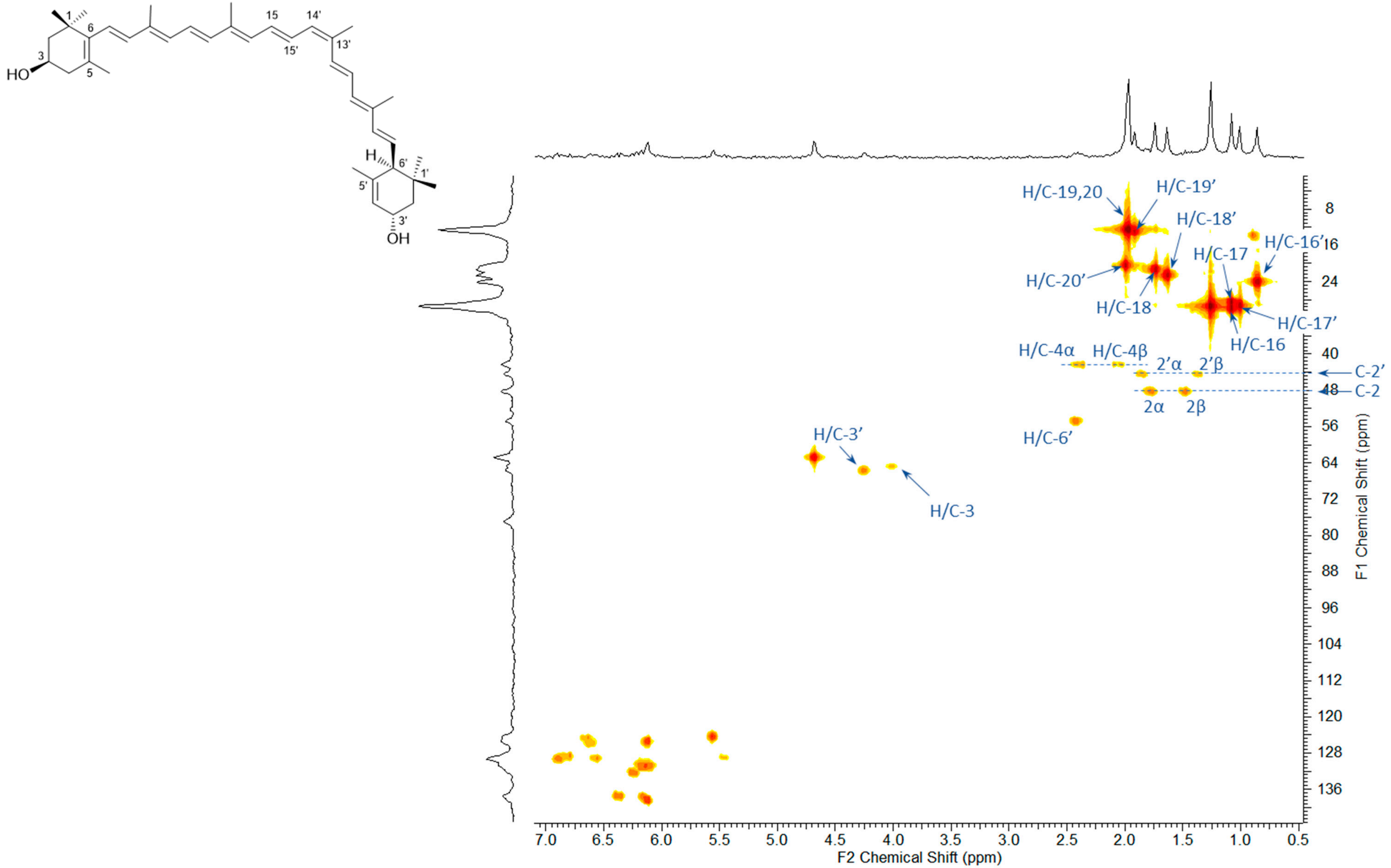

Figure S2.31. ^{13}C-^{1}H-HSQC spectrum of (13′*Z*)-lutein in $CDCl_3$, (500/125 MHz for ^{1}H/^{13}C)

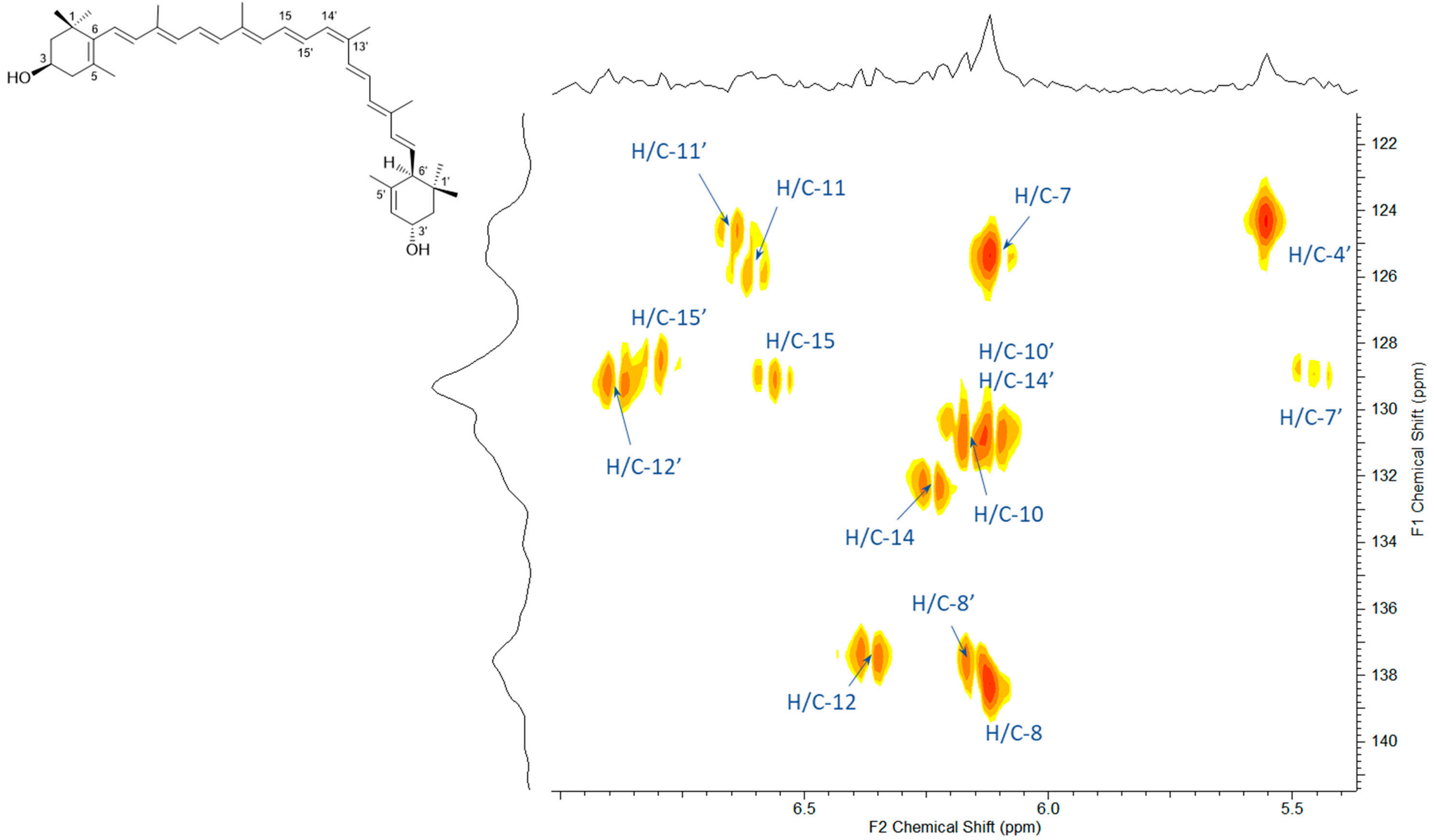

Figure S2.32. ^{13}C-^{1}H-HSQC spectrum of (13′*Z*)-lutein in CDCl$_3$, (500/125 MHz for ^{1}H/^{13}C)

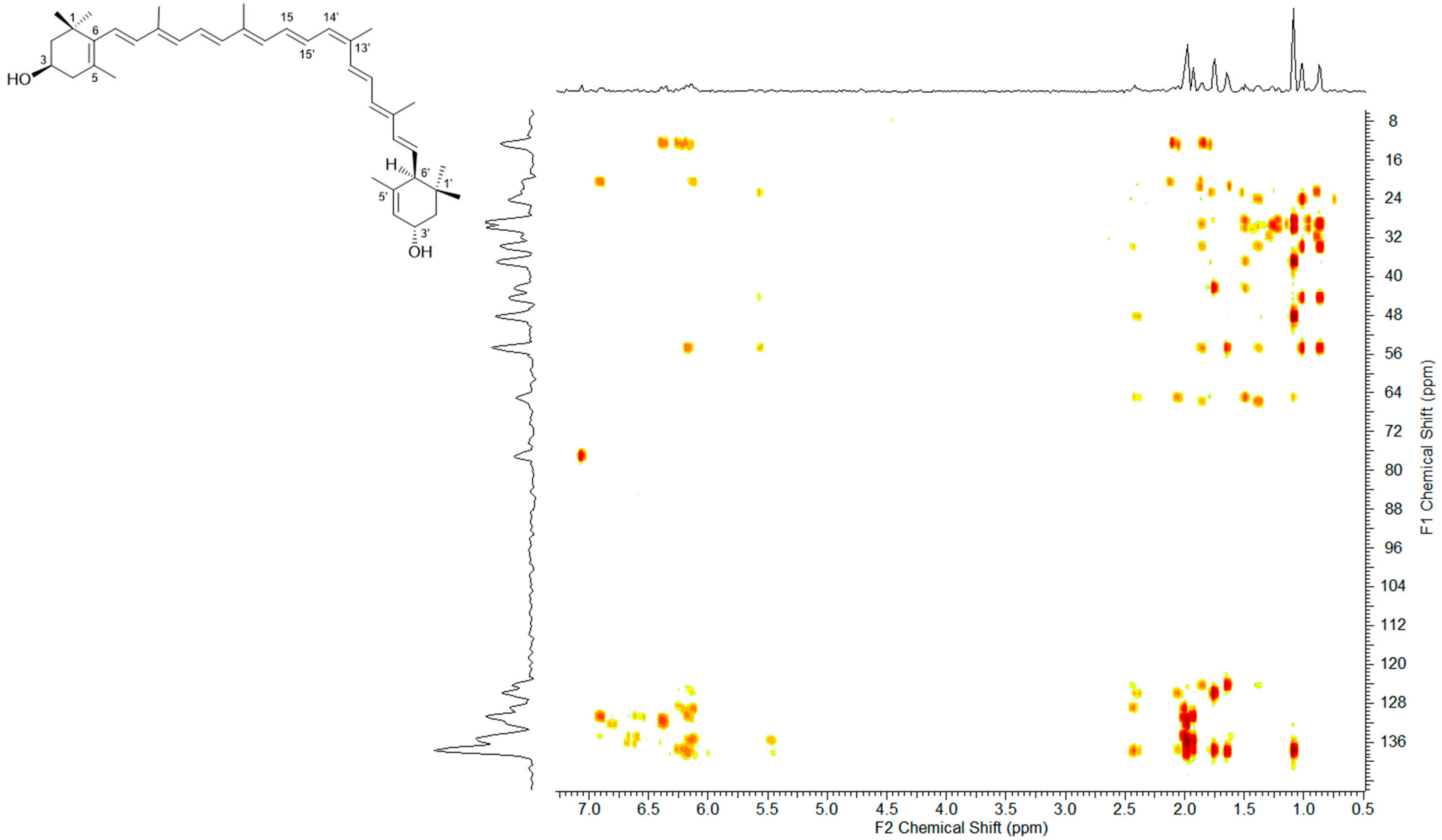

Figure S2.33. ^{13}C-^{1}H-HMBC spectrum of (13′*Z*)-lutein in $CDCl_3$, (500/125 MHz for ^{1}H/^{13}C)

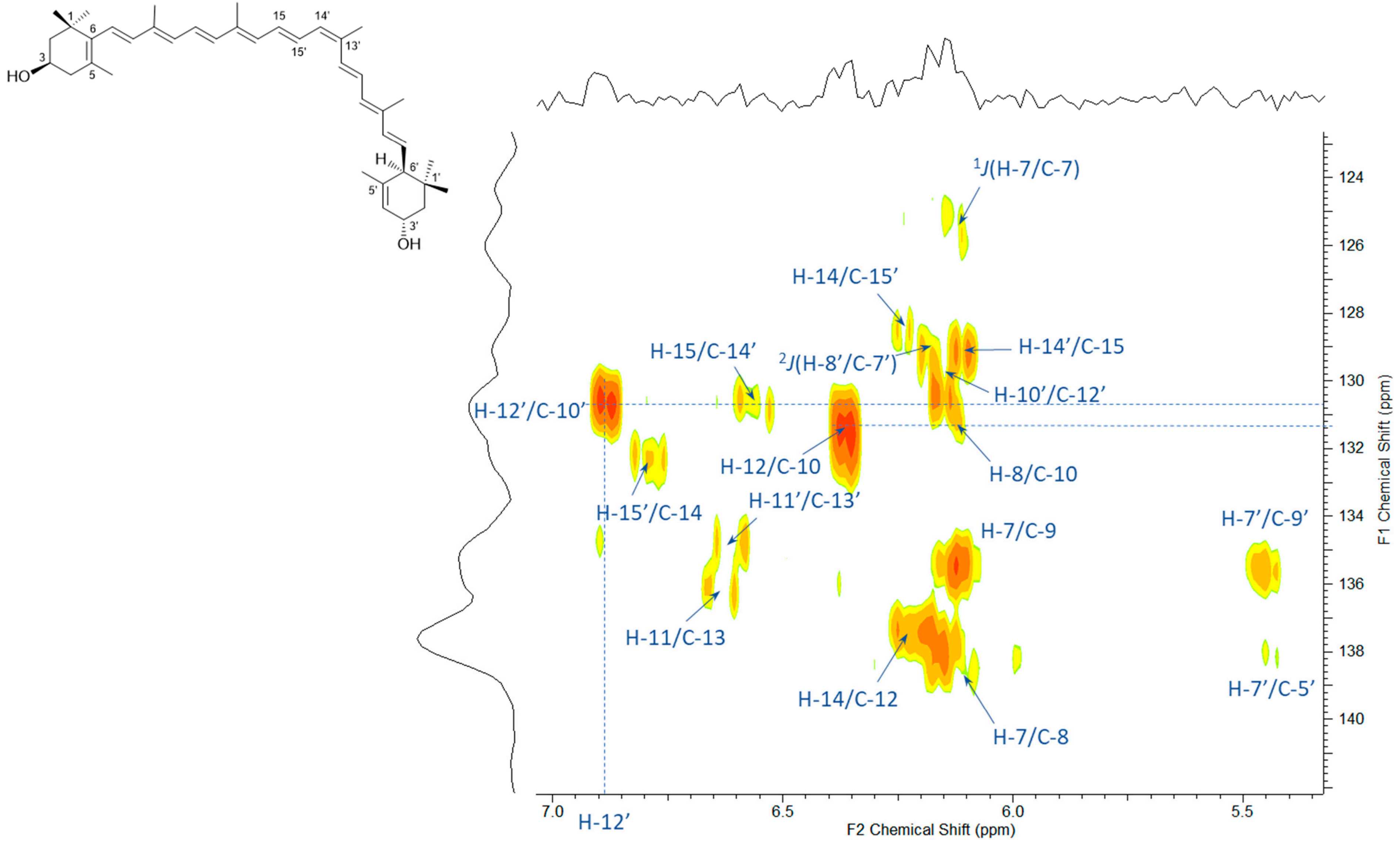

Figure S2.34. ^{13}C-^{1}H-HMBC spectrum of (13′Z)-lutein in $CDCl_3$, (500/125 MHz for ^{1}H/^{13}C)

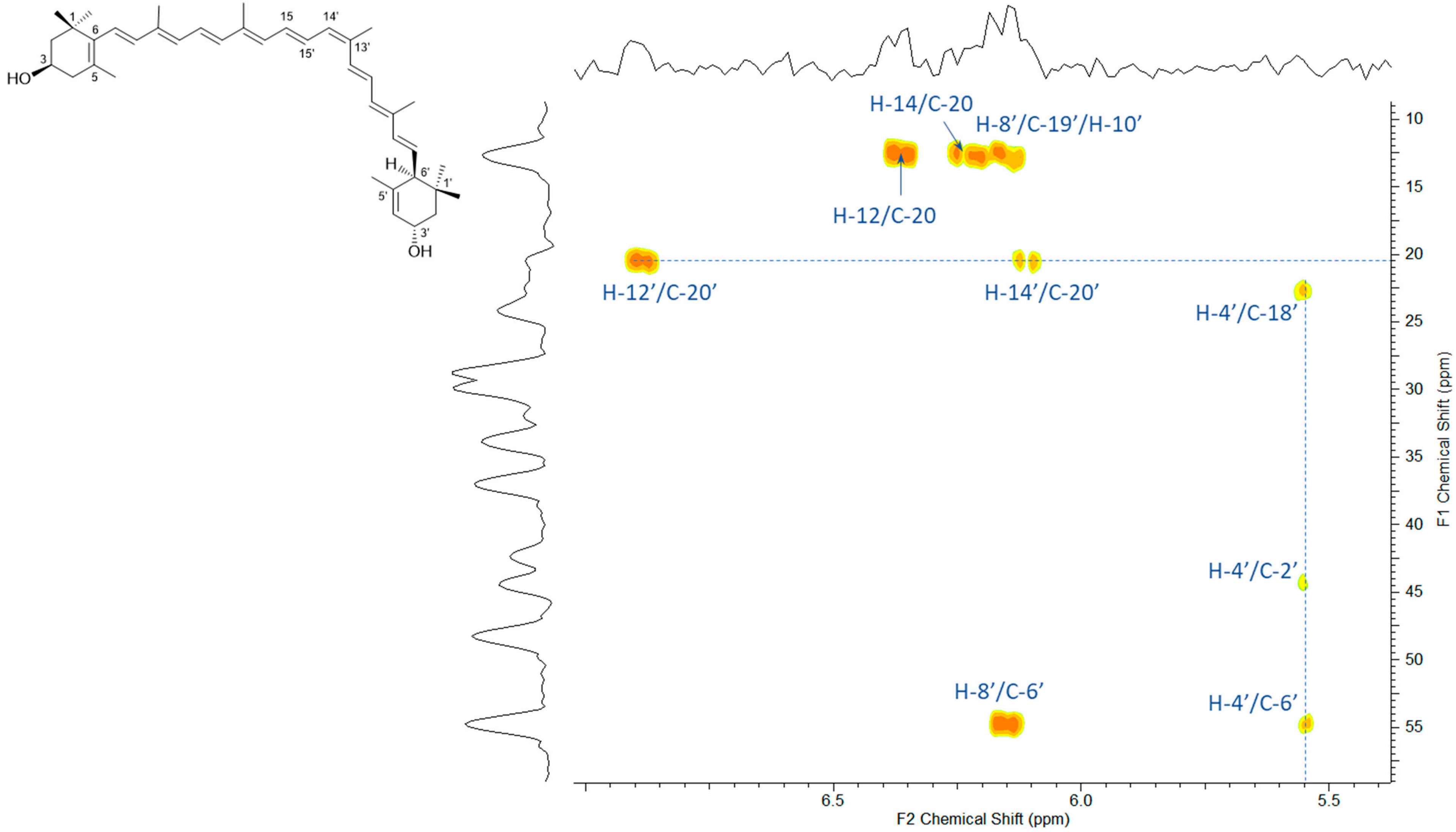

Figure S2.35. ^{13}C-^{1}H-HMBC spectrum of (13′*Z*)-lutein in $CDCl_3$, (500/125 MHz for ^{1}H/^{13}C)

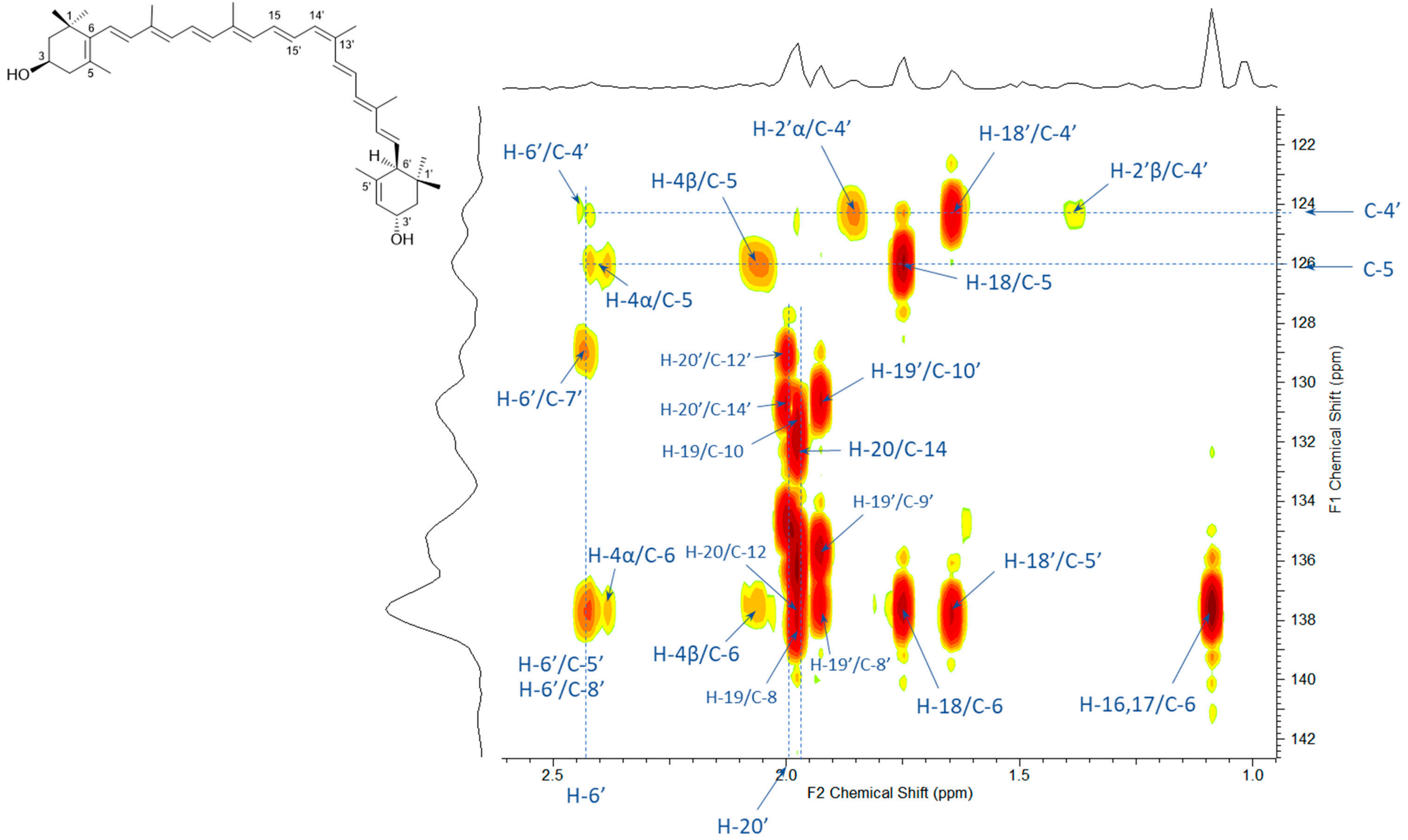

Figure S2.36. ^{13}C-^{1}H-HMBC spectrum of (13′*Z*)-lutein in $CDCl_3$, (500/125 MHz for ^{1}H/^{13}C)

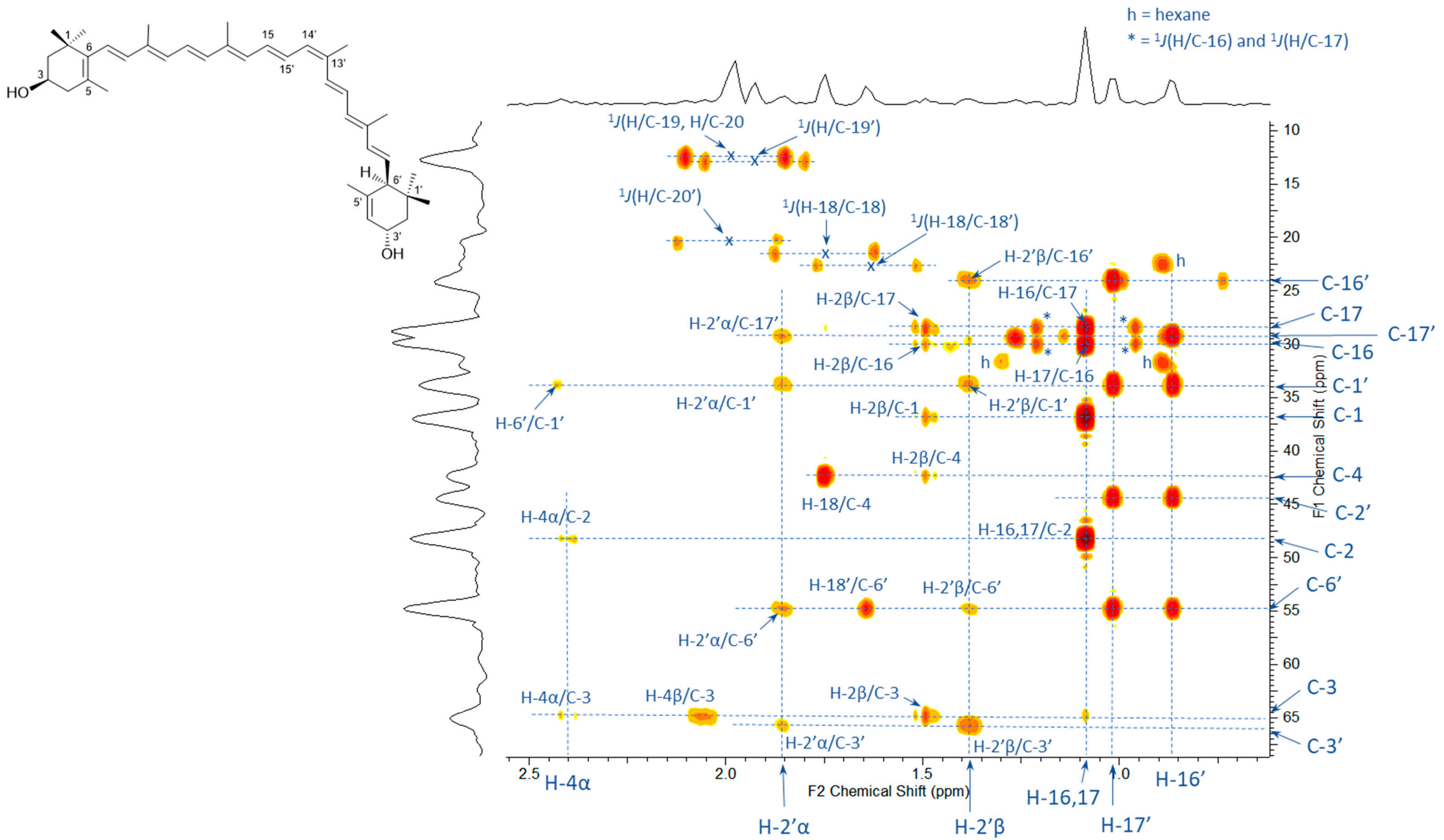

Figure S2.36. ^{13}C-^{1}H-HMBC spectrum of (13′*Z*)-lutein in $CDCl_3$, (500/125 MHz for ^{1}H/^{13}C)

Figure S3. UV-vis spectra of lutein isomers, in the HPLC mobile phase

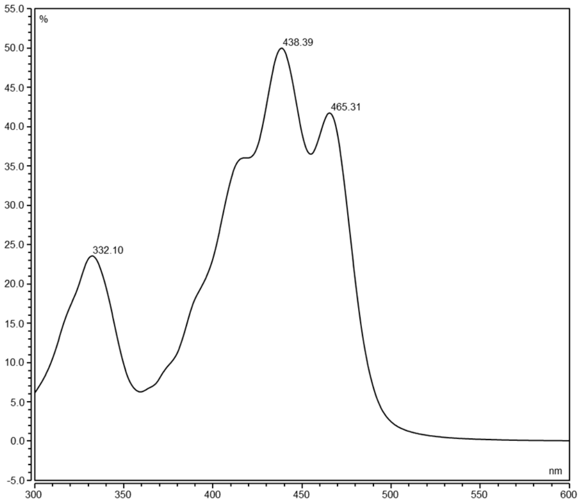

Figure S3.1. UV-vis spectrum of (13Z)-lutein in the HPLC mobile phase at 11.9 min. Q: 2.10, A_B/A_{II}: 47.2%

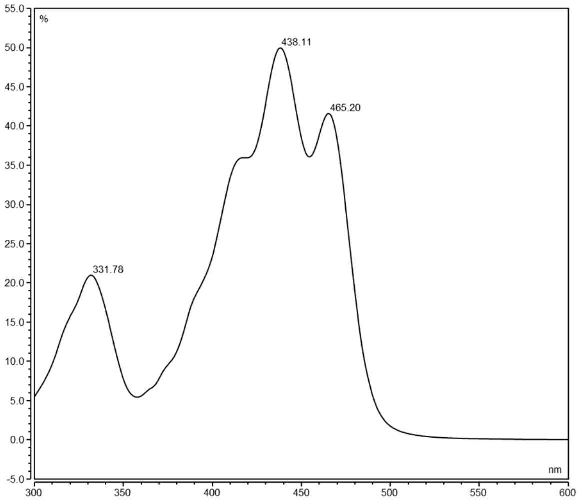

Figure S3.2. UV-vis spectrum of (13’Z)-lutein in the HPLC mobile phase at 12.4 min. Q: 2.37, A_B/A_{II}: 41.7%

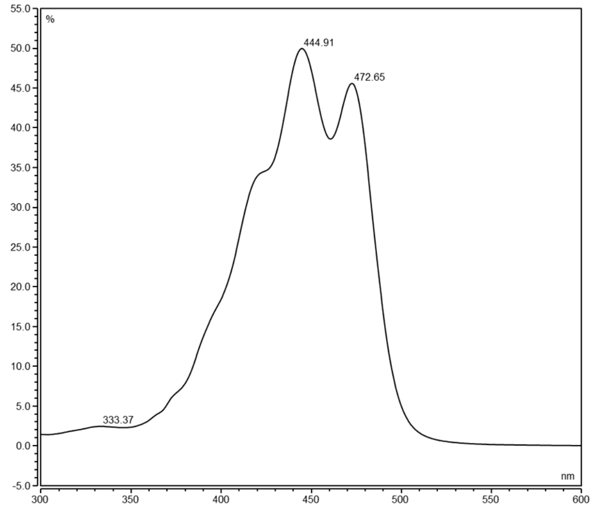

Figure S3.3. UV-vis spectrum of all-(*E*)-lutein in the HPLC mobile phase at 13.3 min.

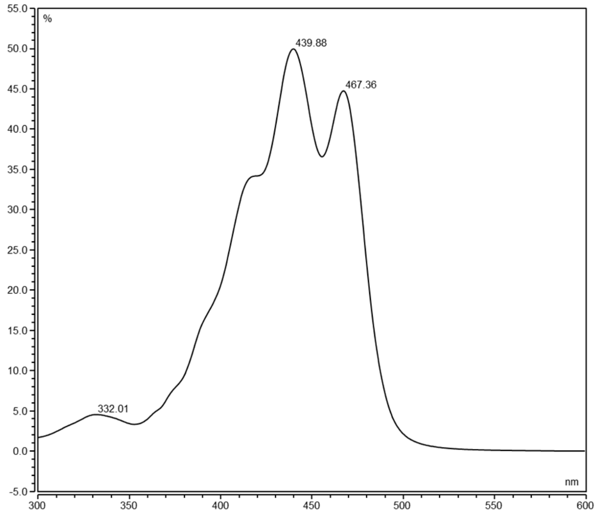

Figure S3.4. UV-vis spectrum of (9*Z*)-lutein in the HPLC mobile phase at 16.8 min. Q: 10.80, A_B/A_{II}: 9.7%

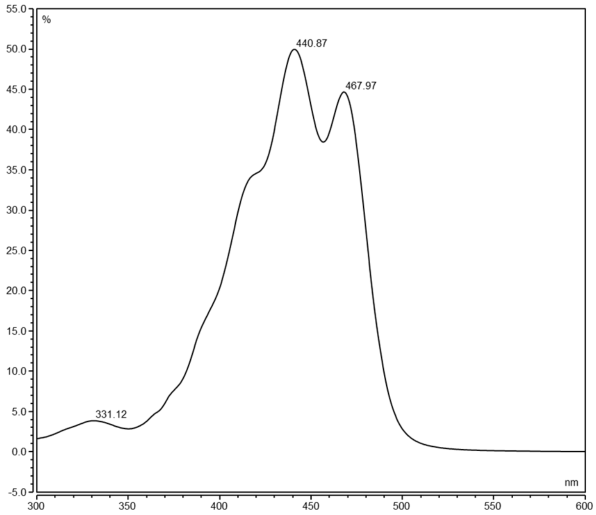

Figure S3.5. UV-vis spectrum of (9′*Z*)-lutein in the HPLC mobile phase at 19.6 min. Q: 13.56, A_B/A_{II}: 8.5%

Figure S4. UV-vis and EIC chromatograms of the investigated flowers

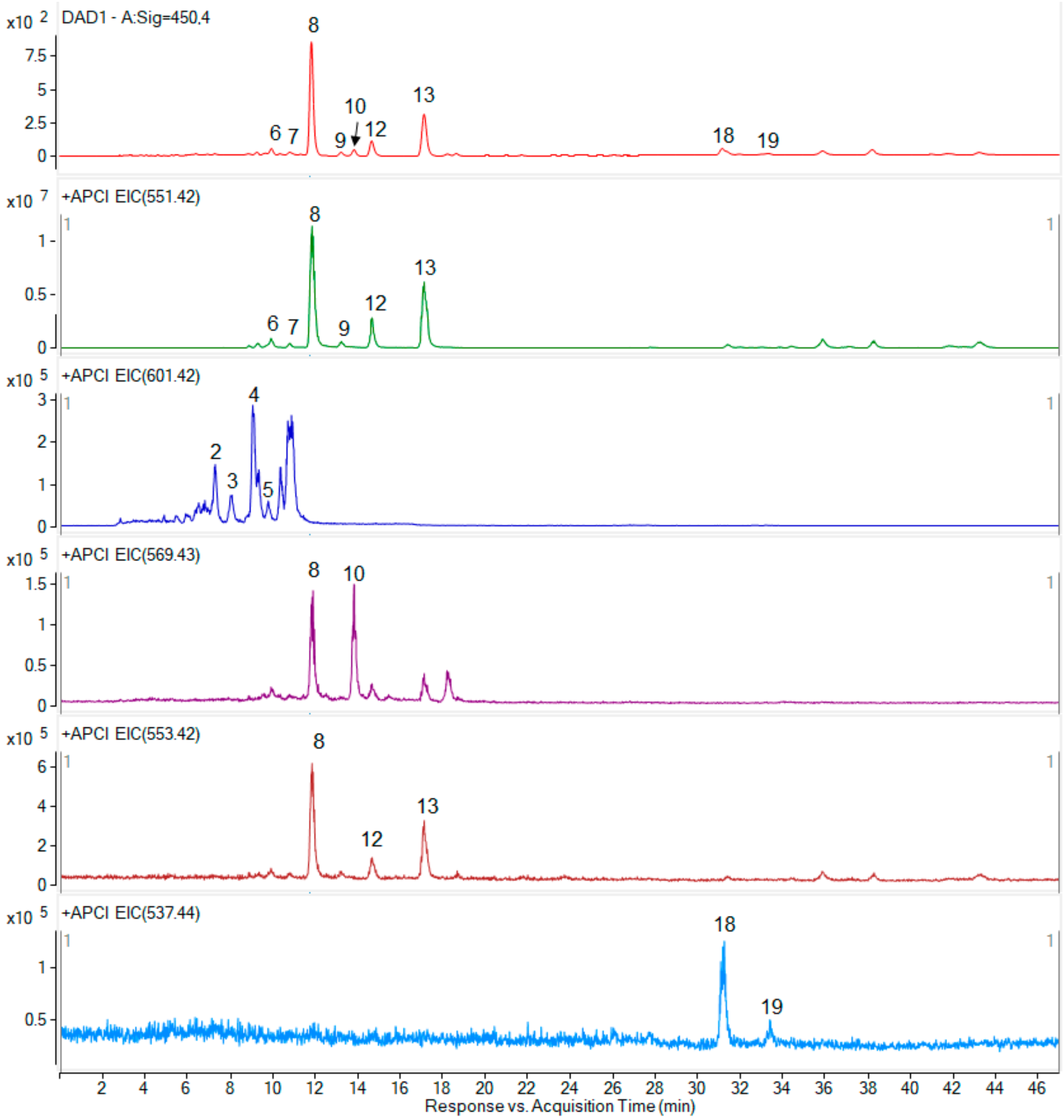

Figure S4.1. UV-vis and EIC chromatogram of *Anthemis tinctoria*

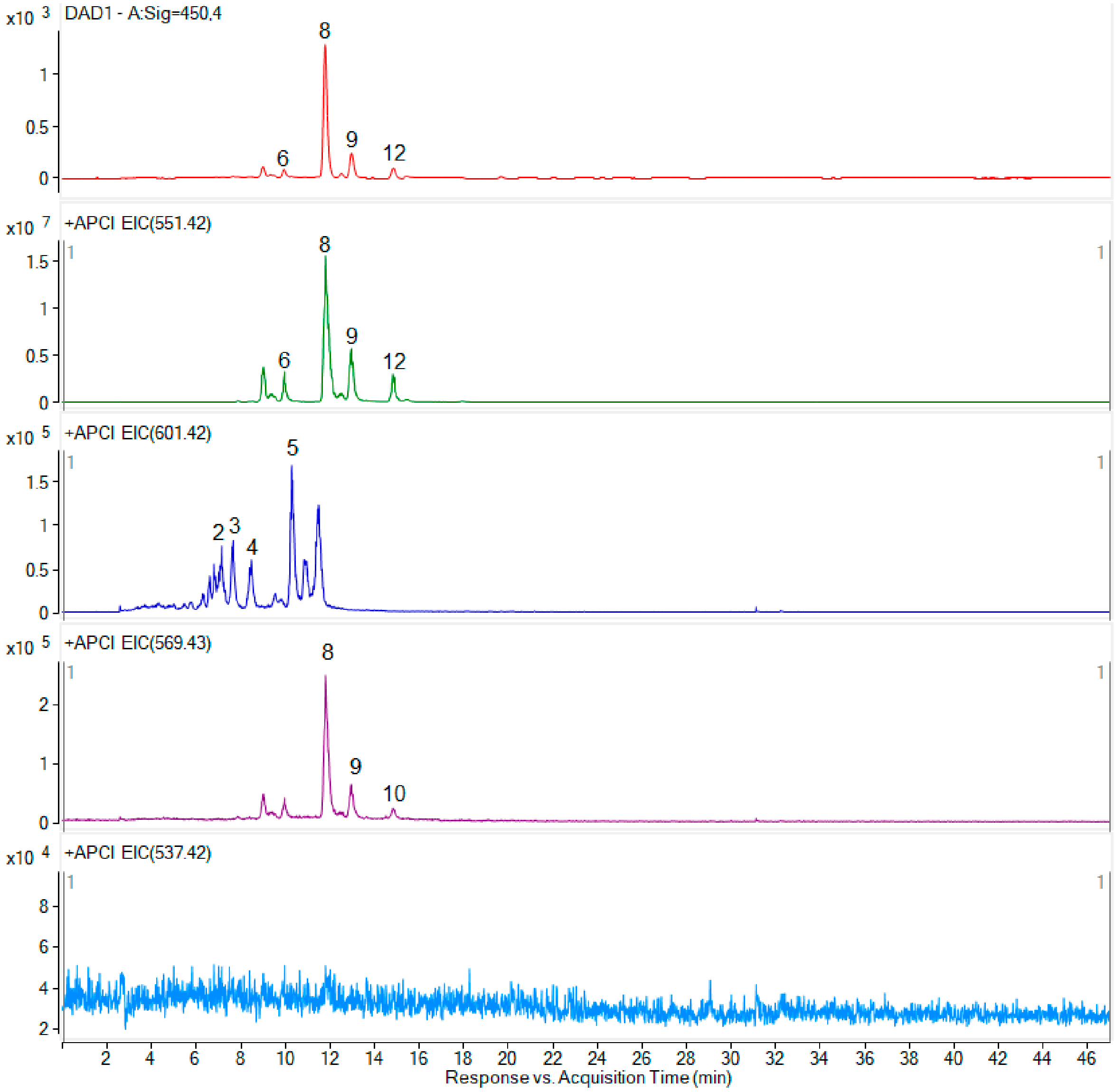

Figure S4.2. UV-vis and EIC chromatogram of *Cholcicum autumnale*

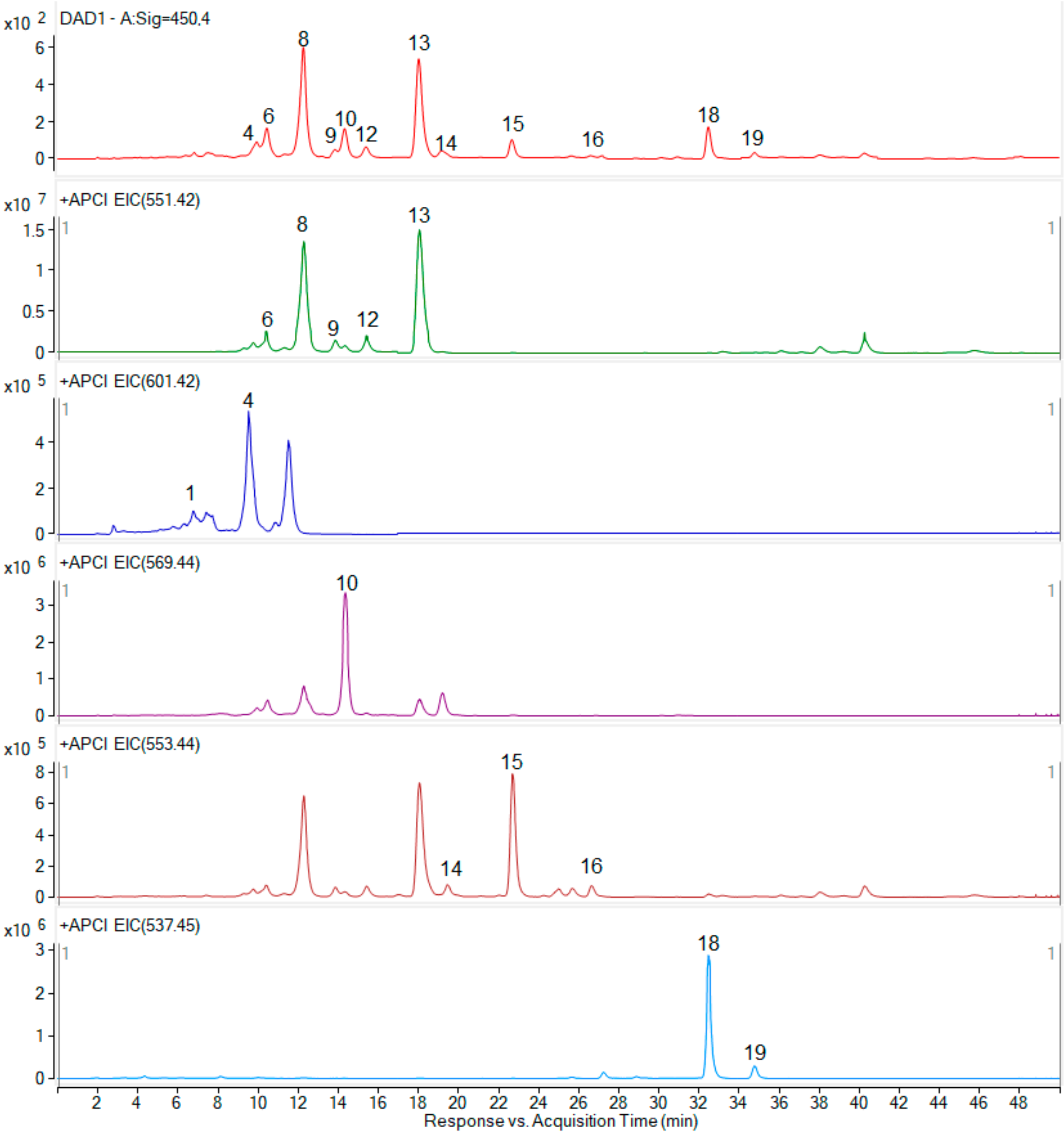

Figure S4.3. UV-vis and EIC chromatogram of *Helicrysum italicum*

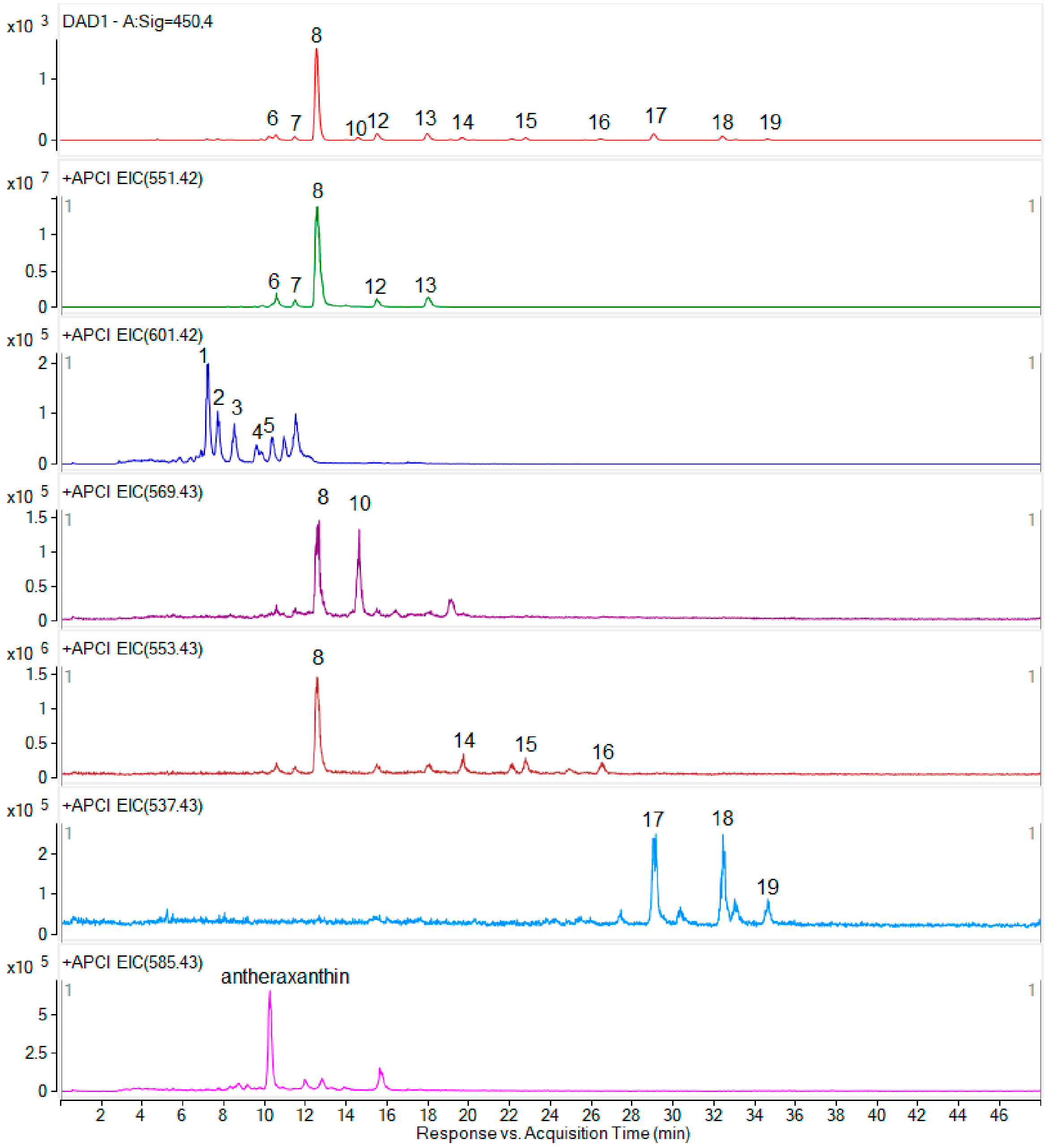

Figure S4.4. UV-vis and EIC chromatogram of *Helianthus angustifolius*

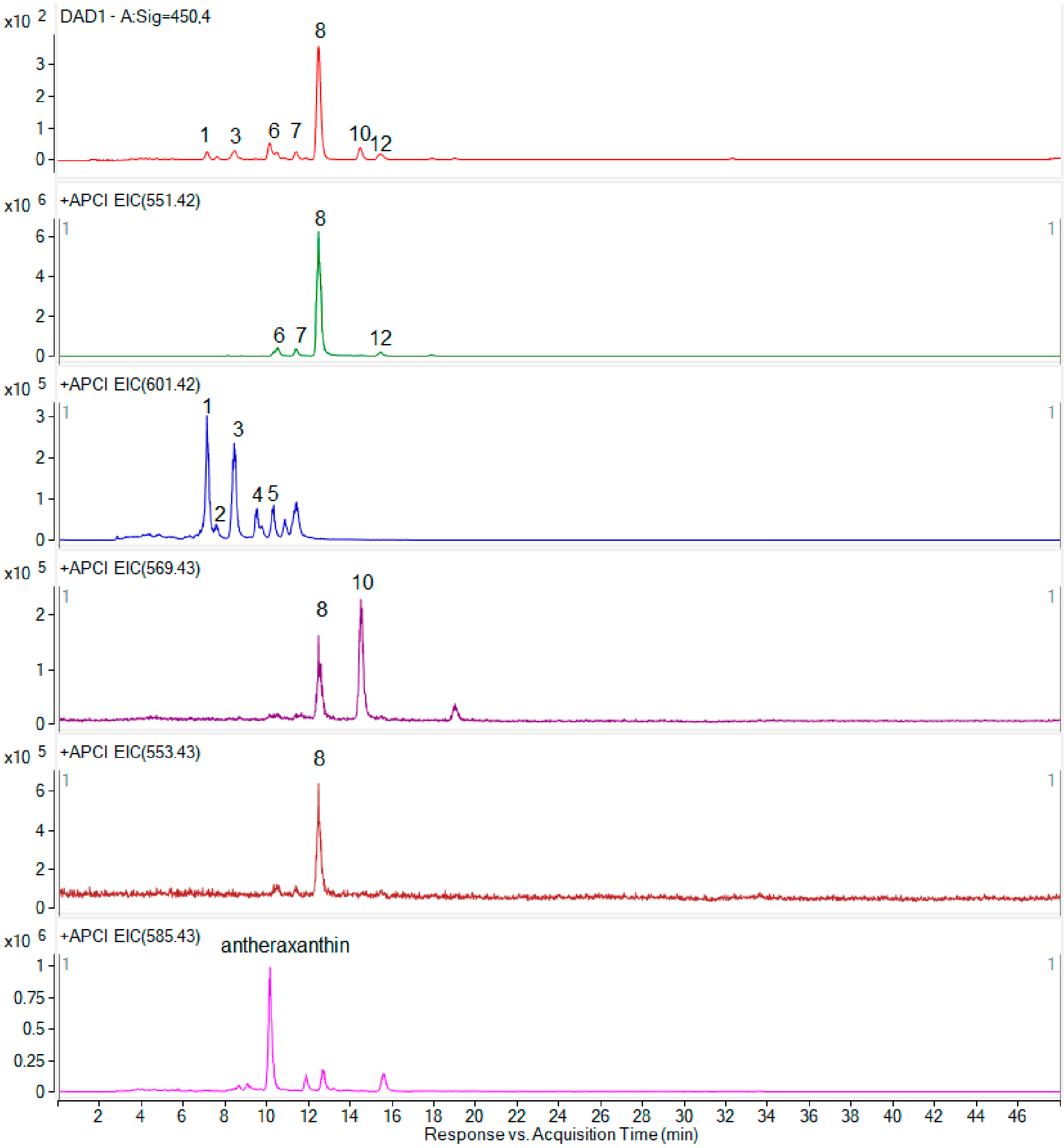

Figure S4.5. UV-vis and EIC chromatogram of *Helianthus tuberosus*

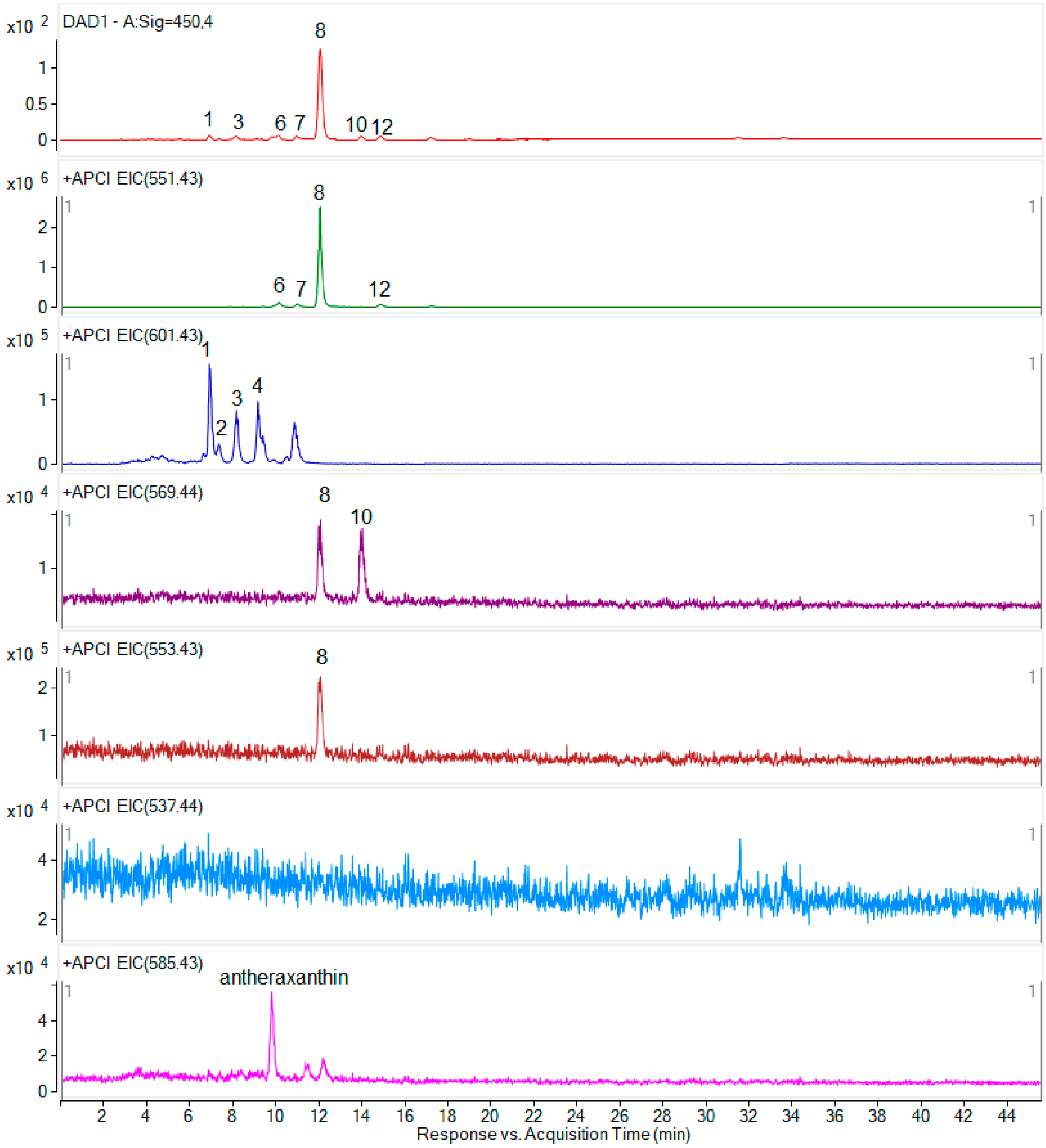

Figure S4.6. UV-vis and EIC chromatogram of *Sternbergia lutea*

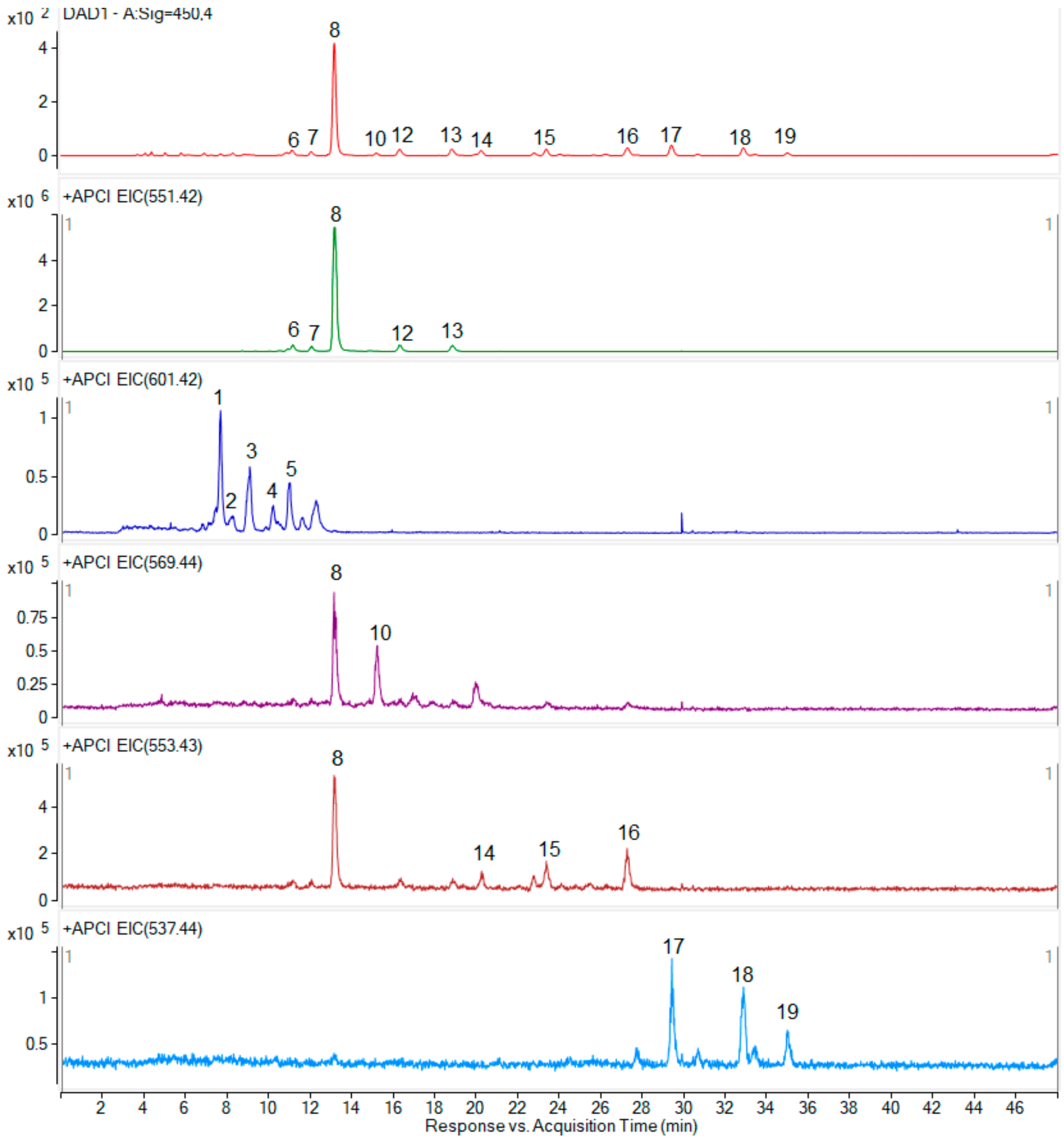

Figure S4.7. UV-vis and EIC chromatogram of *Coreopsis pubescens*

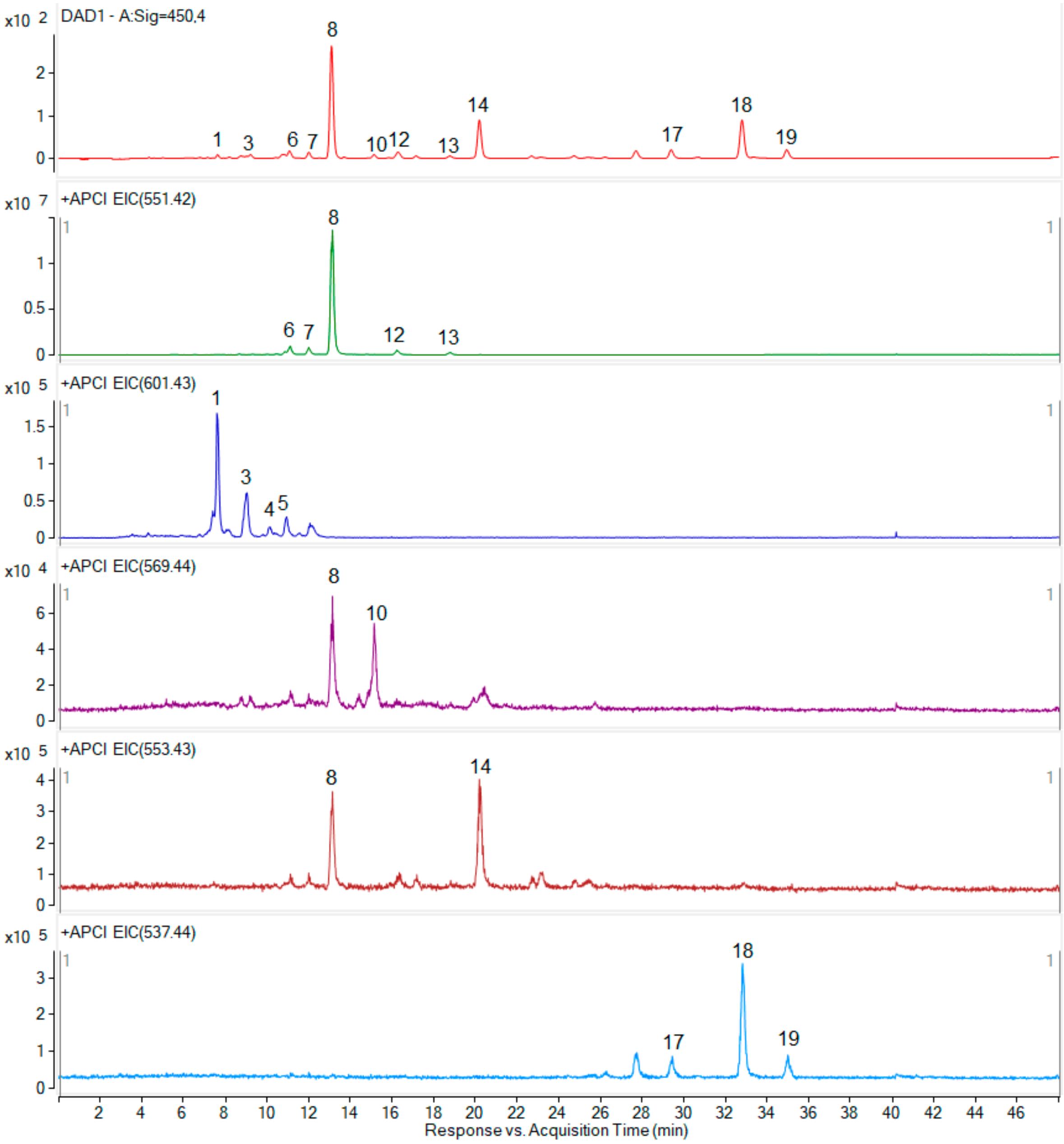

Figure S4.8. UV-vis and EIC chromatogram of *Cassia artemisiodes*

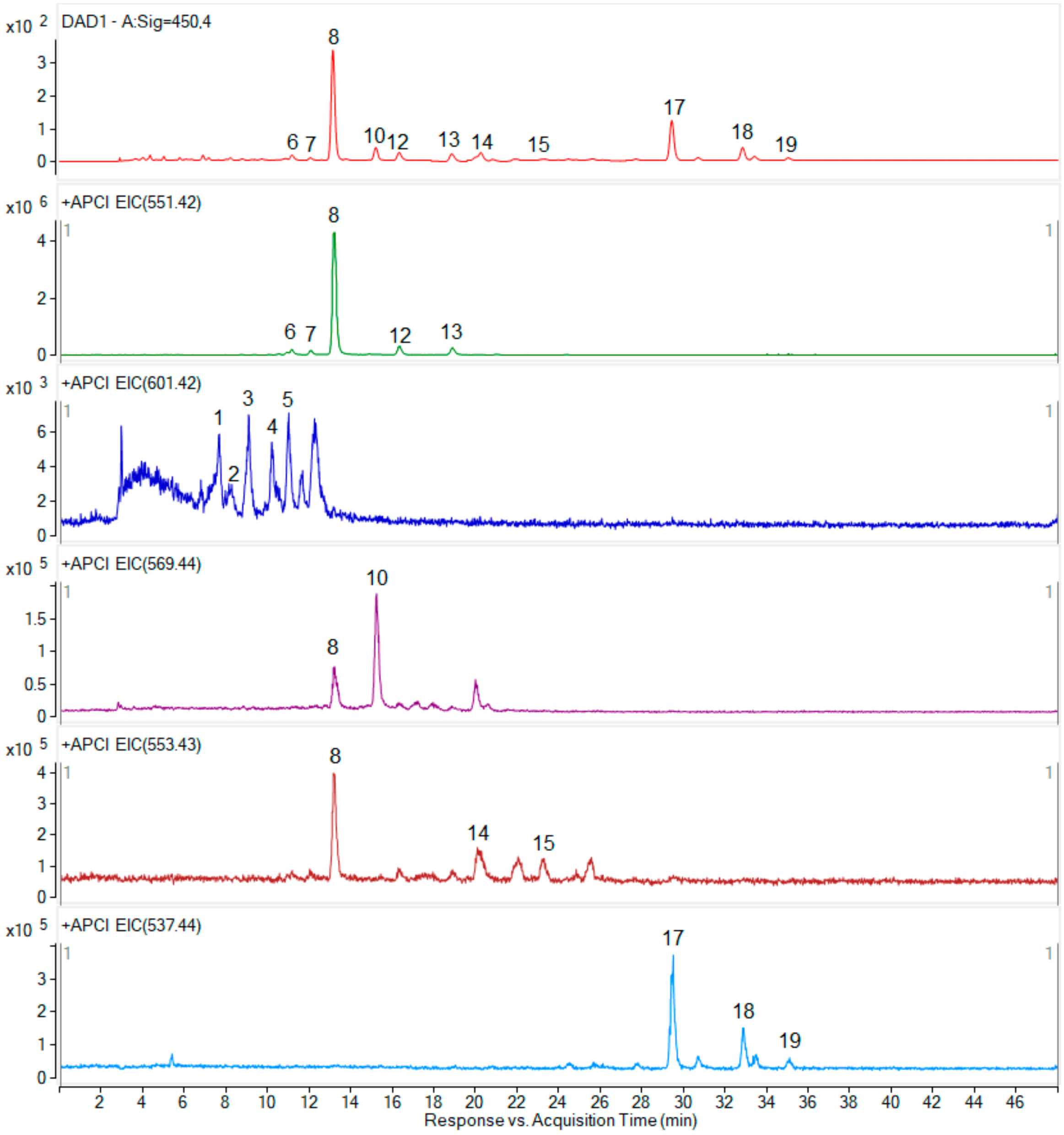

Figure S4.9. UV-vis and EIC chromatogram of *Echinacea paradoxa*
